# The Role of *Spongia* sp. in the Discovery of Marine Lead Compounds

**DOI:** 10.3390/md14080139

**Published:** 2016-07-23

**Authors:** Patrícia Máximo, Luísa M. Ferreira, Paula Branco, Pedro Lima, Ana Lourenço

**Affiliations:** 1 LAQV-REQUIMTE, Departamento de Química, Faculdade de Ciências e Tecnologia, Universidade NOVA de Lisboa, 2829-516 Caparica, Portugal; lpf@fct.unl.pt (L.M.F.); paula.branco@fct.unl.pt (P.B.); 2 Sea4Us—Biotecnologia de Recursos Marinhos, Ltd., 8650-378 Sagres, Portugal; pedro.lima@sea4us.pt; 3 Nova Medical School/Faculdade de Ciências Médicas, Universidade Nova de Lisboa, Campo Mártires da Pátria 130, 1169-056 Lisboa, Portugal

**Keywords:** *Spongia* sp., sesquiterpene quinones, diterpenes, C21 furanoterpenes, sesterterpenes, sterols, macrolides, biological activity

## Abstract

A comprehensive review on the chemistry of *Spongia* sp. is here presented, together with the biological activity of the isolated compounds. The compounds are grouped in sesquiterpene quinones, diterpenes, C21 and other linear furanoterpenes, sesterterpenes, sterols (including secosterols), macrolides and miscellaneous compounds. Among other reports we include studies on the intraspecific diversity of a Mediterranean species, compounds isolated from associated sponge and nudibranch and compounds isolated from *S. zimocca* and the red seaweed *Laurentia microcladia*. Under biological activity a table of the reported biological activities of the various compounds and the biological screening of extracts are described. The present review covers the literature from 1971 to 2015.

## 1. Introduction

Marine sponges have been considered as a very remarkable field for the discovery of bioactive natural products, being so far the most studied source of marine natural products [1]. Some of these metabolites contribute to the chemical defense against predation in their habitat, overgrowth by fouling organisms or competition for space. Moreover, many of them have been found to possess multiple biological activities, such as antitumor, antiviral, anti-inflammatory, immunosuppressive and antibiotic, among others ([1] and previous reviews, [2]). The genus *Spongia*, Linnaeus 1759, belongs to the family Spongidae of the order Dictyoceratida. It comprises three subgenus, *Australospongia*, *Heterofibria* and *Spongia*, containing 1, 7 and 81 species, respectively, according to “The world Porifera database” “and the WoRMS (World Register of Marine Species)”. Knowledge of the softness, elasticity and water retention capacity has rendered some of the species of *Spongia* genus useful as bath sponges [3,4]. As a result of overfishing, habitat degradation and spread of diseases, one of them, *S. agaricina*, is now considered an endangered species under Annex III of the Bern and Barcelona conventions [5]. It is worth mentioning that the nomenclature *S. agaricina* Pallas 1766 has been proposed to refer only to Philippine specimens while the Mediterranean ones should be better referred to as *S. lamella* Schultze 1879 [6]. An interesting study on the potential use of three *Spongia* sp., specially *S. agaricina*, as precursors in the production of ceramic based tissue engineered bone scaffolds has been recently published [7].

Many reports on the chemistry of *Spongia* sp. have been published since 1971 and the work of Fattorusso et al. [8] on the C21 furanoterpenes of *S. nitens*, the first report on the chemistry of *Spongia* sp. The C21 furanoterpenes, together with spongian diterpenes and scalarane sesterterpenoids, are one of the more abundant metabolite structures of this genus. Other metabolites comprise sesquiterpene quinones (mainly with a rearranged drimane skeleton), sterols and secosterols (mainly of the 5α-cholest-7-en and 5α-hydroxy-cholest-7-en type), and macrolides. A section with reports on the isolation of previously unreported compounds and the biological activity for each of these metabolite classes is presented including at the end reports on X-ray structures, reports on the isolation of known compounds and isolated biological activity studies (other studies). A description of the structure assignment is only given for new compounds, since the known metabolites were identified, in most cases, by comparison with literature data. Under Other reports we include studies on the intraspecific diversity of a Mediterranean species, the compounds isolated from associated sponge and nudibranchs (which are believed to sequester sponge compounds) and geographically co-occurring sponge and seaweed (where the opposite occurs). A section on biological activity summarizing the described biological activities of the compounds and the biological screening of extracts is also provided at the end of the chapter. This review covers the literature from 1971 to 2015.

## 2. Sesquiterpene Quinones

**Figure 1:**
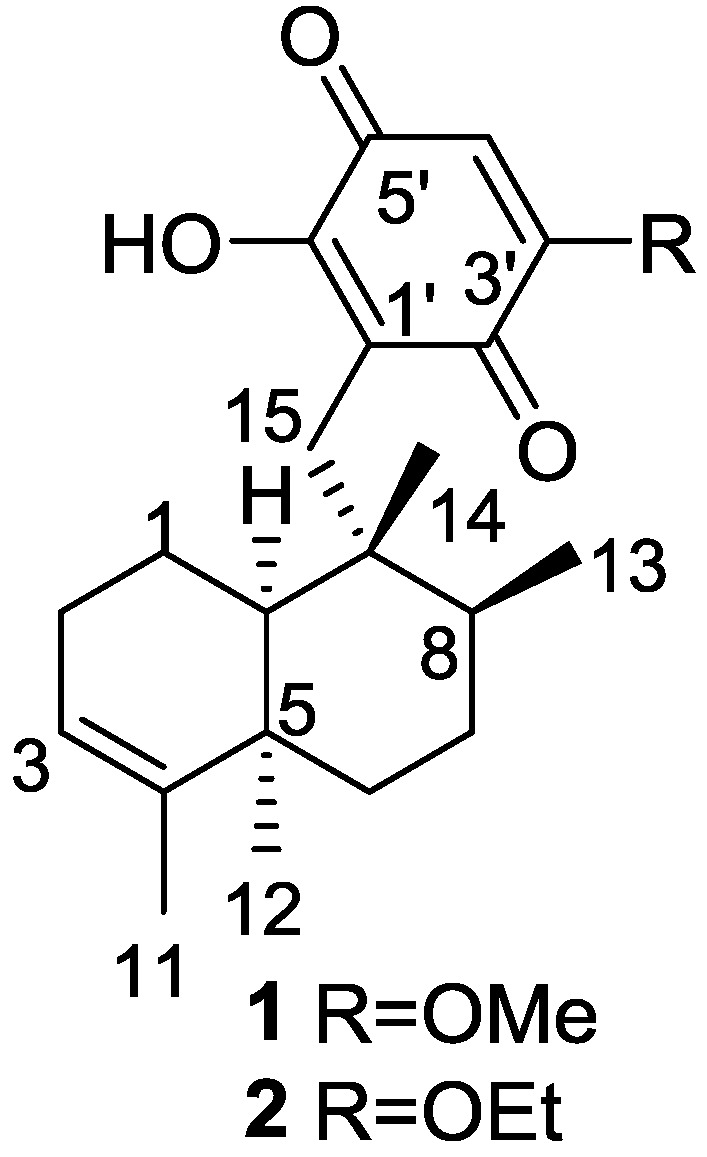
Structures of 5-*epi*-isospongiaquinone **1** and 5-*epi*-homoisospongiaquinone **2**.

Urban and Capon [9] reported the isolation of 5-*epi*-isospongiaquinone **1**, together with 5-*epi*-homoisospongiaquinone **2** (Figure 1), a possible artifact of isolation procedures, from *S. hispida*, collected in the south western coast of Australia.

Compound **1** was identified by comparison with the known isospongiaquinone, the C-5 epimer. The fact that the ^13^C NMR (CDCl_3_) resonance of C-12 (32.3 ppm) was deshielded compared to that of isospongiaquinone (19.9 ppm) was interpreted by the authors as being diagnostic of a *cis*, rather than a *trans* ring junction. Further evidence came from the acid-catalyzed rearrangement of **1** that gave two compounds in all respect identical with the ones obtained from isospongiaquinone. Both **1** and **2** showed antibiotic activity against *Staphylococcus aureus* (MIC 20 μg/disk and 50 μg/disk, respectively) and *Micrococcus* sp. (MIC 20 μg/disk and 50 μg/disk, respectively).

**Figure 2:**
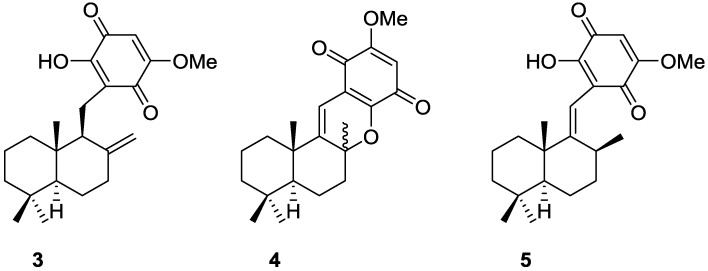
Structures of compound **3**, dehydrocyclospongiaquinone-1 **4** and spongiaquinone **5**.

Subsequent studies by Capon et al. [10] led to the isolation of the new **3**, together with the known dehydrocyclospongiaquinone-1 **4** and spongiaquinone **5** from a *Spongia* sp. collected in the Great Australian Bight (Figure 2). **5** was also isolated as a potassium salt.

Compound **3** was identified by ^1^H and ^13^C NMR after methylation and comparison with known compounds. For spongiaquinone **5** the previously assigned *E* configuration of the double bond was confirmed by nOe, and the depicted absolute stereochemistry was established by chemical degradation. X-ray fluorescence spectroscopy confirmed potassium as the main counter ion (sodium was present in trace amounts). The isolated compounds proved to be responsible for the antibiotic activity of the extract against a range of test microorganisms.

**Figure 3:**
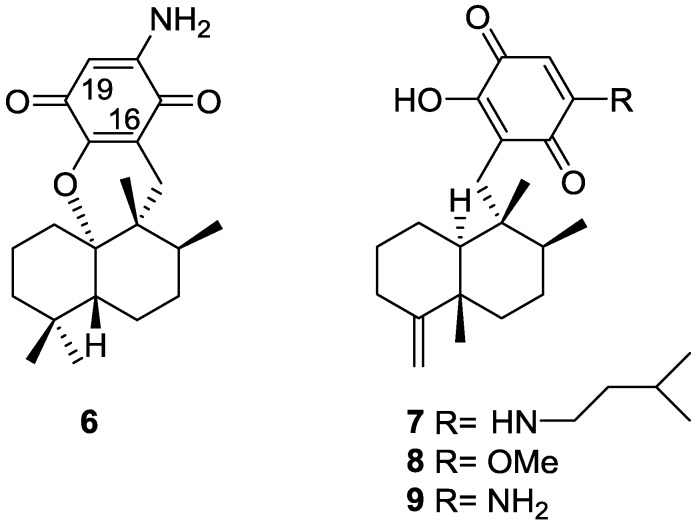
Structures of cyclosmenospongine **6**, smenospongiarine **7**, ilimaquinone **8** and smenospongine **9**.

From a *Spongia* sp. collected in Australia, the isolation of the unusual cyclosmenospongine **6** was reported by Utkina et al. [11], together with the already known metabolites smenospongiarine **7**, ilimaquinone **8** and smenospongine **9** (Figure 3). It is worth mentioning that ilimaquinone had its structure revised in 1987 [12]. Since the absolute configuration of smenospongine **9** was established by comparison of CD spectra with ilimaquinone **8** [13], the structure here presented is also corrected.

For cyclosmenospongine **6** a rearranged drimane skeleton was proposed in the basis of ^1^H and ^13^C NMR data, together with the mass spectra fragment at *m/z* 191. UV and IR spectra indicated the presence of a 1,4-benzoquinone. The bathochromic shift of the absorption maxima observed in the UV spectra together with IR bands confirmed the presence of an amino substituent. A quaternary carbon at δ 88.6 ppm and an IR band at 1244 cm^−1^ confirmed the presence of an ether linkage. Analysis of ^1^H–^1^H COSY, HMQC and HMBC allowed the confirmation of the proposed structure. The relative stereochemistry was ascertained by nOe experiments where irradiation of Me-14 resulted in nOe to H-5 and Me-13. The absolute stereochemistry of **6** was subsequently determined as 5*R*,8*S*,9*R*,10*S* by chemical correlation [14]. Cyclosmenospongine **6** showed moderate cytotoxic activity against mouse Ehrlich carcinoma cells (IC_100_ 145 μM) and moderate hemolytic activity, inducing 50% hemolysis of mice blood erythrocytes at a concentration of 70 μM in 10 min.

**Figure 4:**
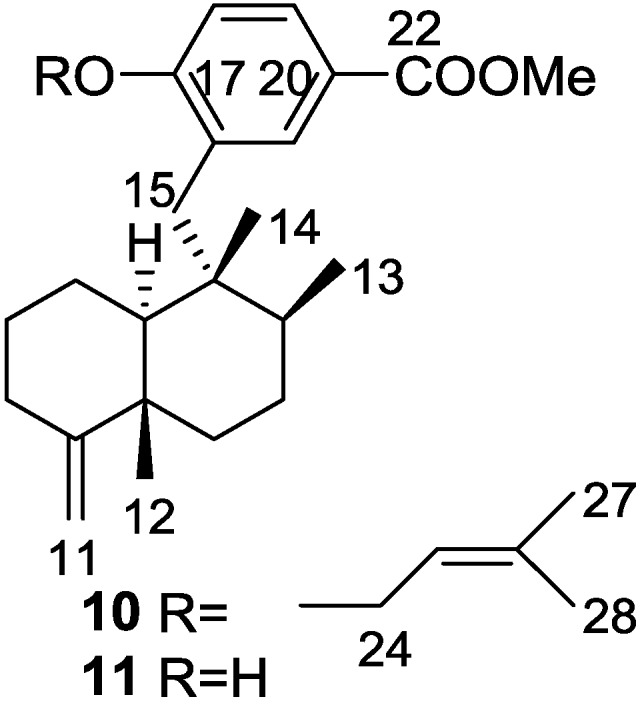
Structures of 17-*O*-isoprenyldictyoceratin-C **10** and dictyoceratin-C **11**.

Cao et al. [15] reported the isolation of the new 17-*O*-isoprenyldictyoceratin-C **10**, together with the known dictyoceratin-C **11** (Figure 4), ilimaquinone **8**, and a nucleoside 2′-deoxyuridine, from a bioactive extract of *Spongia* sp. collected in the Philippines. The extract showed inhibitory activity toward the lyase activity of DNA polymerase β at 16.2 μg/mL.

The structures of **11** and **8** were identified by comparison with literature data and 2′-deoxyuridine with an authentic sample. For **10**, the rearranged drimane skeleton was established by the typical ^1^H NMR signals. The NMR spectra also showed the presence of an 1,2,4-trisubstituted benzene ring (confirmed by UV), and a carbomethoxy moiety (confirmed by IR). The prenyloxy group was identified by the characteristic ^1^H NMR signals. Comparison of the data with that of **11** confirmed **10** as a *O*-prenylated derivative, whose location was confirmed by ROESY (correlation from H-24 to H-18). Stereochemistry was assumed on the basis of the value of the optical rotation of both compounds. Further evidence came from conversion of **11** to **10**. The absolute stereochemistry was not determined although its co-occurrence with ilimaquinone **8** supports the depicted structure. Purified compounds were used to determine the IC_50_ values for inhibition of lyase activity of rat DNA polymerase β, as well as for cytotoxicity to A2780 ovarian cancer cells and inhibitory activity toward Cdc25B. Compounds **10**, **11** and the nucleoside were inactive in all three assays. **8** showed a IC_50_ of 45.2 μM as inhibitor of lyase activity of DNA polymerase β. It was also weakly active as an inhibitor of Cdc25B, with an IC_50_ of 92 μM, and showed moderate toxicity to A2780 cells with an IC_50_ of 10.9 μM.

**Figure 5:**
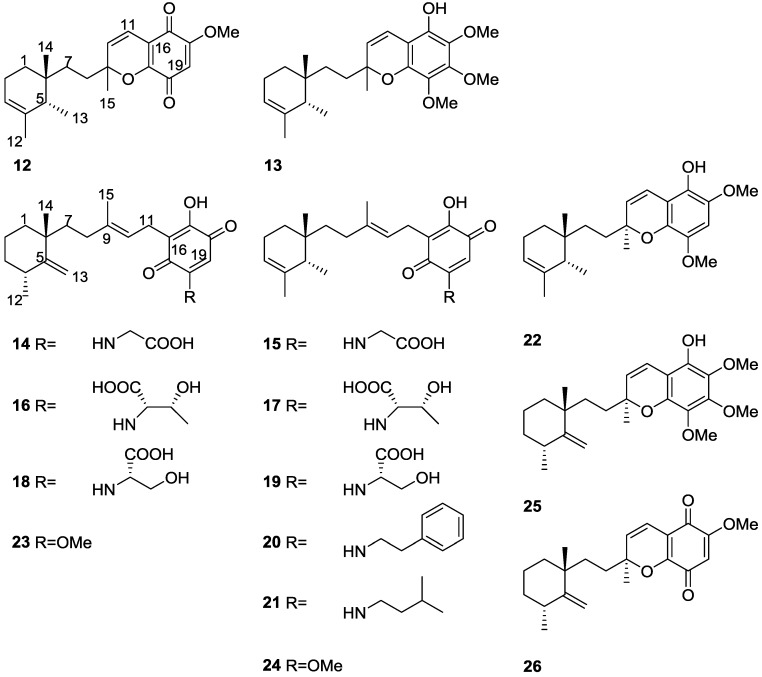
Structures of metachromins J **12** and K **13**, L–T, **14**–**22**, A **23**, and C–E **24**–**26**.

Work of Takahashi et al. [16,17,18] allowed the identification of several new metachromins, metachromins J **12** and K **13**, L–T, **14**–**22**, together with the known metachromins A **23**, C–E **24**–**26**, from a *Spongia* sp. collected in Okinawa (Figure 5).

For metachromin J **12** IR and UV allowed the identification of the carbonyl group and quinone moiety. Comparison of the spectral data with that of metachromins C **24** and E **26** allowed the determination of the proposed structure. NOESY correlations between H-1/Me-13, H-2/Me-14 and H-5/Me-14 allowed the determination of the relative stereochemistry of Me-13 and Me-14 and of a pseudochair conformation for the cyclohexene ring. For metachromin K **13** the hydroxyl group and aromatic ring were identified by IR and UV, respectively. Comparison of the spectral data with that of metachromin C **24**, metachromin D **25** and metachromin J **12** allowed the determination of the structure. Both compounds showed weak cytotoxicity against murine lymphoma L1210 cells (IC_50_ 1.0 and 12.6 μg/mL, respectively) and human epidermoid carcinoma KB cells (IC_50_ 9.9 and >20 μg/mL, respectively) in vitro. For metachromin L **14** the presence of OH and/or NH and carboxy groups was established by IR data. A conjugated carbonyl functionality was also present and the UV spectrum suggested the presence of a quinone chromophore. Similarity of the overall NMR data to metachromin A **23** together with the signals corresponding to a glycine residue led to the assignment. Further confirmation was obtained by chemical synthesis of **14** from **23**. Comparison of the NMR data of metachromins N **16** and P **18** with that of metachromin L **14** led to the assignment of the former. Again, confirmation of the proposed structures came from their synthesis from metachromin A **23**. Metachromins M **15**, O **17** and Q **19** were assigned analogously by comparison of the NMR data with that of metachromin C **24**. Synthesis from this latter compound confirmed the assigned structures. The structure of metachromin R **20** was assigned by IR, UV and NMR data (including ^1^H–^1^H COSY, TOCSY and HMBC). Comparison with the known metachromin G showed that the main differences were the presence of a substituted double bond bearing a methyl group in place of an exomethylene. A phenethylamine unit was inferred from NMR and its connectivity was established by HMBC. The relative stereochemistry of the cyclohexane ring was established by NOESY where cross peaks between H-2β and Me-14 and H-1α and Me-13 were observed. Comparison of the NMR data of metachromin S **21** with that of metachromin R **20**, allowed the assignment of the depicted structure. NOESY revealed that the stereochemistry was the same for both compounds. For metachromin T **22** analysis and comparison of the IR, UV and NMR data (including ^1^H–^1^H COSY, TOCSY and HMBC) with that of metachromin B allowed the identification of a 6,8-dimethoxy-2-methyl-*2H*chromen-5-ol moiety, confirmed by HMBC. Further NMR analysis allowed the identification of the remaining structure, indicating that **22** possessed a cyclohexane ring identical to **20** and **21**. The NOESY spectra of **22** indicated that the stereochemistry of the cyclohexane moiety was the same. The absolute configuration at C-9 was deduced as *S* from CD spectra. For all three compounds **20**–**22** the absolute stereochemistry at C-5 and C-6 was tentatively assigned as *S* and *R*, respectively, since they can be considered to be generated through the same biosynthetic path as metachromin A **23**, whose C-6 configuration is *R*. Metachromins L **14**, M **15**, S **21** and T **22** showed toxicity against L1210 (IC_50_ 4.0, 3.5, 5.2 and 3.0 μg/mL, respectively) and KB cells (IC_50_ 4.0, 5.4, >10 and 5.6 μg/mL, respectively) in vitro, while metachromins N–Q and R, **16**–**19** and **20** did not show that activity (IC_50_ > 10 μg/mL). In a subsequent study [19] metachromin L **14** showed inhibitory activity of EGFR (epidermal growth factor receptor) kinase (IC_50_ 197 μg/mL) and metachromins L–Q, **14**–**19**, showed inhibitory activity of HER2 (human epidermal growth factor receptor 2) kinase (IC_50_ 125, 79, 190, 27, 18 and 22 μg/mL, respectively).

**Figure 6:**
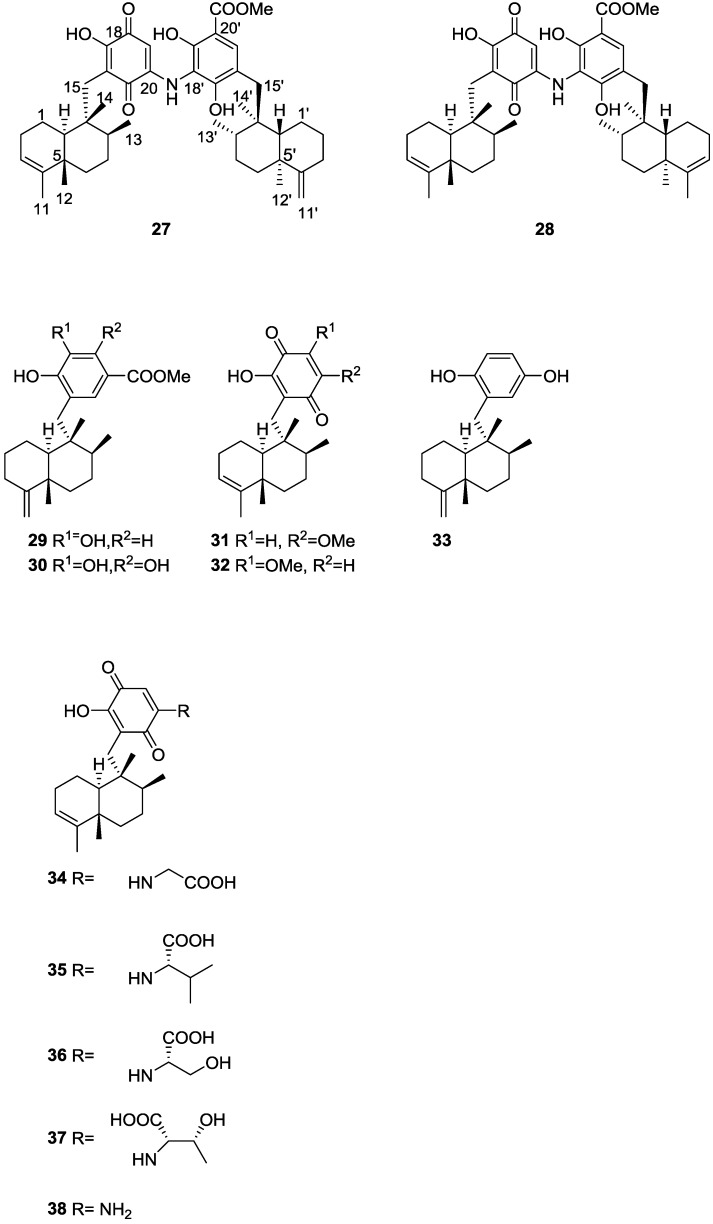
Structures of nakijiquinone E **27** and F **28**, dictyoceratins A–C, **29**, **30**, isospongiaquinone **31**, 6′-hydroxy-4′-methoxyavarone **32**, neoavarol **33**, nakijiquinones A–D **34**–**37**, and an *endo* olefin isomer at C-3 of smenospongine **38**.

Further investigation of another lot of the same sponge by Takahashi et al. [20] afforded the new dimeric sesquiterpenoid quinones, nakijiquinone E **27** and F **28**, together with the known dictyoceratins A–C, **29**, **30** and **11**, isospongiaquinone **31**, 6′-hydroxy-4′-methoxyavarone **32**, neoavarol **33**, nakijiquinones A–D **34**–**37**, and an *endo* olefin isomer at C-3 of smenospongine **38** (Figure 6).

For **27** IR data implied the presence of OH and/or NH, carboxy and conjugated carbonyl functionalities. UV suggested the presence of the quinone chromophore. HRESIMS and ^1^H and ^13^C NMR data suggested a dimeric sesquiterpenoid quinone. Further analysis of NMR spectra, including ^1^H–^1^H COSY and HMBC, identified a tetramethyl decalin with an endo olefin, a trimethyl decalin with an exomethylene, a 2-amino-5-hydroxy-benzoquinone and a methyl 3-amino-2, 4-dihydroxybenzoate. Connection of these moieties was confirmed by HMBC. The relative stereochemistry of the two decaline was established by NOESY. The α-configuration of H-10 and β-configurations of Me-12, Me-13 and Me-14 were deduced from the correlations H-8/H-10, H-10/CH_2_-15 and Me-12/Me-14. Correlations H-8′/H-10′, H-10′/CH_2_-15′ and Me-12′/Me-14′ revealed the same orientation for H-10′ and Me-12′, Me-13′ and Me-14′. **28** possessed similar spectral data to that of **27** the difference being the absence of the exomethylene; NMR analysis established its structure. The relative stereochemistry was assigned by NOESY. Both compounds did not show cytotoxicity against P388 and L1210, and KB cells (IC50 > 10 μg/mL).

### Other Studies

**Figure 7:**
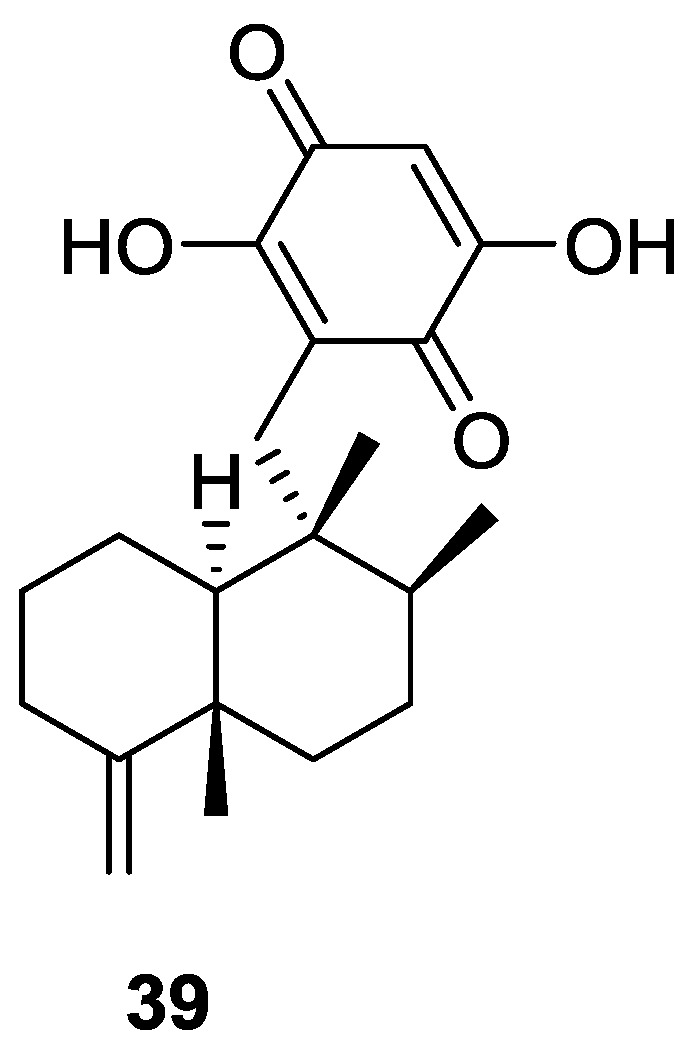
Structure of smenoquinone **39**.

Utkina and Denisenko [21] reported the isolation of the already known smenoquinone **39** (Figure 7), together with smenospongiarine **7** and ilimaquinone **8**, from a *Spongia* sp. collected in the Vietnam sea.

The antioxidant activity of **39** was tested using bleaching of solutions of DPPH radical (2,2-diphenyl-1-picrylhydrazyl radical) and ABTS^•+^ ([2.22-azinobis(3-ethylbenzothiazolin-6-sulfonic acid)]). Compound **39** showed moderate activity from trapping DPPH radicals (IC_50_ 3.7 × 10^−4^ M, comparable to that of ionol, IC_50_ 3.6 × 10^−4^ M). The antioxidant activity for reduction of ABTS^•+^ radical cations corresponded to 0.15 mmol/L of trolox (6-hydroxy-2,5,7,8-tetramethylchroman-2-carboxylic acid) equivalents. Compounds **7** and **8** were inactive.

An independent study by Kittiwisut et al. [22] investigated the antiproliferative activity of several sesquiterpene quinones in a SRB assay. Ilimaquinone **8** showed an IC_50_ of 7.6 μM against HeLa cells and initiated toxicity, in addition to the already mentioned activity.

## 3. Diterpenes

**Figure 8:**
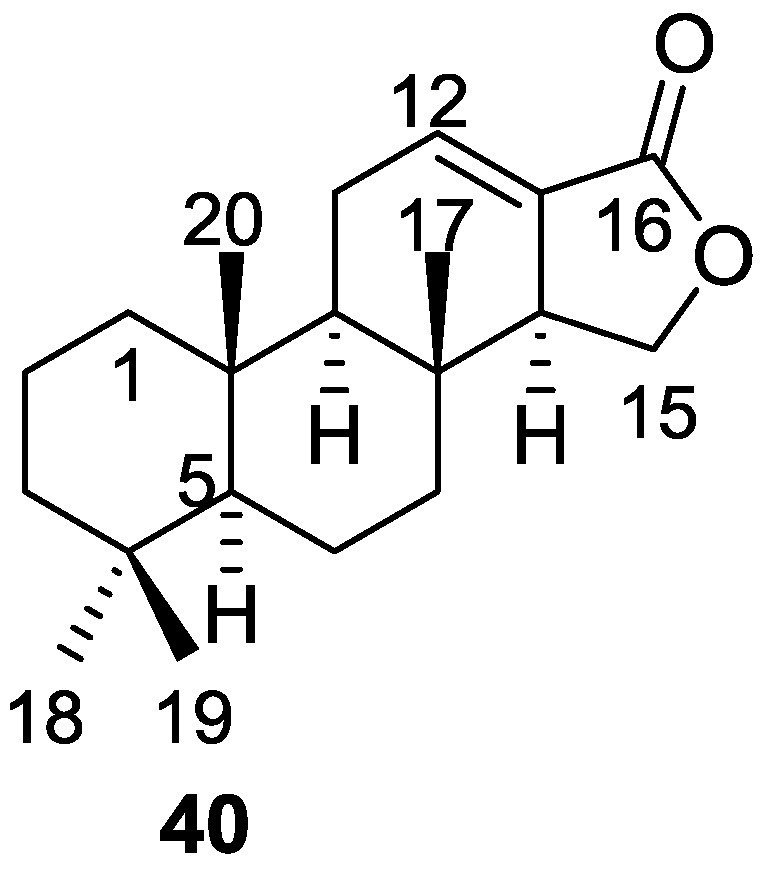
Structure of isoagatholactone **40**.

Cimino et al. [23] reported the isolation of the first spongian diterpene, isoagatholactone **40** (Figure 8) from a *S. officinalis* collected in Naples.

The UV and IR absorptions indicated the presence of a α,β-exounsaturated γ-lactone. ^1^H NMR showed the presence of four tertiary methyls, an olefinic hydrogen coupled with an allylic methylene and an allylic methine (confirmed by decoupling experiments), and an oxygenated methylene coupled to the allylic methine (confirmed by decoupling experiments). MS data where the base peak at *m/z* 192 originating from a retro Diels-Alder process was observed, confirmed the position of the double bond in ring C. Chemical correlation with grindelic acid confirmed the structure and the depicted stereochemistry.

**Figure 9:**
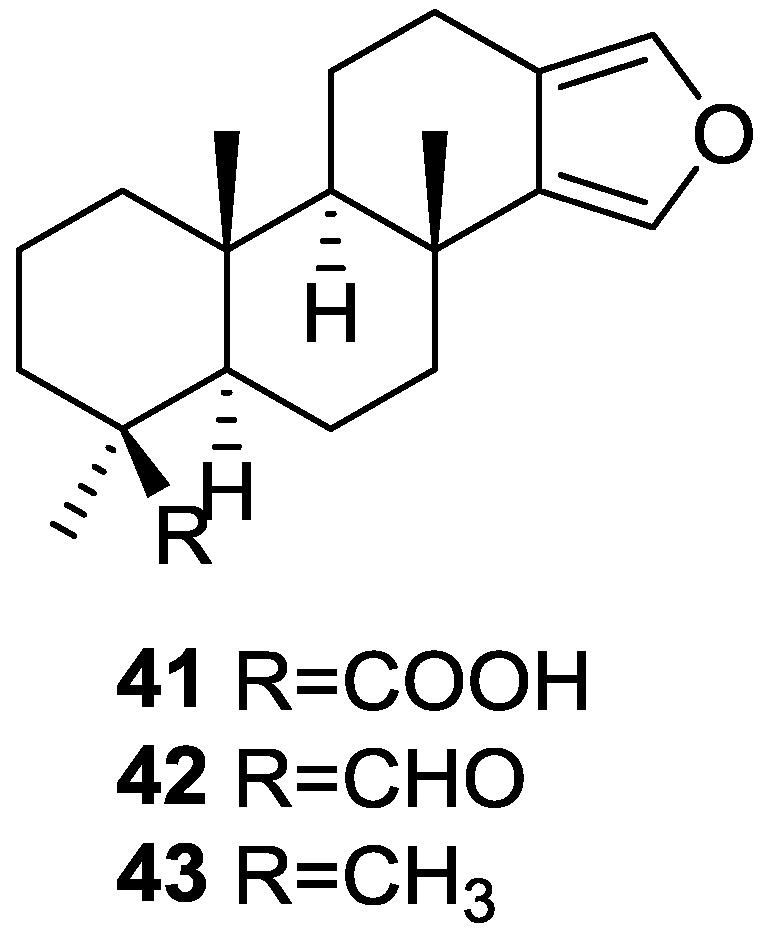
Structures of spongia-13(16),14-dien-19-oic acid **41**, spongia-13(16),14-dien-19-al **42** and spongia-13(16),14-diene **43**.

Capelle et al. [24] reported the isolation of the new spongia-13(16),14-dien-19-oic acid **41**, spongia-13(16),14-dien-19-al **42** and spongia-13(16),14-diene **43** from a *S. officinalis* collected in Papua-New Guinea (Figure 9).

UV, IR, ^1^H NMR and ^13^C NMR data of **41** allowed the identification of a β,β-disubstituted furan moiety, a carboxylic acid and three tertiary methyl groups, compatible with a tetracyclic diterpene with a furan ring (confirmed by comparison with literature compounds). Location of the COOH group was established by pyridine induced shifts in ^1^H NMR. The spectral data of **42** was very similar to that of **41**, with the characteristic signals of the COOH giving rise to an aldehyde function. Reduction of both compounds to the corresponding alcohol confirmed the assignment. For **43** the appearance of a fourth methyl group and absence of the COOH or CHO functions revealed its structure. Confirmation came from chemical correlation with the alcohol obtained by reduction of **41** and **42**.

**Figure 10:**
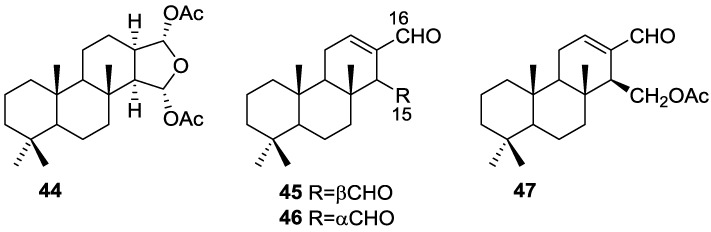
Structures of 15α,16α-diacetoxyspongian **44**, *ent*-isocopal-12-en-15,16-dial **45**, 14-iso-*ent*-isocopal-12-en-15,16-dial **46** and 15-acetoxy-*ent*-isocopal-12-en-16-al **47**.

Cimino et al. [25] reported the isolation of the new 15α,16α-diacetoxyspongian **44**, *ent*-isocopal-12-en-15,16-dial **45**, 14-iso-*ent*-isocopal-12-en-15,16-dial **46** and 15-acetoxy-*ent*-isocopal-12-en-16-al **47** from a *S. officinalis* (Figure 10).

For **44** ^1^H NMR identified the two acetyl groups and corresponding oxygenated methines, and the four tertiary methyls. Comparison of ^13^C NMR data with the known aplysillin (the 12-acetyl analogous, whose relative stereochemistry has been established by X-ray) confirmed the proposed structure. Comparison of the coupling values for H-15 and H-16 in ^1^H NMR in both compounds supports the depicted stereochemistry. Further evidence came from transformation of **44** into the corresponding furan derivative (already known in the literature) by heating in benzene in the presence of catalytic amounts of silica gel. For **45** four methyl group resonances in ^1^H NMR spectra suggested a diterpene skeleton. Other signals include an olefinic proton and two aldehyde groups. Assignment of ^13^C NMR data was accomplished by comparison with literature compounds. Reduction of **45** afforded the diol of known absolute stereochemistry. NMR data of **46** was very similar to that of **45**, pointing to a C-14 epimer. Isomerization of **45** afforded **46**, confirming the proposed structure of the latter. For **47** an α,β-unsaturated aldehyde, an ester function and an oxygenated methylene could be identified. Reduction with LAH afforded the corresponding diol, confirming structure and absolute stereochemistry.

**Figure 11:**
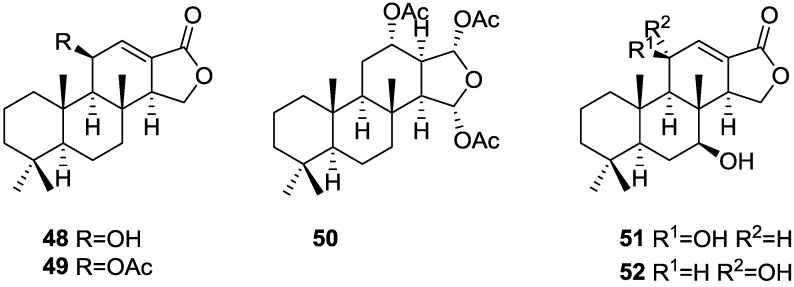
Structures of 11β-hydroxyspongi-12-en-16-one **48**, 11β-acetoxyspongi-12-en-16-one **49**, aplysillin **50**, 7β,11β-dihydroxyspongi-12-en-16-one **51**, and 7β,11α-dihydroxyspongi-12-en-16-one **52**.

Gonzalez et al. [26], from the active methanol extract of *S. officinalis* L. from Tenerife, reported the isolation of the active 11β-hydroxyspongi-12-en-16-one **48** and 11β-acetoxyspongi-12-en-16-one **49**, the inactive and already known isoagatholactone **40** and aplysillin **50** (respectively spongia-12-en-16-one and 12α,15α,16α-triacetoxyspongian), and a mixture of the new 7β,11β-dihydroxyspongi-12-en-16-one **51** and 7β,11α-dihydroxyspongi-12-en-16-one **52** (Figure 11), for which no testing was performed. The extract showed antimicrobial activity against *Staphylococcus aureus*, *Pseudomonas aeruginosa* and *Bacillus sphaericus*, and inhibited HeLa cells with values of ID_50_ 1–5 μg/mL.

For **48** and **49**, a spongian skeleton was inferred from ^13^C NMR data. The presence of an oxygenated substituent at C-11 was established by chemical transformation: acetylation of **48** gave **49**; treatment of **48** with TosCl/Py gave the 9,13-diene as a result of concomitant dehydration; and oxidation of **48** with Jones reagent gave the 11-ketolactone. The stereochemistry at C-11 in **48** and **49** was obtained by ^1^H NMR analysis of hydrogenated derivatives of **49** (cis and trans ring C/D junction) and confirmed by X-ray analysis of a pyrazine derivative obtained by treatment of **49** with diazomethane in ether. The structures of **51** and **52** are proposed on the basis of ^1^H NMR spectra analysis of the pyrazine derivatives obtained from the acetylated natural products.

**Figure 12:**
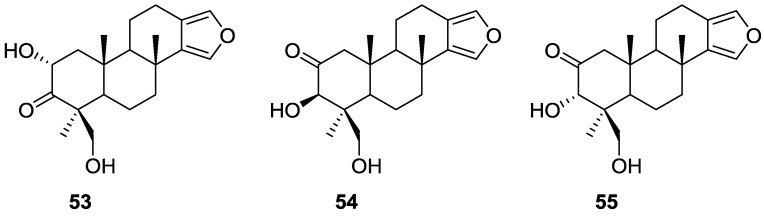
Structures of 2α,19-dihydroxyspongia-13(16),14-dien-3-one (isospongiadiol) **53**, **54** (epispongiadiol) and **55** (spongiadiol).

Kohmoto et al. [27] reported the isolation of the new 2α,19-dihydroxyspongia-13(16),14-dien-3-one (isospongiadiol) **53**, together with the known **54** (epispongiadiol) and **55** (spongiadiol) from a *Spongia* sp. collected in the Bahamas (Figure 12). Previous biological screening of the extract showed activity against HSV-1 (herpes simplex virus type 1) and P388 murine leukemia cells.

Compounds **54** and **55** were identified by comparison with literature data. For **53** ^1^H NMR showed the presence of the furan ring, three methyl singlets and an oxygenated methylene. Comparison of the remaining NMR data with that of **54** and **55** suggested a different oxidation pattern in ring A. Further NMR analysis, including C–H correlations, COSY and nOe established the structure. nOe between H-2 and Me-20, and Me-17 and Me-20 suggested a 1,3 diaxial relationship between these substituents and a chair conformation for ring A. The ring A oxidation pattern and absolute configuration was confirmed by comparison of the ^1^H NMR spectrum and optical rotation of the reduction products of all three compounds. From in vitro assays against P388 cells **53**, **54** and **55** yielded IC_50_ values of 5, 8, and 0. 5 μg/mL, respectively (the value for vinblastine is 0.01 μg/mL). Against HSV-1 the IC_50_ values for **53**, **54** and **55** were 2, 12.5, and 0.25 μg/mL, respectively (the values for ara-A and acyclovir are 50 and 0.5 μg/mL, respectively).

**Figure 13:**
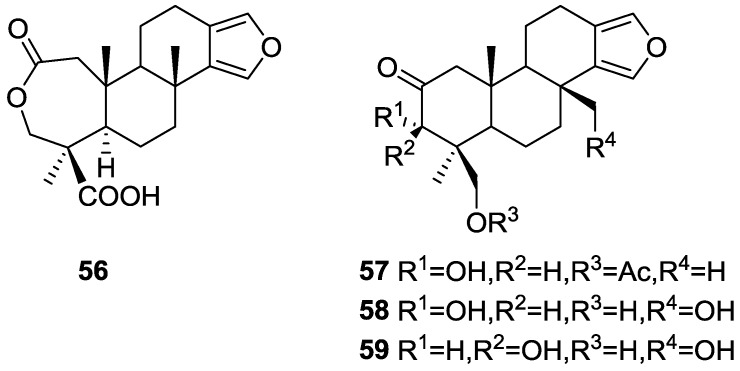
Structures of spongialactone A **56**, 19-acetoxy-3α-hydroxyspongia-13(16),14-dien-2-one **57**, 3α-17,19-trihydroxyspongia-13(16),14-dien-2-one **58**, and 3β,17,19-trihydroxyspongia-13(16),14-dien-2-one **59**.

Hirsch and Cashman [28] reported the isolation of the new spongialactone A **56** and 19-acetoxy-3α-hydroxyspongia-13(16),14-dien-2-one **57** from *S. officinalis* var. *arabica* collected in gulf of Eilat, together with the known metabolites 3α-17,19-trihydroxyspongia-13(16),14-dien-2-one **58** and 3β,17,19-trihydroxyspongia-13(16),14-dien-2-one **59** (Figure 13). **57** is the acetate of the already known diol 3α,19-dihydroxyspongia-13(16),14-dien-2-one

For compounds **57**, **58** and **59** comparison with literature data confirmed their structures. Furthermore, hydrolysis of **57** gave the known parent diol. Comparison of the NMR data of **56** with that of **57** indicated that these compounds only differed in ring A. The carbonyl of **57** was replaced with two new carbonyls, one belonging to an acid group (proved by methylation and acetylation), and the other to a lactone ring (proved by analysis of IR data). The confirmation of the proposed structure for **56**, together with the stereochemistry at C-4 (Me-18 and COOH-19) and C-5 was obtained by NMR experiments (COSY 45, RELAY and NOEDS). The relative configuration of the remaining chiral centers was established by comparison of the NMR data with reference compounds.

**Figure 14:**
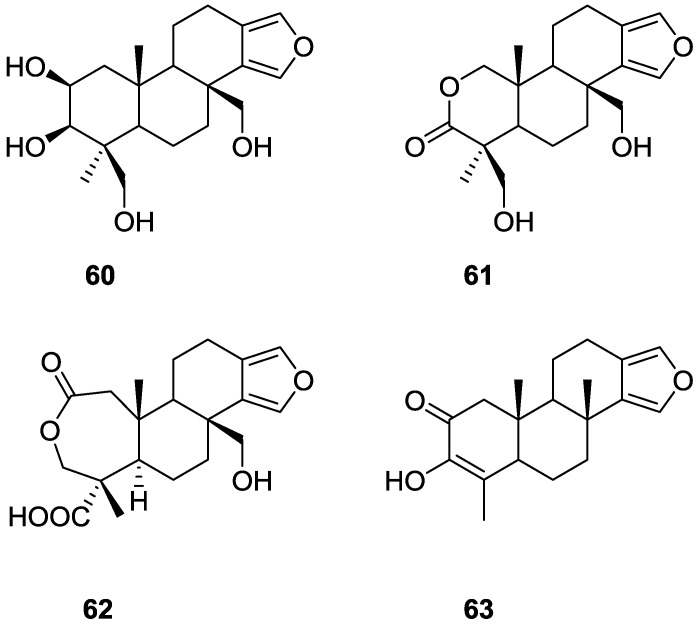
Structures of 2β,3β,17,19-tetrahydroxyspongia-13(16),14-diene **60**, 2-oxa-17,19-dihydroxyspongia 13(16),14-dien-3-one **61**, 17-hydroxy-4-*epi*-spongialactone A **62**, and 19-nor-3-hydroxyspongia-3,13(16),14-trien-2-one **63**.

Gunasekera and Schimtz [29] reported the isolation of four new metabolites 2β,3β,17,19-tetrahydroxyspongia-13(16),14-diene **60**, 2-oxa-17,19-dihydroxyspongia 13(16),14-dien-3-one **61**, 17-hydroxy-4-*epi*-spongialactone A **62** and 19-nor-3-hydroxyspongia-3,13(16),14-trien-2-one **63** (Figure 14), together with the known 3β,17,19-trihydroxyspongia-13(16),14-dien-2-one **59**, from an unidentified *Spongia* sp., collected in Dalton Reef, Australia.

The identification of **59** was accomplished by comparison with literature data, although some comments were made. Confirmation of the β-orientation of C-3-OH (inferred from the ^13^C shift value) came from irradiation of the signal of H-3 that sharpened one of the signals of H-19, showing that H-3 and the oxymethylene group are diaxially disposed. For the remaining compounds the spongiane skeleton, rings B, C and D, two quaternary methyls and the C-17 substituent were established by comparison of the ^1^H and ^13^C NMR data with that of **59**. For **60** the absence of the carbonyl in IR suggested a reduction derivative of **59**. nOe established the β-orientation of C-17, C-19, Me-20 (irradiation of Me-20) and α-orientation of H-3 (irradiation of Me-18). The latter was confirmed by the upfield shift of α-H-1, excluding a 1,3-diaxial relationship with C-3-OH. The configuration at C-2 was inferred from the *J* coupling values of H-2. For **61** NMR data analysis and nOe (enhancement of CH_2_-17, CH_2_-19 and H-1 upon irradiation of Me-20) led to the proposed structure. The authors suggest the presence of an intramolecular hydrogen bond between the C-19-OH and the carbonyl group, based on the low frequency observed for the latter in IR (1702 cm^−1^). Compound **62** was purified and identified after esterification with diazomethane and acetylation of C-17-OH. Decoupling experiments and nOe (enhancement of Me-19 and CH_2_-17 upon irradiation of Me-20, and enhancement of CH_2_-3 upon irradiation of Me-19) confirmed the structure. For **63**, confirmation of the structure came from coupling of the vinyl methyl with H-5. Compound **61** showed marginal cytotoxicity to murine leukemia cells (P388), E_50_ = 3.5 μg/mL, and the other compounds were inactive.

**Figure 15:**
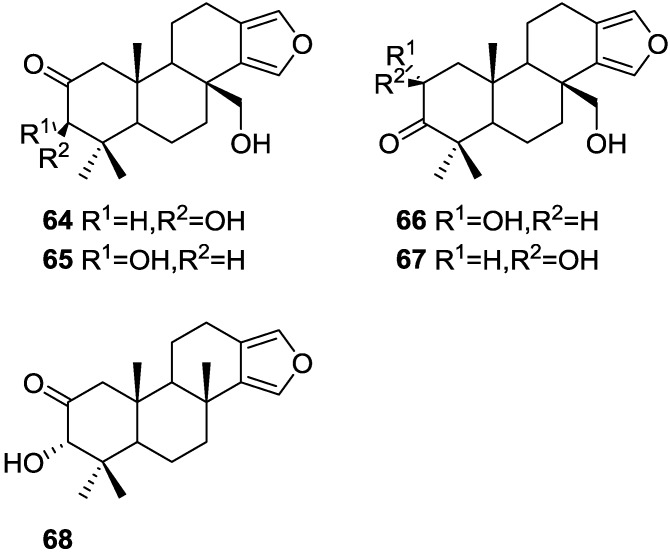
Structures of 3β,17-dihydroxyspongia-13(16),14-dien-2-one **64**, 3α,17-dihydroxyspongia-13(16),14-dien-2-one **65**, 2α,17-dihydroxyspongia-13(16),14-dien-3-one **66**, 2β,17-dihydroxyspongia-13(16),14-dien-3-one **67**, and 3α-hydroxyspongia-13(16),14-dien-3-one **68**.

Searle and Molinski [30] reported, among others, the isolation of 5 new diterpenes, 3β,17-dihydroxyspongia-13(16),14-dien-2-one **64**, 3α,17-dihydroxyspongia-13(16),14-dien-2-one **65**, 2α,17-dihydroxyspongia-13(16),14-dien-3-one **66**, 2β,17-dihydroxyspongia-13(16),14-dien-3-one **67** and 3α-hydroxyspongia-13(16),14-dien-3-one **68** (Figure 15), together with the known spongia-13(16),14-diene **43**, from a *Spongia* sp. collected in Australia.

**43** was identified by comparison with literature data. For **64**, analysis of NMR data, including COSY, HETCOR and COLOC spectra established an oxidized tetracyclic spongian diterpene skeleton and allowed structural identification. Comparison of the furan ^13^C chemical shifts with those of reported compounds allowed the hydroxymethylene group to be placed at C-17, supported by COSY and COLOC experiments. A chair conformation for A ring with equatorial C-3-OH and Me-18 was established by NOEDS spectra. NMR analysis of **65** identified it as a C-3 epimer of **64**. Strong nOe observed between H-3 and Me-20 established a boat conformation for ring A, with C-3-OH in a *pseudo*-equatorial “prow” position. NMR analysis of **66** and **67** and comparison with **64** and **65** established their structures and allowed their identification as C-2 epimers. NOESY experiments established the relative configuration of **66** and suggested a chair conformation for ring A, with the C-2-OH in an equatorial position. Both **66** and **67** proved to be rather unstable, which prevented full characterization. For **68**, analysis of the NMR data and comparison with **64**, **65** and literature compounds established its structure. The absolute configuration of **64** was established as 2*S*,3*R* by CD studies of the tribenzoate derivative obtained by esterification of the 2β,3β,17β-triol obtained after reduction of the C-2-carbonyl group. This configuration corresponds to the normal “5α,10β” absolute configuration common to all sterols and most polycyclic diterpenes with the exception of *ent* kaurenes, and is consistent with the findings for earlier spongian derivatives.

**Figure 16:**
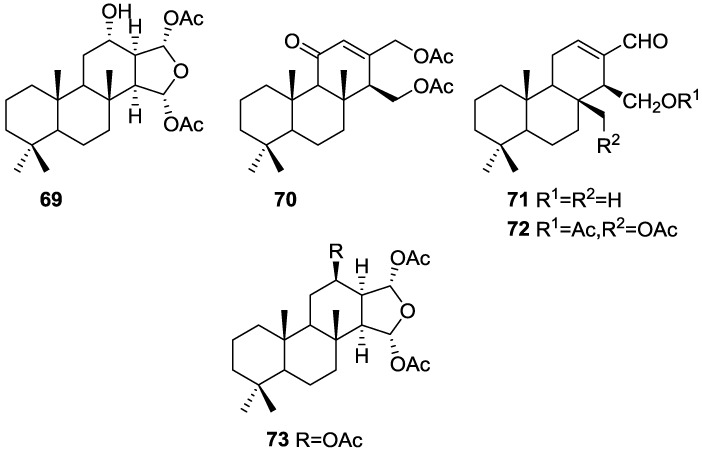
Structures of 12-deacetyl-aplysillin **69**, 15,16-diacetoxy-11-oxo-*ent*-isocopal-12ene **70**, 15-hydroxy-*ent*-isocopal-12-en-16-al **71**, 15,17-diacetoxy-*ent*-isocopal-12-en-16-al **72**, and compound **73**.

Zubía et al. [31] reported the isolation of four new metabolites 12-deacetyl-aplysillin **69**, 15,16-diacetoxy-11-oxo-*ent*-isocopal-12ene **70**, 15-hydroxy-*ent*-isocopal-12-en-16-al **71**, 15,17-diacetoxy-*ent*-isocopal-12-en-16-al **72**, and seven already known structures **40**, **47**, **49**, **44**, **73**, **46** and **45**, from a Mediterranean sponge, *S. zimoca*, Schmidt 1862, collected in the channel of Sicily (Figure 16).

The known compounds were identified by comparison with literature data while the new compounds were identified by comparison of the NMR spectra with those of **50**, **72** and **47**. Acetylation of **69** afforded a compound in all respects identical with **50** (including optical rotation). The absolute stereochemistry at C-12 was ascertained by applying a modified Mosher’s method. Attempts to acetylate **71** to obtain **47** failed, probably because of the existence of an intramolecular hydrogen bond between C-15-OH and the aldehyde group. Alternatively methanolysis of **47** with Na_2_CO_3_/MeOH (anhydrous) afforded **71**. Analysis and comparison of the NMR data of **72** with that of **47** allowed the identification of the former, where the chemical shift of C-7 was diagnostic to localize an acetoxy group at C-17. Compound **70** was identified by comparison with **72** and NMR data analysis. The multiplicity of the olefinic proton allowed the correct localization of all the functionalities. The relative stereochemistry at C-14 was supported by comparison of the ^13^C NMR δ value for C-7, similar to that reported for **47**. The authors suggest that most probably all the metabolites have the same absolute stereochemistry as **40**. This suggestion has been proven for **69** (Mosher’s method) and is supported by the fact that **45**, **47** and **72** show CD curves opposite to the curves of known compounds (polydiglyal, scalaradial and 12-deacetoxy-scalaradial) supporting the *ent*-isocopalane skeletons.

**Figure 17:**
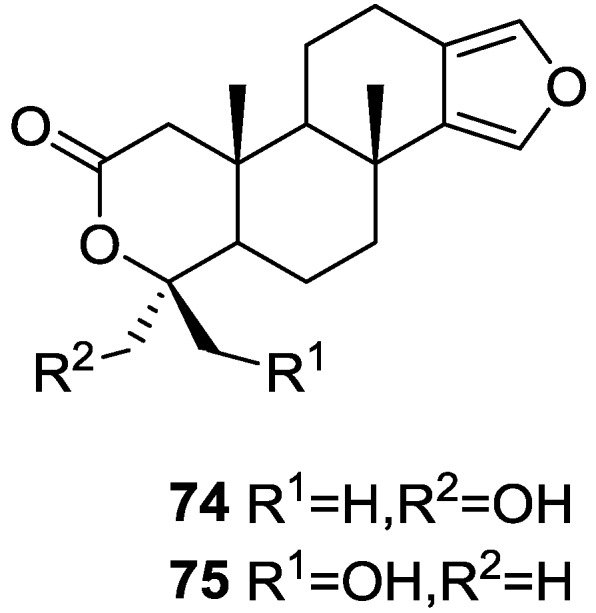
Strutures of furanoterpenes **74** and **75**.

Li et al. [32] reported the isolation of two new metabolites **74** and **75** (Figure 17) together with the previously reported **53**, **54**, **63** and a furanoterpene, from the sponge *S. matamata* de Laubenfels, 1954, collected in Yap island, Micronesia. This specimen was later reclassified as *S. zimocca sensu* de Laubenfels by the same authors in a subsequent study [33].

The known compounds were identified by comparison of the spectral data with the literature. The new compounds were identified by ^1^H, ^13^C, HMQC, HMBC and nOe experiments and comparison with **54**, **53** and **63**, that showed that they only differed in ring A. The A/B ring *trans* fusion of **74** was established by comparison of the ^13^C NMR δ value of Me-20 with those of **54** and **53** and by the coupling constant of H-5. The α-configuration of the oxymethylene was determined on the basis of the nOe enhancement of the oxymethylene proton signals after irradiation of H-5. The β-orientation of the oxymethylene in **75** was confirmed by the nOe enhancement of its signal upon irradiation of Me-20. Analysis of the ^1^H NMR signals of the these protons before and after proton exchange pointed to the existence of a restricted conformation in which there is significant coupling between only one of the methylene protons and the hydroxyl proton. The authors suggest this is due to a hydrogen bond between the hydroxyl and one of the lactone oxygens. The brine shrimp lethality test was carried out for the purified compounds: **74** was inactive, and the remaining compounds showed mild toxicity with LC_50_ values of approximately 50–100 μg/mL.

**Figure 18:**
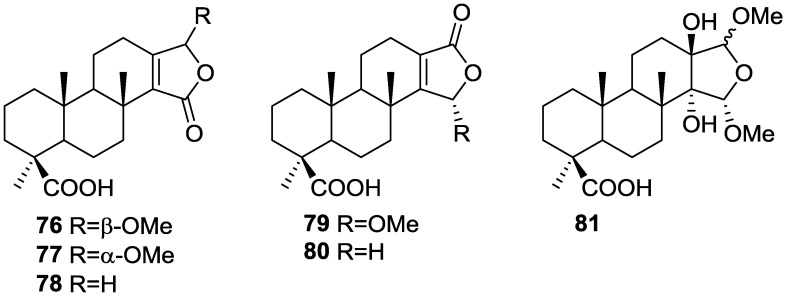
Strutctures of 16β-methoxy-15-oxospongi-13-en-19-oic-acid **76**, 16α-methoxy-15-oxospongi-13-en-19-oic-acid **77**, 15-oxospongi-13-en-19-oic acid **78**, 15α-methoxy-16-oxospongi-13-en-19-oic-acid **79**, 16-oxospongi-13-en-19-oic acid **80**, and 13β,14α-dihydroxy-15α,16ξ-dimethoxyspongian-19-oic-acid **81**.

In the subsequent study of *S. matamata* de Laubenfels collected in Yap island, Micronesia, from the same authors [33] six new terpenoids, 16β-methoxy-15-oxospongi-13-en-19-oic-acid **76**, 16α-methoxy-15-oxospongi-13-en-19-oic-acid **77**, 15-oxospongi-13-en-19-oic acid **78**, 15α-methoxy-16-oxospongi-13-en-19-oic-acid **79**, 16-oxospongi-13-en-19-oic acid **80**, 13β,14α-dihydroxy-15α,16ξ-dimethoxyspongian-19-oic-acid **81** (Figure 18) and the known spongia-13(16),14-dien-19-oic acid **41** were isolated.

The known metabolite **41** was identified by comparison with literature data. Comparison of ^1^H and ^13^C NMR data of the new compounds with **41** showed that all compounds had identically substituted rings A and B. Confirmation of these rings stereochemistry was obtained from pyridine-induced solvent shifts. For **76** the NMR data indicated the presence of a carbonyl group, a tetrasubstituted double bond, methoxyl and acetal functions. An α,β-unsaturated-γ-lactone was identified by UV and IR and confirmed by HMBC. The stereochemistry at C-16 of **76** was established on the basis of NOESY of H-16 with H-12α. Although a similar study could not be performed with **77** due to overlapping of the two allylic protons at C-12, the similarity of spectral data allowed its identification as an epimer of **76**. For **79** a α,β-unsaturated-γ-lactone with acetal function was confirmed by HMBC. Irradiation of Me-17 in NOESY studies caused an enhancement of H-15, confirming the α-orientation of the methoxyl group. The structure of **81** was established by HMBC data. NOESY experiments confirmed the α-orientation of the C-14-OH and C-15-OMe groups. The deshielding effect on Me-17 on running the NMR spectra in pyridine proved that C-13-OH is β-oriented. The configuration at C-16 could not be resolved. NMR analysis of **78** and **80** indicated the presence of an α,β-unsaturated-γ-lactone (confirmed by UV and IR data) in ring D. The downfield shift of C-14 in **80** when compared to **78** confirmed that this was the β-carbon of the an α,β-unsaturated-γ-lactone and made possible the distinction of both compounds. The brine shrimp lethality test was carried out for all the purified compounds but **76**. Only **41** showed mild toxicity, with an LC_50_ value 10–100 μg/mL.

**Figure 19:**
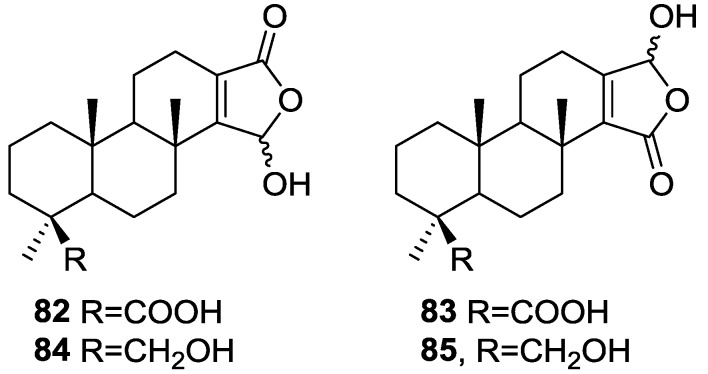
Structures of spongiabutenolides A–D, **82**–**85**.

Mitchell et al. [34] reported the isolation of four new diterpenes, spongiabutenolides A–D, **82**–**85** (Figure 19), together with the known spongia-13(16),14-dien-19-oic acid **41**, from a sample of *Spongia* sp. collected in the Philippines.

Each of the new structures consisted of an inseparable mixture of stereoisomers at the hemiacetal carbon. Furthermore, structures **82** and **83** had to be separated as their methyl esters in order to be identified. The natural products were eventually separated and their spectral data was obtained. The structures were identified by ^1^H, ^13^C, HMQC, HMBC and 1D-TOCSY NMR spectra. The relative stereochemistry of the **82** (and **83**) was established by ROESY correlations of **82** and its methyl ester. Correlations were seen between Me-20/Me-17/COOMe and Me-18/H-5. The relative stereochemistry of **84** was established by ROESY spectra, while that of **85** was assumed. **82** and **83** were synthesized by singlet oxygen oxidation starting from **41**. All the compounds were tested for anti-cancer activity in a 25 cell-line panel but none showed significant cytotoxicity.

**Figure 20:**
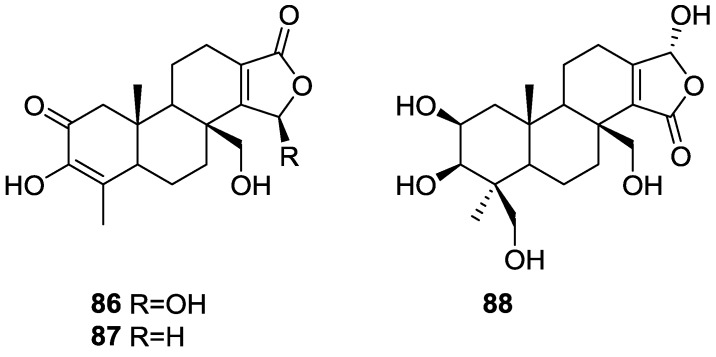
Structures of zimoclactone A **86**, zimoclactone B **87**, and zimoclactone C **88**.

Zeng et al. [35] and Su et al. [36] reported the isolation of the new zimoclactone A **86**, zimoclactone B **87** and zimoclactone C **88** from *S. zimocca* subspecies *irregularia* (Figure 20).

The structures were determined by 1D and 2D NMR and X-ray diffraction analysis. Zimoclactone A **86** was isolated with 7-dehydrocholesterol and showed moderate cytotoxic activity against P388 cells.

**Figure 21:**
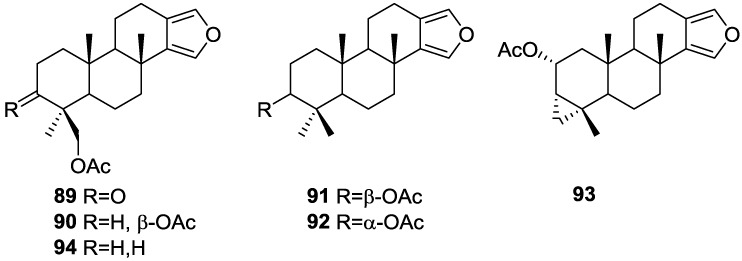
Structures of 19-acetoxyspongia-13(16),14-dien-3-one **89**, 3β,19-diacetoxyspongia-13(16),14-diene **90**, 3β-acetoxyspongia-13(16),14-diene **91**, 3α-acetoxyspongia-13(16),14-diene **92**, 2(*R*),3(*S*),4(*S*)-3,18-methylene-2α-acetoxyspongia-13(16),14-diene **93**, and 19-acetoxyspongia-13(16),14-diene **94**.

Ponomarenko et al. [37], isolated five new diterpenes, 19-acetoxyspongia-13(16),14-dien-3-one **89**, 3β,19-diacetoxyspongia-13(16),14-diene **90**, 3β-acetoxyspongia-13(16),14-diene **91**, 3α-acetoxyspongia-13(16),14-diene **92** and 2(*R*),3(*S*),4(*S*)-3,18-methylene-2α-acetoxyspongia-13(16),14-diene **93**, together with the known 19-acetoxyspongia-13(16),14-diene **94**, from *S. Heterofibria* collected in Northern Cook Islands (Figure 21).

Structure of **89** was established on the basis of ^1^H, ^13^C, COSY, HSQC and HMBC and a single crystal X-ray diffraction study, followed by CD spectroscopy—the conformations of A and B rings are chairs, while that of ring C is a half chair. The CD spectrum of **89** showed positive Cotton effects, and application of the octant rule established the depicted stereochemistry (4*S*,5*R*,8*R*,9*R*,10*R*), in accordance with other spongians diterpenoids. Alkaline hydrolysis afforded the known 19-hydroxyspongia-13(16),14-dien-3-one, although the observed and reported values for optical rotation where somewhat different. Comparison of the NMR data of the remaining compounds with that of **89** established the spongian-based furanoditerpene skeleton. It was suggested that all these metabolites shared with **89** the same absolute configurations in their polycyclic structures. The nature and orientation of the substituents was established by NMR data analysis (including ^1^H–^1^H COSY, HSQC, HMBC, and NOESY spectra, and irradiation experiments). For **90** the configuration at C-3 came from the *J* coupling value of H-3 and its NOESY with H-5 and Me-18. NOESY of CH_2_-19 with Me-20 established the β-orientation of the former. For **91** and **92** the orientation of the acetoxy group was inferred from the *J* coupling values of H-3, and NOESY with H-5 and Me-18 in the case of **91**. The unusual cyclopropane ring in **93** was identified by the high field ^1^H NMR signals and its location in C-3, C-4 and C-18 was established on the basis of the HMBC spectra. Its α-orientation was established by nOe of one H-18 with H-5. The α-orientation of the acetoxy group at C-2 was established by the *J* coupling observed for H-2 and its nOe with Me-20. Compounds **90** and **94** were tested for immunomodulatory properties by the methods reported in the literature and demonstrated a slight lysosomal activation (about 130% of control) of mice spleenocytes at concentrations of 100 μg/mL.

**Figure 22:**
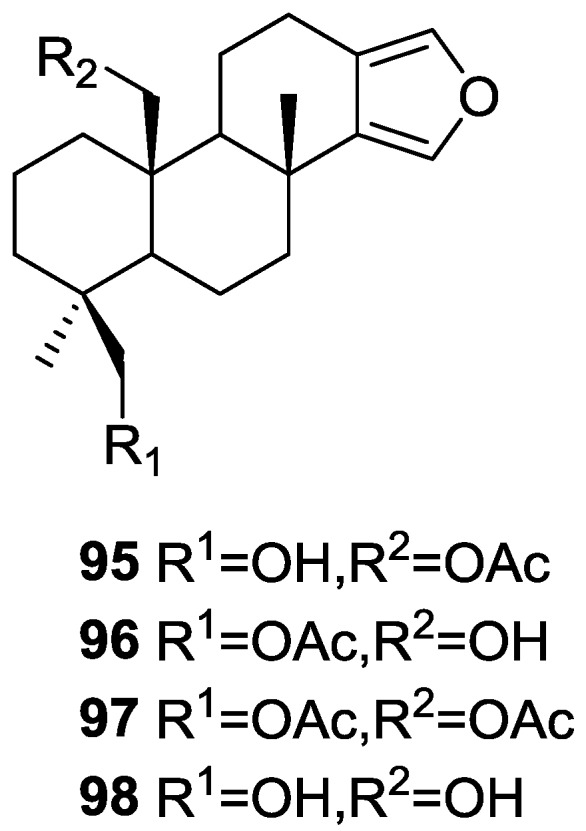
Structures of 20-acetoxy-19-hydroxyspongia-13(16),14-diene **95**, 19-acetoxy-20-hydroxyspongia-13(16),14-diene **96**, 19,20-diacetoxyspongia-13(16),14-diene **97**, and 19,20-dihydroxyspongia-13(16),14-diene **98**.

Carroll et al. [38] reported the isolation of four new spongian diterpenes 20-acetoxy-19-hydroxyspongia-13(16),14-diene **95**, 19-acetoxy-20-hydroxyspongia-13(16),14-diene **96**, 19,20-diacetoxyspongia-13(16),14-diene **97** and 19,20-dihydroxyspongia-13(16),14-diene **98** (Figure 22) together with the known spongia-13(16),14-diene **43**, from an extract of *Spongia* sp. collected in Wreck Reef, Coral Sea, that showed TRH-R2 binding affinity.

The known compound was identified by 2D NMR data analysis and comparison with literature data. For the remaining compounds analysis of the ^1^H, ^13^C, gCOSY, gHMQC, HMBC and ROESY spectra allowed their full characterization. All four compounds showed positive Cotton effects in their CD spectra, confirming the 4*S*,5*R*,8*R*,9*R*,10*S* configurations. For **95** Me-18 was established as equatorial on basis of the chemical shift. ROESY correlations between CH_2_-19 and CH_2_-20 confirmed a 1,3-diaxial relationship. For **96,** comparison with **95** revealed an isomeric relationship. HMBC confirmed the structure. For **97** the comparison with **95** and **96** led to the proposed structure. For **98** the lack of the acetate band in IR, of the corresponding methyl signal in ^1^H NMR and the upfield shifts of CH_2_-20 when compared to **95** led to the proposed structure. TRH is a tripeptide that has been proposed to play an important role in neurotransmitter signaling. Two subtypes of the TRH receptor, TRH-R1 and TRH-R2 are found in rat brain tissues. Agonists and antagonist of TRH binding show potential therapeutic value in regulating endocrine function, in controlling pain, and in the treatment of spinal cord injury. Compound **95** was the most active of the five compounds in the TRH-R2 receptor binding assay, exhibiting an IC_50_ of 23 μM. Compounds **96**, **97**, **98** and **43** were only weakly active, displaying IC_50_’s of 70 μM, 400 μM, 600 μM and 1 mM, respectively. The reference compound TRH had an IC_50_ of 23 nM.

**Figure 23:**
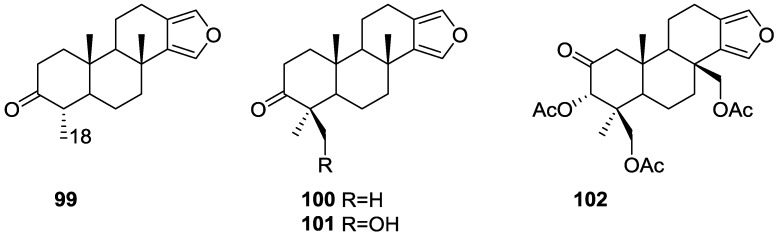
Structures of 19-norspongia-13(16),14-dien-3-one **99**, and compounds **100**–**102**.

Ponomarenko et al. [39] reported the isolation of the new 19-norspongia-13(16),14-dien-3-one **99**, together with the known **93**, **102**, **100**, **91**, **92**, **94**, **89**, **90** and **101** from a *Spongia* ssp. (subgenus *Heterofibria*) collected in Northern Cook Islands. From a *Spongia* ssp. (subgenus *Heterofibria*) collected in Vietnam the known **43** and **91** were isolated. Compound **99** had previously been synthesized (Figure 23).

MS and ^13^C NMR data for **99** suggested a norditerpenoid structure. In the NMR spectra, signals corresponding to the furan ring, two methyls at quaternary carbons, one methyl group at a tertiary carbon and a carbonyl group were observed. HMBC confirmed that only one methyl was attached to C-4. nOe enhancements of H-4 and Me-17 upon irradiation of Me-20 proved that H-4 is β-oriented. The effects of **89**, **90**, **91**, **99**, **100**, **101** and **102** on the biosynthesis of nucleic acids and embryonic development of the sea urchin *Strongylocentrotus intermedius* were studied. All the compounds inhibited sea urchin embryo development at concentrations of 20 μg/mL and above and DNA biosynthesis at the dose of 10 μg/mL. The inhibitory effect of these diterpenoids may partly be explained by the inhibition of thymidine kinase activity. The same compounds stimulated RNA synthesis in the developing sea urchin embryos.

**Figure 24:**
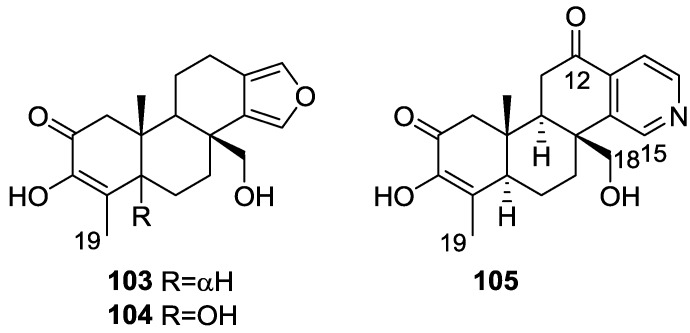
Structures of 18-nor-3,17-dihydroxyspongia-3,13(16),14-trien-2-one **103**, 18-nor-3,5,17-trihydroxyspongia-3,13(16),14-trien-2-one **104**, and spongiapyridine **105**.

Parrish et al. [40] reported the isolation of three new diterpenes 18-nor-3,17-dihydroxyspongia-3,13(16),14-trien-2-one **103**, 18-nor-3,5,17-trihydroxyspongia-3,13(16),14-trien-2-one **104** and spongiapyridine **105** (Figure 24) together with the known **62**, from an unidentified *Spongia* sp. collected in Sulawesi, Indonesia.

**Figure 25:**
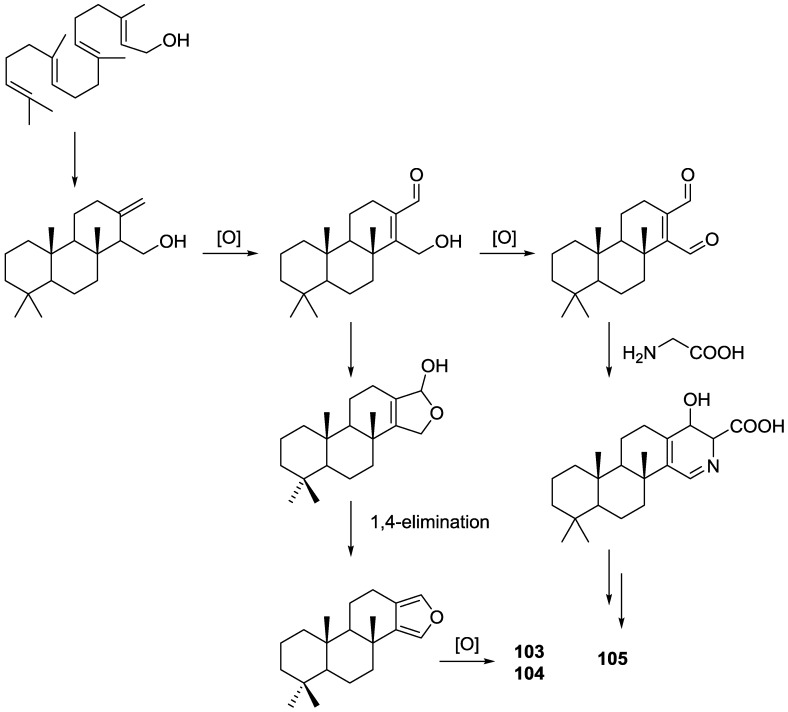
Proposed biosynthesis route for compounds **103**, **104** and **105**.

Structure of compound **103** was established on the basis of ^1^H, ^13^C, HMBC and COSY NMR spectra. The relative configuration was ascertained by ROESY spectra where correlations between Me-20 and CH_2_-17 indicated they were *syn* diaxial. Correlation between H-9 and H-5 (axial, on the basis of *J* coupling values with H-6) identified H-9 as axial. The presence of the 5-OH substituent in **104** was suggested by the downfield shift of C-5 when compared to **103**, and confirmed by HMBC. Of the four stereocenters of **104** only two could be determined by NOESY: Me-20 and CH_2_-17 in a *syn* diaxial relationship. The coupling constant of H-9 indicated it was axial as well. C-5 could not be determined due to rapid exchange of the alcoholic proton in aprotic solvents, and at lower temperatures. For compound **105** comparison with **103** showed identical rings A and B. ^1^H and ^13^C NMR data analysis, including ^1^*J*_C–H_ values for H-16, were consistent with the presence of a pyridine ring, which was confirmed by ^1^H–^15^N HMBC. Additional structural features were deduced based on HMBC correlations that connected the pyridine ring to ring B, and indicated that the carbonyl was at C-12. The relative configuration were established by NOESY: again a correlation between Me-20 and CH_2_-18 confirmed both substituents as *syn* diaxial; ^1^H *J* values for H-5 and H-9 suggested both to be axial oriented. For **62**, NMR data analysis was in agreement with the known structure, including the configuration of C-4. Although this is not a new structure full ^1^H and ^13^C NMR data are presented since the former characterization was for the 17-acetyl methyl ester derivative [29]. Since all the spongian diterpenes for which absolute configurations were determined belong to the same enantiomeric series, the authors suggest that all the compounds in this study have the 5*R*,8*R*,9*R*,10*R* configuration. The authors also propose a biosynthetic route to compounds **103**, **104** and **105** (Figure 25).

Several bioactivity tests were performed in search of the chemopreventive capacity of the isolated compounds. Modest inhibition of TNF-α-activated NF-κB activity was observed for **62**, **103**, **104** and **105** with ED_50_ values around 50 μM. No significant activity was observed for inhibition of iNOS activity in LPS-induced RAW 264.7 murine macrophage cells, and no significant induction occurred in a retinoic X receptor response element luciferase reporter gene assay. Compound **104** inhibited aromatase in a dose-dependent manner with an IC_50_ value of 34.4 μM. The other compounds did not achieve 50% inhibition at a concentration of 50 μM. **104** was also tested as an QR1 (NAD(P)H: quinone reductase 1) inducer. With cultured Hepa 1c1c7 cells **104** showed a CD (concentration required to double the specific activity) value of 11.2 μM, which is similar to the CD value of resveratrol (21 mM), a weak QR1 inducer. None of the compounds showed any significant activity towards the aspartic protease BACE1 (<100 μM).

**Figure 26:**
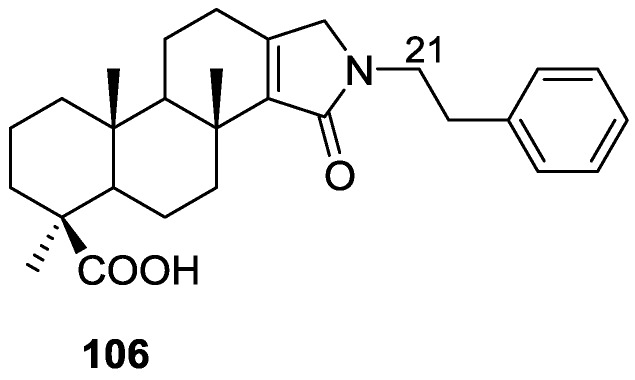
Structure of haumanamide **106**.

Pham et al. [41] reported the isolation of an unusual nitrogenous spongian metabolite, haumanamide **106**, together with the known spongia-13(16),14-dien-19-oic acid **41** from a *Spongia* sp. collected in Pohnpei, Micronesia (Figure 26).

The structure of **106** was established by comparison of the ^13^C NMR data with that of **41**. The α,β-unsaturated-γ-lactam in D ring was confirmed by the chemical shift of C-15, an IR band at 1665 cm^−1^, and HMBC spectrum analysis. Difference nOe measurements confirmed that the relative stereochemistry of both compounds is the same. **106** showed activity against KB (MIC 5 μg/mL) and LoVo (MIC 10 μg/mL) cancer cells.

**Figure 27:**
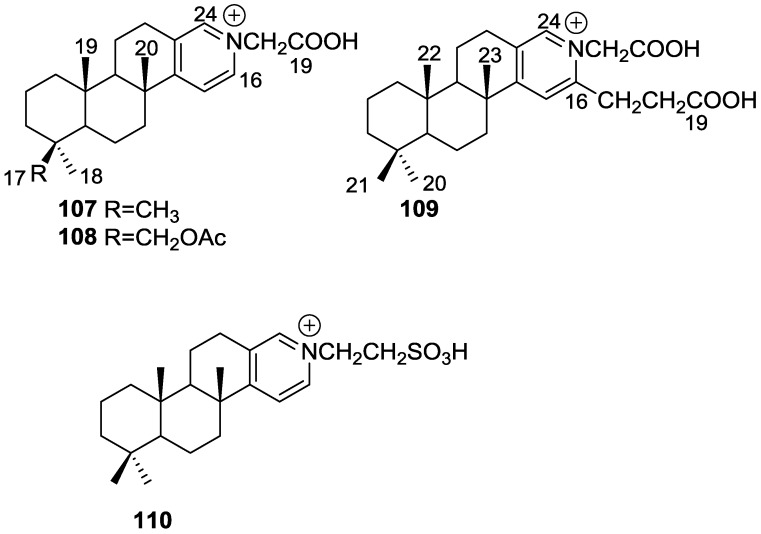
Structures of spongidines A–D **107**–**110**.

De Marino et al. [42] reported the isolation of the new spongidines A–D **107**–**110** from a *Spongia* sp. collected in Vanuatu Islands, Australia (Figure 27).

For **107**, mass spectrum, IR and ^13^C NMR data indicated the presence of a carboxyl group. The ^13^C NMR data also revealed a tricarbocyclic skeleton with geminal dimethyl groups at C-4, and two methyl groups at the ring junctions C-8 and C-10. A disubstituted pyridinium salt was also inferred from ^13^C NMR, confirmed by UV and IR absorptions typical of alkylpyridinium salts. COSY and HMBC allowed the proposal of the structure. For **108** the comparison with **107** allowed the identification of the acetoxymethyl group, located at C-4 by the downfield shift observed at C-4 and upfield shift observed at C-3. The stereochemistry at C-4 was determined by ROESY (intense cross peaks between CH_2_-17 and Me-19). For **109**, ^1^H and ^13^C NMR, COSY and HMBC data, together with comparison with **107** and **108** allowed the determination of the proposed structure. For **110** the comparison with **107** and the differences observed for the pyridine salt moiety, together with COSY and IR data allowed the determination of the taurine residue. HMBC established its location. Inhibition of specific PLA_2_ enzymes constitutes a potentially useful approach for treating a great variety of inflammatory disorders. Compounds **107**, **108**, **109** and **110** were tested as inhibitors of sPLA_2_ (secretory phospholipase A_2_) enzymes belonging to the groups I (*Naja naja* venom and porcine pancreatic enzymes), II (human synovial recombinant and rat air pouch secretory enzymes) and III (bee venom enzymes). All compounds inhibited human synovial PLA_2_ at 10 μM, compound **110**, containing a sulfonic acid group, being the most interesting inhibitor. In this regard these compounds can offer new structural requirements for further studies about mechanistic interactions between PLA_2_ enzymes and inhibitors. All compounds were inactive to cPLA_2_. The results are summarized in Table 1.

**Figure 28:**
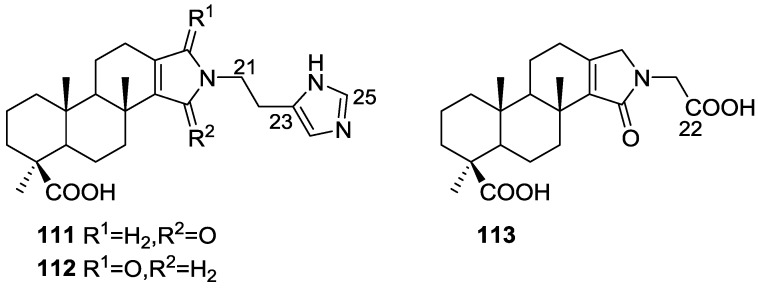
Structures of spongolactams A–C, **111**–**113**.

Mori et al. [43] reported the isolation of spongolactams A–C, **111**–**113** (Figure 28), together with the known spongia-13(16),14-dien-19-oic acid **41**, from a *Spongia* sp. collected in Okinawa, Japan, whose extract showed a 70% inhibition of FTase (Farnesyl transferase) at 20 μg/mL, in a new assay described by the authors.

Structure elucidation for all compounds was based on ^1^H, ^13^C, HMQC, HMBC and DQF-COSY spectra. For compounds **111** and **112** the carboxyl group was identified by its δ value in ^13^C NMR and IR bands; its location was determined by HMBC. Similar process led to identification and localization of the tertiary amide. The 5-imidazolyl ring connected to the C-22 methylene group was established by its typical chemical shifts and HMBC data. The relative stereochemistry of **111** was inferred from NOESY spectra where correlation between H-12/H-16 specified the direction of the lactam group. For **112** the reversal of the chemical shifts of C-15 and C-16, together with NOESY between H-7/H-15 confirmed the structure. Compound **113** was identified by comparison with **111**: significant differences in the spectra were the absence of the imidazole moiety and the replacement of the C-22 methylene by a carbonyl group. The structure and absolute stereochemistry of **111** and **112** were confirmed by synthesis from **41**. The structure of **113** was also confirmed by synthesis from the same precursor. The synthesis of other spongolactam related compounds are also presented. FTase inhibitors are believed to be candidates for novel chemotherapeutic drugs. In the FTase inhibition assays the synthetic sample of **111** showed an IC50 23 mM (natural sample 22 mM). The activity of spongolactams B and C was determined only with synthetic samples due to inadequate amounts of natural material (130 μM and >260 μM, respectively). Cytotoxicity of these compounds against a human vulval-derived epidermoid carcinoma cell line, A431, was also evaluated and apparently some correlation exists between the two assays. The authors suggest that FTase could be a molecular target in the expression of spongolactam cytotoxicity.

### Other Studies

**Figure 29:**
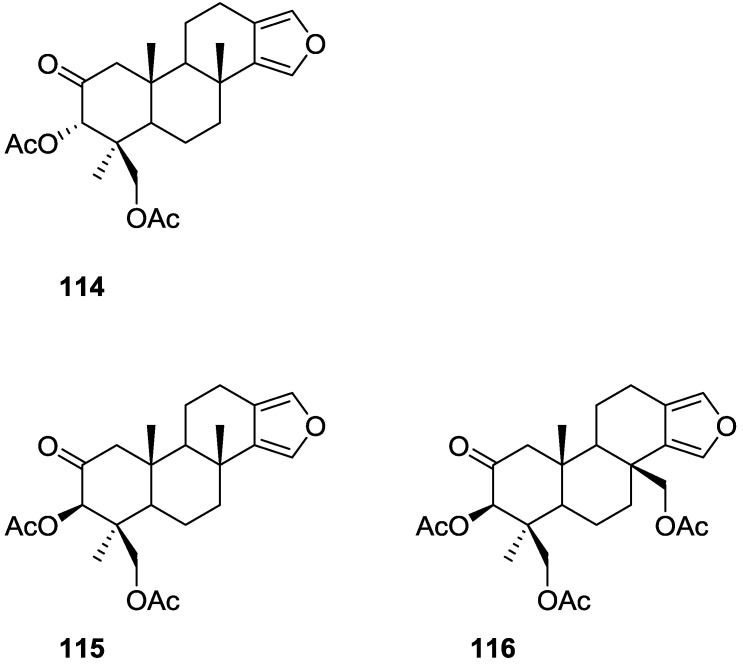
Structures of 3α,19-diacetoxyspongia-13(16),14-dien-2-one **114**, 3β,19-diacetoxyspongia-13(16),14-dien-2-one **115**, and 3β,17,19-triacetoxyspongia-13(16),14-dien-2-one **116**.

Kazlauskas et al. [44] reported the isolation of 3α,19-dihydroxyspongia-13(16),14-dien-2-one **55**, 3β,19-dihydroxyspongia-13(16),14-dien-2-one **54**, 3α,17,19-trihydroxyspongia-13(16),14-dien-2-one **58** and, 3β,17,19-trihydroxyspongia-13(16),14-dien-2-one **59**, together with their acetyl derivatives 3α,19-diacetoxyspongia-13(16),14-dien-2-one **114**, 3β,19-diacetoxyspongia-13(16),14-dien-2-one **115**, 3α,17,19-triacetoxyspongia-13(16),14-dien-2-one **102** and 3β,17,19-triacetoxyspongia-13(16),14-dien-2-one **116** from several *Spongia* sp. collected in the Great Barrier Reef (Figure 29). These specimens were subsequently reclassified as *Rhopaloeides odorabile* [45].

For **114** fragment ions in mass spectra indicated successive losses of CH_3_, AcOH and 2xAcOH. The ^1^H NMR spectrum indicated three quaternary methyls, two acetoxy methyls, an oxygenated methylene and methine and two furan protons. For **102** mass spectra showed the successive losses of CH_2_OAc and AcOH, which suggested that a quaternary methyl group had been replaced by an acetoxymethyl. This was confirmed by ^1^H NMR, where the remaining signals were very similar in both compounds. IR and ^13^C NMR showed the presence of a ketone group for both compounds. Analysis of the ^13^C NMR spectra of both compounds allowed the identification of the furan ring and establishment of the functionality at C-17. ^1^H NMR analysis together with biogenetic considerations established ring A. The position of an acetoxymethyl group at C-4 was assigned for **114** by an ^1^H NMR study in the presence of Eu(fod)_3_. Definite proof of stereochemistry came from a single crystal X-ray diffraction study of **102** where ring A was shown to be present as a boat conformation with atoms C-1, C-2, C-4 and C-5 coplanar, ring B formed a chair, ring C a distorted half-chair and ring D was practically flat. CD and ORD established the absolute configuration of **102**. Acetylation of **59** produced **116** identified as a C-3 epimer of **102**. Acetylation of **54** produced **115**, the C-3 epimer of **114**. Compound **58** was also acetylated to give **102**. Acetylation of a mixture of **55** and **54** gave **114** and **115**.

Puliti and Matia [46] determined the relative configuration of *ent*-isocopal-12-en-15,16-dial **45** as 5S*,8R*,9R*,10S*,14S* by X-ray analysis. A *trans* fused tricyclic system with four methyl substituents, three of which are axially β-oriented (at C-4, C-8, and C-10) was determined. The β-orientation of the aldehyde substituent at C-14 was confirmed.

An independent study by Yong et al. [47] determined the absolute configurations and conformations of **100**, **54** and **55** by X-ray analysis. For **100** a twisted-boat ring A, chair B and C rings and a planar furan ring was determined, with an absolute stereochemistry of 5*R*,8*R*,9*R*,10*R*. In **54** ring A adopts a chair conformation and the hydroxymethylene group donates an intramolecular hydrogen bond to the C-3-OH. An absolute stereochemistry of 3*R*,4*S*,5*R*,8*R*,9*R*,10*R* was determined. For **55** a disordered ring A with a dominant chair conformer with the C-3-OH in an axial position was observed. The minor contribution was a distorted-boat conformer where the hydroxyl group adopts an equatorial position. An absolute stereochemistry of 3*S*,4*S*,5*R*,8*R*,9*R*,10*R* was determined. The authors point out that all the literature spongian diterpenes for which absolute stereochemistry had been reported belonged to the same enantiomeric series, even though some configurations had been assigned by Mosher esters analysis or CD data (as is the case of **64**, **89**, **95** and **102**).

An independent study by Betancur-Galvis et al. [48] tested several spongian diterpenes for their activity against herpes simplex virus type 2 and cytotoxic effect on tumor cells. Compound **40** showed low cytotoxicity and **43** was poorly active against HSV-2. Compound **100** showed no anti viral activity but was cytotoxic to HeLa (human cervix epithelioid carcinoma-CC_100_ 30 μg/mL), Hep-2 (human larynx epidermoid carcinoma-CC_100_ 40 μg/mL), CHO (*Cricetulus griseus* Chinese hamster ovary cells ATCC CCL-61-CC_100_ 30 μg/mL) and Bon-Fib (primary culture of bovine ear subcutaneous fibroblasts-CC_100_ 40 μg/mL) cells.

## 4. C21 and Other Linear Furanoterpenes

**Figure 30:**
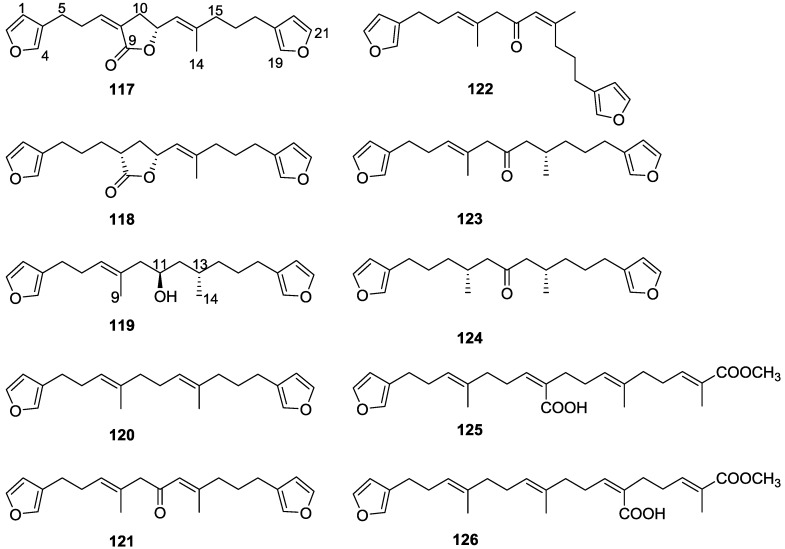
Structures of nitenin **117**, dihydronitenin **118**, furospongin-1 **119**, anhydrofurospongin-1 **120**, furospongin-2 **121**, isofurospongin-2 **122**, dihydrofurospongin-2 **123**, tetrahydrofurospongin-2 **124**, furospongin-3 **125**, and furospongin-4 **126**.

Work of Fattorusso et al. [8] and Cimino et al. [49,50,51] on the chemistry of *S. nitens* and *S. officinalis*, both from the Mediterranean, allowed the isolation and characterization of the furanoterpenes, nitenin **117** and dihydronitenin **118** (both from S. nitens), furospongin-1 **119**, anhydrofurospongin-1 **120**, furospongin-2 **121**, isofurospongin-2 **122**, dihydrofurospongin-2 **123**, tetrahydrofurospongin-2 **124**, furospongin-3 **125** and furospongin-4 **126** (from *S. officinalis*) (Figure 30).

The structures were identified on the basis of UV, IR, ^1^H NMR data with double irradiation experiments, mass spectra, and chemical transformation and degradation. Typical of the furan moiety seem to be a positive Ehrlich test, a λ_max_ ca. 220 nm in UV (cyclohexane), characteristic IR bands at 3140, 1570, 1500, 875 and 780 cm^−1^, ^1^H NMR (CCl_4_) signals at δ 7.26–7.15 ppm and δ 7.14–7.05 ppm for the α-protons of both rings (usually equivalent), one proton signal at δ 6.16–6.14 ppm for the β-protons of both rings (usually equivalent), and mass fragments at *m/z* 67, 81 and 95. The isoprene unit is usually recognized by a methyl singlet at δ 1.58 ppm, broadened by long range coupling to the *trans* olefinic proton. For nitenin **117** and dihydronitenin **118** the configuration of C-11 was assigned as *R*, by applying Horeau’s method to the C-7 unsaturated and saturated diols, respectively, obtained after LAH reduction. This assignment was confirmed by ^1^H NMR analysis of the Mosher’s esters in a subsequent study by Fontana et al. [52] that isolated both compounds from *S. agaricina* from NE Spain. These authors also determine a *R* absolute stereochemistry for C-8 of **118** on the basis of nOe spectra. For furospongin-1 **119** the absolute configuration at C-11 was established as *S* by applying Horeau’s method. Subsequent studies by Kobayashi et al. [53] corrected this assignment to *R* by applying Mosher’s method, further supported by nOe studies and pyridine induced shift. Although the authors [49] assigned a *R* configuration to C-13 on the basis of chemical degradation of the dehydrated derivative, this was later corrected to *S* in a subsequent paper [50].

The same correction is applied to the configuration of C-13 of **123**. For **121** UV and IR indicated an α,β-unsaturated ketone, confirmed by ^1^H NMR with irradiation experiments of the signals of the corresponding H-12 and vinylic methyl. The low field resonance of the vinylic methyl at C-13 suggested it was *cis* to the carbonyl group. Further ^1^H NMR analysis led to the proposal of the structure. **122** showed UV, IR and mass spectra identical to **121**. The only difference in the ^1^H NMR spectrum was the upfield shift of the methyl at C-13 that led the authors to assume a different configuration of the ∆^12,13^ bond. For **123** the presence of the ketone was inferred from IR, and mass fragments corresponding to the cleavage of the C-10/C-11 bond, together with ^1^H NMR analysis, led to the proposal of the structure. For **124** the ketone group identified by IR, ^1^H NMR data analysis, the fact that it presented no optical rotation, and that in the mass spectrum only one fragment for α-cleavage of the carbonyl group was observed, led the authors to propose the *meso* compound depicted. **125** and **126** were isolated as a mixture resistant to separation. IR spectra indicated the presence of conjugated ester and carboxylic acid substituents that justify the intensity of the UV absorption observed, further confirmed by ^1^H NMR shifts of the corresponding olefinic protons. The location of the carboxylic acid was inferred from mass spectra and the *trans* orientation of the carboxyl substituents to the corresponding olefinic proton was established by spin decoupling experiments.

**Figure 31:**
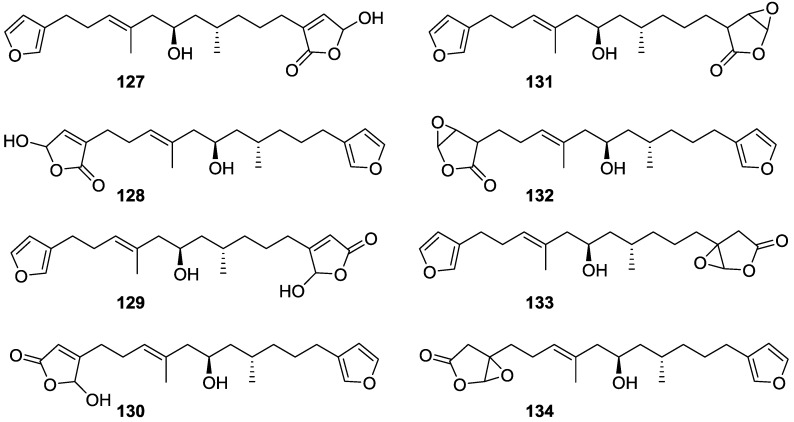
Structures of furospongin-1 **119** related compounds with γ-hydroxy-α,β-butenolide and β,γ-epoxybutenolide rings, **127**–**134**.

Further work by the same authors [54] on the same *S. officinalis* led to the isolation of eight new structures (isolated as mixtures) related to furospongin-1 **119**, with γ-hydroxy-α,β-butenolide and β,γ-epoxybutenolide rings, **127**–**134** (Figure 31).

The structures were identified as mixtures on the basis of UV, IR, ^1^H NMR data, mass spectra and comparison with furospongin-1 **119**. The mixtures of **127** and **128**, and **129** and **130** were also identified by chemical correlation with **119**. Since the structure of the latter was reviewed after the publication of this study, the structures here presented are also corrected. The mixture of **131**–**134** readily underwent decomposition to **127**–**130**. The fact that the authors were unable to identify any of the metabolites after exposure of a methanolic solution of furospongin-1 **119** to the light, reinforces the nature of the β,γ-epoxybutenolides as natural products.

**Figure 32:**
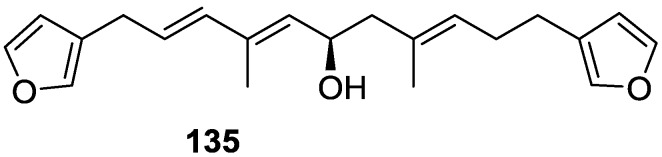
Structure of tetradehydrofurospongin-1 **135**.

Kazlauskas et al. [55] reported the isolation of tetradehydrofurospongin-1 **135** from an Australian *Spongia* sp (Figure 32). The proposed structure was subsequentially corrected.

**Figure 33:**
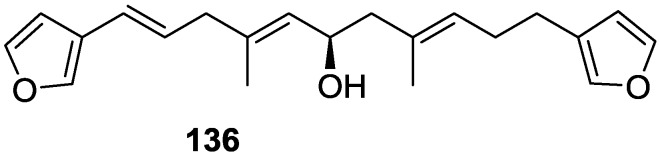
Structure of tetradehydrofurospongin-1 **136**.

The structure was identified on the basis of IR, ^1^H NMR data with double irradiation experiments, mass spectra and chemical transformation. Capon et al. [56] in a subsequent study in 1982 assigned ^13^C data, established the *E* configuration of the double bonds based on the ^13^C NMR resonances of the vinylic methyls and *J* coupling values of H-6 and H-7, and an *R* configuration at C-11 (Horeau’s method). In the already mentioned study of Fontana et al. [52] of 1996 the enantiomer of (−)-untenospongine B is isolated from *S. virgultosa* from NE Spain. The NMR data of this new compound **136** (Figure 33) was assigned by 1D and 2D experiments and an *R* configuration at C-11 was confirmed by ^1^H NMR analysis of the Mosher’s esters. Comparison of the obtained NMR data with that of the reported for **135** led the authors to reassign the structure of **136** to tetradehydrofurospongin-1.

**Figure 34:**
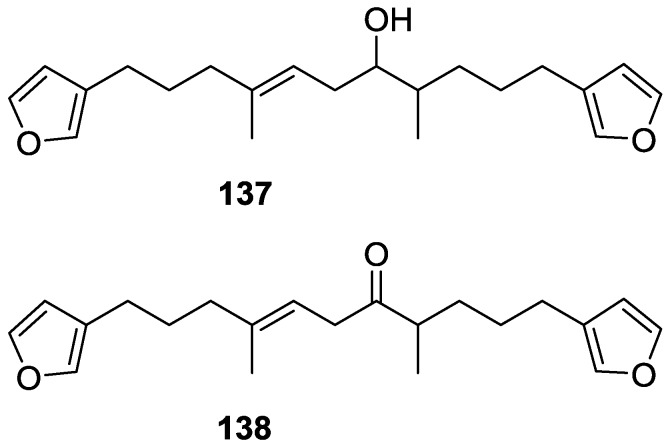
Structures of furospongenol **137** and furospongenone **138**.

Another study of Australian *Spongia* sp. by Kazlauskas et al. [57] led to the isolation of two new compounds, furospongenol **137** and furospongenone **138** (Figure 34).

The structures were identified on the basis of IR, UV, ^1^H NMR data with irradiation experiments, mass spectra and chemical transformation. No absolute configuration was assigned to **137**.

**Figure 35:**
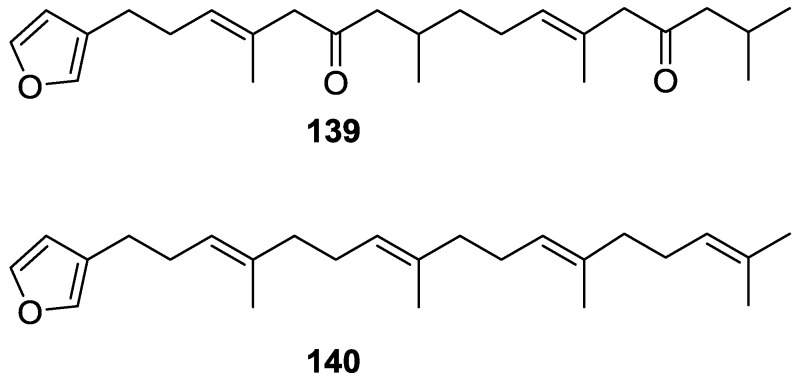
Structures of idiadione **139** and furospinulosin-1 **140**.

Walker et al. [58] reported the isolation of the new idiadione **139** and the known furospinulosin-1 **140** from *S. idia* de Laubenfels collected in San Diego, California (Figure 35).

**140** was identified by comparison with literature data. **139** was identified by IR, ^1^H and ^13^C NMR, and chemical transformation and degradation. The position of the ketone groups was established by mass fragmentation of the diketone-tetrahydrofuran obtained after full hydrogenation; NMR analysis of the products synthesized by reduction and acetylation, followed by ozonolysis and hydrogenation of the ozonides, confirmed the position of the double bonds; their geometry was determined by the chemical shifts of the vinylic methyls. **139** was toxic to the sea star *Pisaster giganteus* at a concentration of 5 mg/L, immobilized the larvae of the red abalone *Haliotis rufescens* at 1 mg/L in sea water, and was toxic to the ectoproct *Membranipora membranacea* at 10 mg/L. Both compounds were toxic to brine shrimp *Artemia* sp. at 10 mg/L.

**Figure 36:**
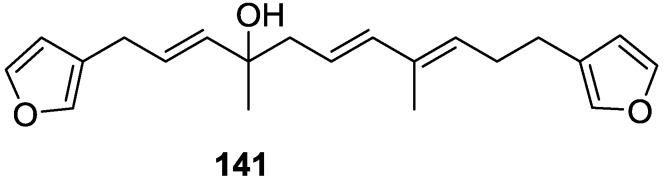
Structure of C-21 furanoterpene **141**.

From anWestern Australian *Spongia* sp., Capon et al. [56] isolated a new C-21 furanoterpene, **141** (Figure 36).

The structure was elucidated on the basis of UV, IR, ^1^H and ^13^C NMR, and mass spectra. The presence of a tertiary carbinol system came from IR, ^1^H and ^13^C NMR data. Significant downfield shifts were observed for the ∆^6,7^ vinylic protons and the methyl singlet upon recording the ^1^H NMR spectrum in the presence of tris[3-(trifluoromethylhydroxymethylene-d-camphorato]europium(III). Confirmation of the proposed structure came from ozonolysis. The *E* configuration of the double bond at C-11 came from *J*_H,H_ coupling analysis of H-11 and H-12; the same configuration was assigned to the double bond at C-13 on the basis of the high field resonance observed for Me-14 (shielded by *cis*-allylic methylene group). Analysis of the *J*_H,H_ coupling of H-6 and H-7 obtained by spectral simulation allowed the assignment of the *E* configuration to the ∆^6,7^ double bond.

**Figure 37:**
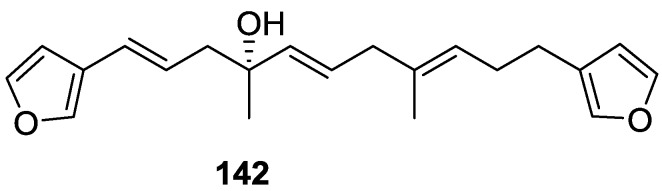
Structure of (−)-isotetradehydrofurospongin-1 **142**.

Subsequent work of Capon et al. [59] on *Spirastrella papilosa* led to the reisolation of the compound, and revision of the proposed structure to **142** (Figure 37), for which the name of (−)-isotetradehydrofurospongin-1 is proposed.

The revision was based on 2D NMR data. The *E* configuration of ∆^5,6^ was confirmed by *J* coupling analysis and the chemical shift of Me-14 in ^13^C NMR; the *E* configuration of ∆^10,11^ is confirmed by NOESY. Ozonolysis was repeated and (*R*)-dimethyl citramalate was recovered: its assignment was confirmed by ^1^H NMR, [α]_D_ and chiral HPLC comparison with authentic samples of both the *R* and *S* enantiomers.

**Figure 38:**
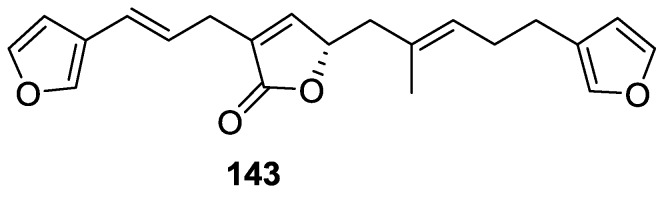
Structure of kurospongin **143**.

Tanaka and Higa [60] reported the isolation of the new kurospongin **143** from a *Spongia* sp. collected in Miyako Island, Japan (Figure 38).

The structure was identified by mass spectrometry, IR, ^1^H and ^13^C NMR and irradiation experiments. These allowed the identification of the furan rings, the α,β-unsaturated γ-lactone, a vinyl methyl, a *trans* di-substituted double bond and a tri-substituted double bond. The geometry of the latter was assigned as *E* by the value of the ^13^C chemical shift of the vinylic methyl. The absolute stereochemistry at C-11 was assigned as *S* by applying Horeau’s partial resolution method to the diol obtained after treatment with ethylmagnesiumbromide. Compound **143** was ichthyotoxic, killing goldfish at the concentration of 5 μg/mL within 4 h. In feeding experiments using the omnivorous fish *Tilapia mosambica* **143** impregnated in feed completely deterred its consumption at the concentration level of 0.3%.

**Figure 39:**
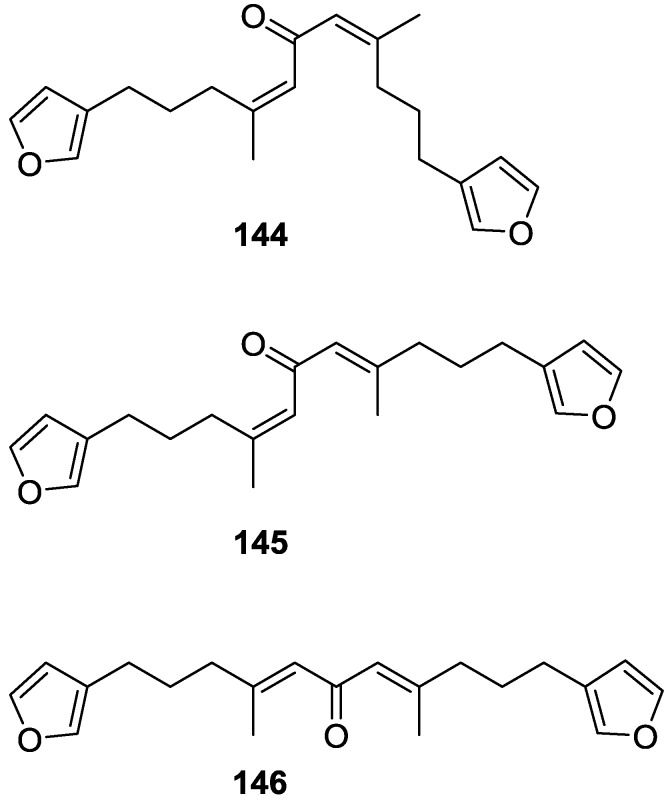
Structures of furospongin-2 **121** isomers **144**–**146**.

De Giulio et al. [61] reported the isolation of furospongin-2 **121**, together with its three new isomers **144**–**146** (Figure 39), from a *S. officinalis* L. collected in northern Adriatic, whose extract showed cytotoxic activity (LD50 45 μg/mL) in the brine shrimp assay.

For all compounds, the presence of an α,β-unsaturated ketone was established by UV and IR and confirmed by ^1^H and ^13^C NMR spectra. The use of COSY and HETCOR spectra allowed the assignment of all resonances for **144**. For this compound the fact that the ^13^C NMR spectra only showed 11 signals led to the conclusion that it was symmetrical. Comparison of its data with the remaining compounds led to identification of the latter. The stereochemistry of the double bonds of all compounds were assigned on the basis of the chemical shifts in ^1^H and ^13^C spectra for the vinylic methyls and allylic methylenes. ^13^C NMR data for **121** is assigned based on COSY and HETCOR. All compounds showed high activity (LD50 0.09–1.6 μg/mL) in the *Artemia salina* shrimp bioassay, an in-house substitute for 9 KB and 9 PS cytotoxicities.

**Figure 40:**
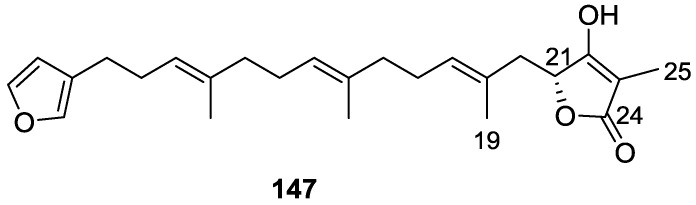
Structure of tetronic acid **147**.

Lumsdon et al. [62] reported the isolation of the new tetronic acid **147** from a *Spongia* sp. collected in Australia (Figure 40). The crude ethanol extract evoked a large triphasic contraction of smooth muscle in the isolated guinea-pig ileum. It also appeared to inhibit contractions elicited by different drugs (acetylcholine, 5-hydroxytryptamine and histamine) of the isolated guinea-pig ileum, and inhibited the growth of several bacteria (*Staphylococcus* *aureus*, *Micrococcus* sp. and *Serrata* sp.) in a standard antibiotic disk assay.

Compound **147** was identified by NMR, where resonances for a β-substituted furan, three substituted double bonds with vinylic methyls and a tetronic acid moiety were observed. The presence of this latter feature and confirmation of the structure came by comparison with palominin, its known geometrical isomer. The observed chemical shifts for the olefinic methyl resonances of **147** confirmed the *E* geometry of all double bonds. CD data supported a 21*R* stereochemistry. The antibiotic activity of the extract was attributed entirely to **147**. Preliminary testing suggested that this compound was also responsible for the inhibitory activity detected in the crude ethanol extract. This compound reversibly blocked contractions, evoked by acetylcholine, 5-hydroxytryptamine and histamine, of isolated guinea-pig ileum, and electrical stimulation of intrinsic nerves. Purification of the extract appeared to remove the contracting substance detected, due to its loss or to a synergistic activity between the isolated compounds.

**Figure 41:**
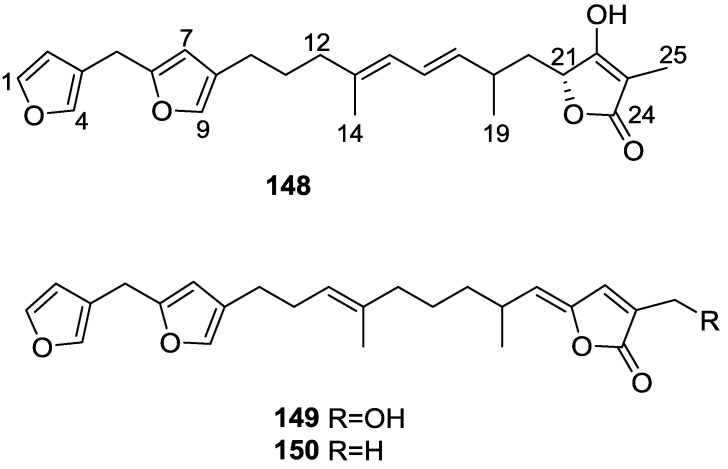
Structures of cometins A–C **148**–**150**.

Urban and Capon [63] reported the isolation of the new cometins A–C **148**–**150** (Figure 41), together with the known furospinosulin-1 **140**, from a *Spongia* sp. collected in the Great Australian Bight.

Compound **148** was identified by mass spectrometry and ^1^H and ^13^C NMR where resonances for the difuran and tetronic acid moieties were identified. Confirmation came from comparison with literature compounds. The 1,1,4-trisubstituted 1,3 diene functionality was further identified by NMR. The geometry of the ∆^16,17^ double bond was determined as *E* by the *J* coupling value observed for the olefinic protons. Comparison of the δ value in ^13^C NMR for the olefinic methyl with reference compounds allowed the determination of the *E* configuration for the trisubstituted double bond. Although stereochemistry at C-18 was not determined, a *R* configuration is proposed on the basis of CD data. Comparison of the NMR data of **149** with that of **148** allowed the replacement of the tetronic acid moiety by a conjugated γ-butenolide. Confirmation of the structure came from COSY spectra and nOe. The geometry of the double bond was determined as *E* by the ^13^C NMR shift of the olefinic methyl. Stereochemistry at C-18 was not determined. Comparison of the data of **150** with that of **149**, together with analysis of the COSY spectra, allowed the proposal of the structure for the former. The geometry of the ∆^12,13^ double bond was determined as *E* by the ^13^C chemical shift of Me-14, and the presence and orientation of the butenolide fragment were confirmed by MS (observation of the fragment derived by allylic fragmentation) and nOe (enhancement of H-20 and Me-25 upon irradiation of H-22). Biological testing of the pure compounds against *Staphylococcus aureus* and a *Serratia* sp. confirmed that only **148** was active. The minimum concentrations for these activities were determined as 3–5 μg/disk and 5 μg/disk, respectively.

**Figure 42:**
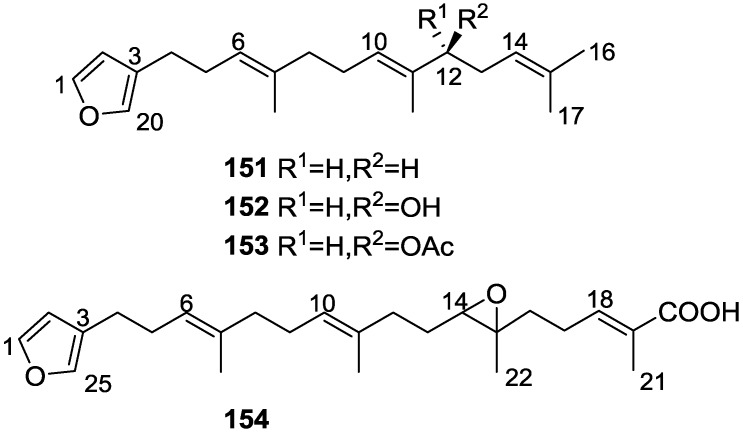
Structures of ambliofuran **151**, (*S*)-12-hydroxyambliofuran **152**, (*S*)-12-acetoxyambliofuran **153**, and compound **154**.

From the already mentioned study of Searle et al. [30] of a *Spongia* sp. from Western Australia, the known ambliofuran **151** together with the new (*S*)-12-hydroxyambliofuran **152**, (*S*)-12-acetoxyambliofuran **153**, and **154** were identified (Figure 42).

Compound **151** was identified by comparison of ^1^H and ^13^C NMR data with the literature. For compound **153** the location of the acetoxy substituent (confirmed by IR and ^1^H signal of the corresponding methine) was determined by COSY, HETCOR and HMBC data. Compound **152** was assigned by comparison with **153**. Hydrolysis of the latter allowed confirmation of the proposed structure. The absolute configuration of **152** and **153** (after hydrolysis) was determined by a modified Mosher’s method. For both compounds a 3:1 mixture of enantiomers was determined, with excess of the 12*S* enantiomer. This ratio was confirmed by the ^1^H NMR spectra of **153** in the presence of Eu((+)-hfc)_3_. The authors suggest the existence of two different enzymes, one with the *S* specificity and other with the *R* that would oxidize **151** to **152**; further acetylation would produce **153** in the same ratio. For **154**, the presence of a trisubstituted epoxide was suggested by ^13^C NMR. COSY, HETCOR, COLOC and 2D-INADEQUATE spectra allowed confirmation of the proposed structure. Although the absolute stereochemistry of **154** was not determined, a *trans* epoxide is proposed on the basis of high field signal of the attached methyl group in ^13^C NMR (due to steric compression by the *syn* methylene group) and comparison with the observed values for the corresponding methyl group in *trans* geraniol-2,3-epoxide and *cis* nerol-2,3-epoxide. The *E* configuration of the double bonds was determined by the ^13^C chemical shift values of the vinylic methyls.

**Figure 43:**
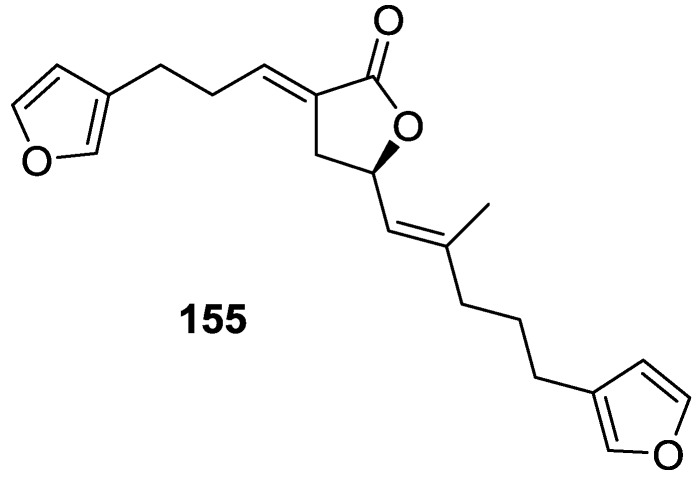
Structure of isonitenin **155**.

Lenis et al. [64] reported the isolation of the new isonitenin **155** (Figure 43) and the known nitenin **117** and dihydronitenin **118** from *S. officinalis* collected in the Galician coast.

The known compounds were identified by comparison with literature data. The structure of **155** was established by NMR (including ^1^H–^1^H COSY and HMQC). Comparison of the data with that of **117** showed an downfield shift of H-7 and upfield shift of CH_2_-6, consistent with an *E* stereochemistry of the ∆^7,8^ double bond. This geometry was further confirmed by the upfield shift observed for C-10 due to the *cis* arrangement of C-10 and C-6. Since optical rotation and CD spectrum of **155** where almost identical to those of **117** an *R* stereochemistry at C-11 is proposed. For **118** an 8*R* stereochemistry is proposed on the basis of nOe observed between H-8 and H-11. The *E* stereochemistry of the ∆^12,13^ double bond was confirmed by nOe between CH_2_-15 and H-12.

**Figure 44:**
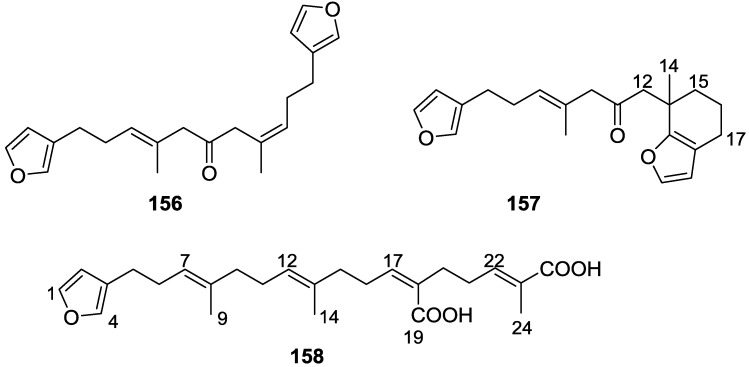
Structures of furospongin-5 **156**, cyclofurospongin-2 **157**, and demethylfurospongin-4 **158**.

Garrido et al. [65] reported the isolation of the new furospongin-5 **156**, cyclofurospongin-2 **157** and demethylfurospongin-4 **158** (Figure 44), together with the known, **121**–**124**, **126**, and **144**–**146**, from a *S. officinalis* L. collected in Cádiz, Spain.

Compound **156** was identified by comparison with **121** and analysis of the IR (non conjugated ketone) and NMR data. The stereochemistry of the double bonds was determined on the basis of the ^13^C chemical shifts of the vinylic methyls. Confirmation of the proposed structure and double bond stereochemistry came from nOe difference spectroscopy, where irradiation of H-7 caused enhancement of CH_2_-10, and irradiation of H-15 enhanced Me-14. For **157** IR, ^1^H and ^13^C data showed it was an isomer of **156** with a (*E*)-furylmethylpentenyl fragment linked to a central ketone. An extra α,β-disubstituted furan ring with a fused methylated cyclohexene and an isolated methylene were established by ^1^H and ^13^C NMR. Acid treatment of **121** yielded (±)-cyclofurospongine-2 as expected. Since the natural product is optically active occurrence of the cyclisation process during isolation is excluded. The structure of **158** was established by IR, ^1^H and ^13^C NMR data, and comparison with the data of a mixture of **125** and **126,** and **154**. ^13^C NMR data is presented for all compounds. The new compounds were tested against P-388, A-549, HT-29 and MEL-28. Compounds **156**–**158** showed low cytotoxicity with E97 values over 10 μg/mL in all cases with the exception of **156** that showed mild cytotoxicity against P-388 cell line (ED50 5 μg/mL).

**Figure 45:**
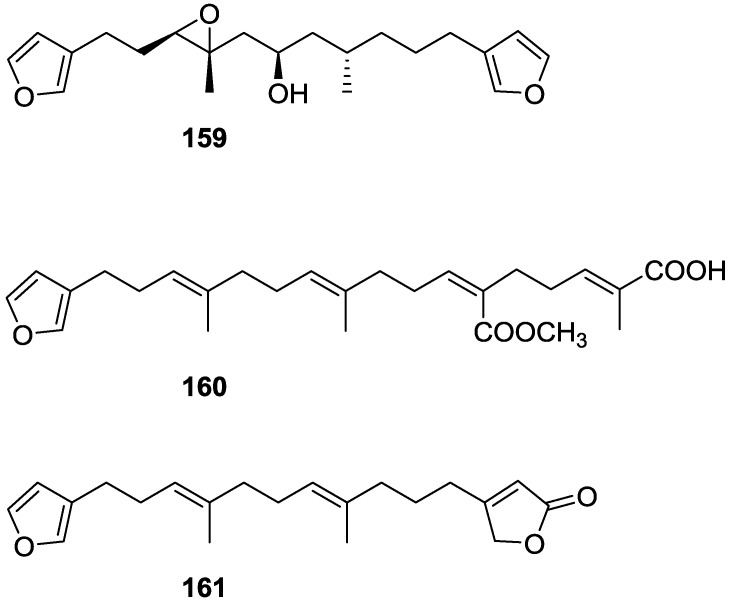
Structures of 7,8-epoxy-furospongin-1 **159**, isofurospongin-4 **160,** and compound **161**.

Manzo et al. [66] reported the isolation of the new 7,8-epoxy-furospongin-1 **159** and isofurospongin-4 **160** together with the known **119**, **120**, **123**, **124**, **126** and **161**, from *S. officinalis* L. collected in Sicily (Figure 45).

Compound **159** was identified by NMR data and comparison to furospongin-1 **119**. The presence of an epoxide ring was identified by ^1^H and ^13^C NMR and the proposed structure was confirmed by 2D NMR. The relative configuration was determined by nOe difference experiments where effects where observed between CH_2_-6 and Me-9, and between H-7 and CH_2_-10. The absolute configuration at C-11 was established by applying a modified Mosher’s method. Based on biogenetic considerations the absolute configuration at C-13 was assigned as in furospongin-1 **119**. The NMR data of **160** closely resembled that of furospongin-4 **126**. Analysis of ^1^H–^1^H COSY, HSQC and HMBC spectra showed that both compounds differed in the esterification site. Further confirmation of the structure came from comparison of the dimethyl ester obtained from both compounds. Compounds **119**, **120**, **123**, **124**, **126**, **160** and **161** were tested for antibacterial and antifungal activity against *E. coli*, *Staphylococcus aureus* and *Candida albicans*. Only **126** showed weak activity against *S. aureus* at 100 μg/mL. For compounds **123** and **124** an interesting biofilm induction activity of *E. coli* PHL628 was observed, this activity being more efficient by an increase in the concentration of **124**. The authors suggest that this activity is related to the symbiosis that marine organisms are able to form with strains of bacteria that do not allow biofouling stratification on their surfaces.

### Other Studies

**Figure 46:**
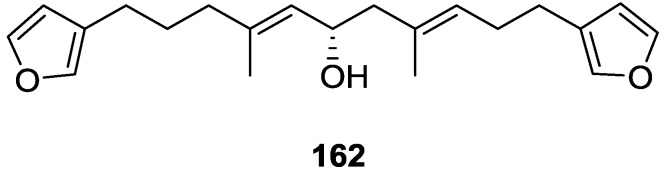
Structure of compound **162.**

Li et al. [32] reported the isolation of the known **162** (Figure 46) from the already mentioned study of on a *S. matamata* de Laubenfels collected in Yap Island, Micronesia, a specimen reclassified as *S. zimocca sensu* de Laubenfels by the same authors in a subsequent study [33].

Purified **162** showed mild toxicity, with LC_50_ values of approximately 50–100 μg/mL, in the brine shrimp lethality test.

Rueda et al. [67] reported the isolation of the known **117**, **118**, **120**, **140**, and **155** from *S. agaricina* collected in Cádiz, Spain.

## 5. Sesterterpenes

**Figure 47:**
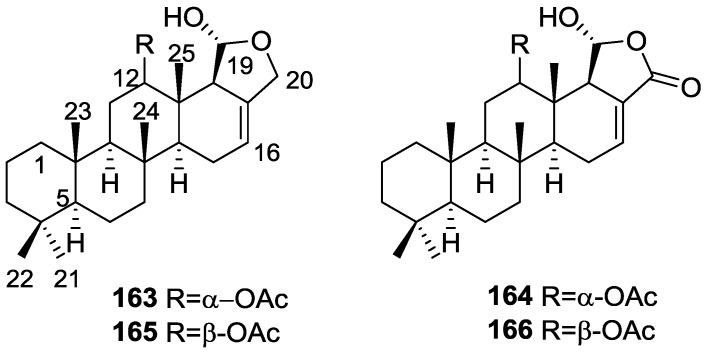
Structures of deoxoscalarin **163**, scalarin **164**, 12-*epi*-deoxoscalarin **165**, and 12-*epi*-scalarin **166**.

Cimino et al. [68] reported the isolation of deoxoscalarin **163** from *S. officinalis*, collected in Naples. The C-19 stereochemistry of this structure was revised in a subsequent publication [69] where the known scalarin **164** was isolated from *S. virgultosa* and the new 12-*epi*-deoxoscalarin **165** and 12-*epi*-scalarin **166** were isolated from the Mediterranean *S. nitens*, whose extracts had already afforded furanoterpenes [8] (Figure 47).

Compound **163** was identified by ^1^H NMR with spin decoupling, mass spectrometry, comparison with **164** and chemical correlation between both compounds. For all compounds the mass fragments corresponding to the rupture of ring D at *m/z* 258 (*retro* Diels Alder and concomitant loss of acetic acid), ring C at *m/z* 205 and *m/z* 191 (cleavages of the 8,14 and 11,12, and 8,14 and 9,11 bonds, respectively, with associated loss of hydrogen from the charge retaining rings A and B) and fragmentation of ring B at *m/z* 137 and *m/z* 123 (cleavage of the 6,7 and 9,10, and 5,6 and 9,10 bonds, respectively) are identified. Comparison of the ^1^H NMR data showed main differences in the shape of the H-12 signal and *J* analysis established its orientation. Further evidence came from the fact that scalarin type compounds (12-OH axial) are more readily oxidized by Jones’ reagent than *epi*-scalarin compounds (12-OH equatorial). Chemical correlation of **165** and **166** with **164** confirmed their structures. Analysis of several derivatives (NMR, CD and shifts induce by Eu([^2^H_9_]fod)_3_) allowed the determination of a *trans*-*transoid*-*trans*-skeleton with an H-18-α absolute configuration and an axial orientation of the Me-24, Me-25, H-9 and H-14. Stereochemistry at C-19 was again inferred from chemical studies of derivatives. ^13^C NMR data is presented for **164**, **165** and **166**.

**Figure 48:**
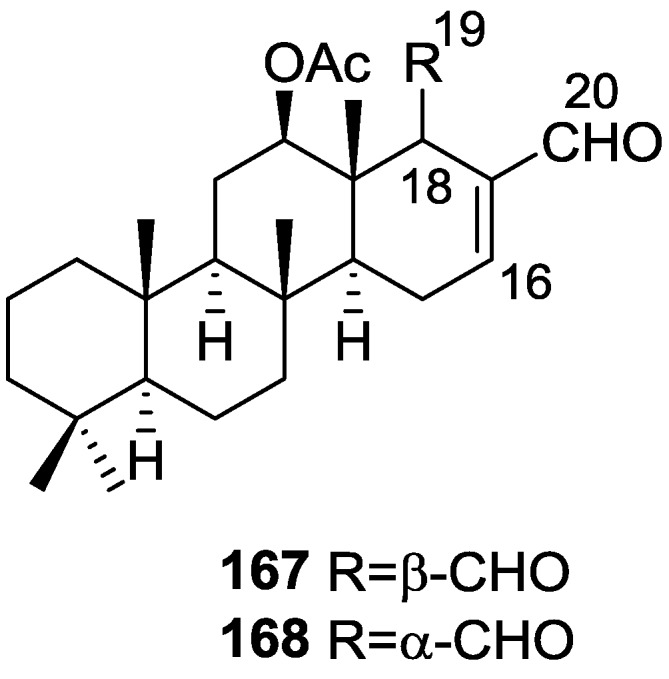
Structures of 12-*epi*-scalaradial **167** and 12,18-di*-epi*-scalaradial **168**.

Further studies on the extracts of the same *S. nitens* [70] led to the isolation of 12-*epi*-scalaradial **167** and 12,18-di*-epi*-scalaradial **168** (Figure 48).

Compound **167** was assigned on basis of the UV, IR, ^1^H and ^13^C NMR data and comparison with the known scalaradial. Orientation at C-12 was based on the shape of the H-12 signal in ^1^H NMR. Full confirmation, including absolute stereochemistry, came from correlation with 12-*epi*-deoxoscalarin **165**. ^13^C NMR data comparison of **167** with that of scalaradial allowed the confirmation of its structure and determination of an all *trans*-*anti*-*trans* configuration. Compound **168** was established by comparison of the obtained data with that of **167**. ^13^C NMR data allowed confirmation of its structure and determination of the stereochemistry.

**Figure 49:**
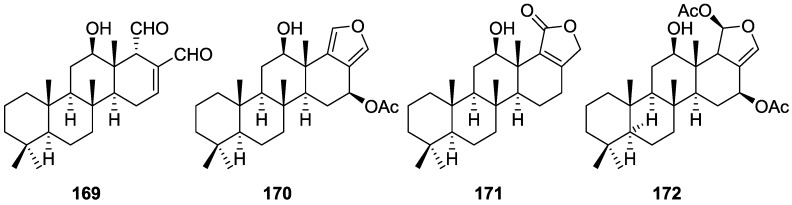
Structures of 12-deacetyl-12,18-di-*epi*-scalaradial **169**, scalarafuran **170**, scalarolide **171**, and **172**.

From the already mentioned study of Walker et al. [58] 12-deacetyl-12,18-di-*epi*-scalaradial **169**, scalarafuran **170** and scalarolide **171**, together with the known heteronemin **172** and **165**, were isolated from *S. idia* de Laubenfels, collected in San Diego, California (Figure 49).

Compounds **165** and **172** were identified by comparison with literature values. **169** was converted into the known 12,18-di-*epi*-scalaradial **168**. Compound **170** was obtained from **172** by controlled pyrolysis. For **171** a scalarin skeleton with an equatorial hydroxyl at C-12, an olefin at C-17(18), and lactone were inferred from ^1^H and ^13^C data. The position of the lactone at C-19 was established by chemical transformation and confirmed by chemical correlation to **172**. Compound **169** was toxic to the sea star *Pisaster giganteus* at a concentration of 5 mg/L, immobilized the larvae of the red abalone *Haliotis rufescens* at 1 mg/L in seawater and was toxic to the hydroid *Bougainvilla* sp. at 10 mg/L. Compounds **165**, **169** and **172** were toxic to brine shrimp *Artemia* sp. at 10 mg/L. Compound **172** immobilized the larvae of the red abalone *Haliotis rufescens* at 1 mg/L in seawater and was toxic to the gametes of the giant kelp *Macrocystis pyrifera* at 10 mg/L.

**Figure 50:**
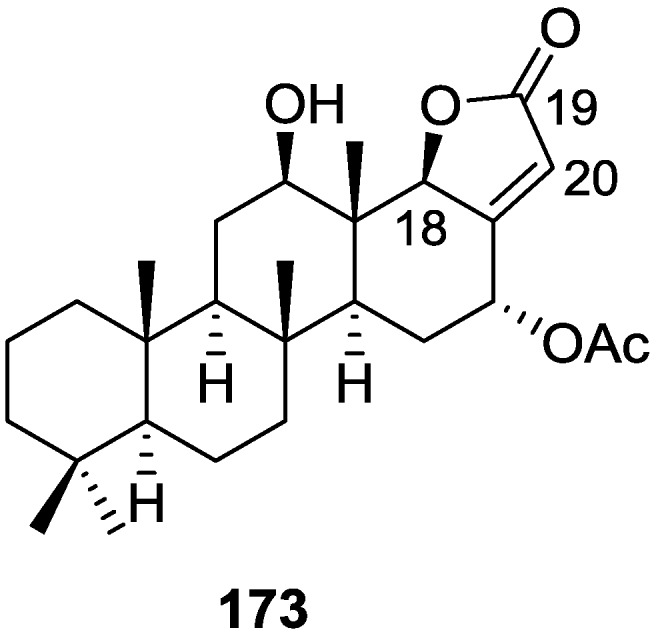
Structure of scalarolbutenolide **173**.

Subsequent work by Cimino et al. [71] on *S. nitens* allowed the isolation of scalarolbutenolide **173** (Figure 50).

A tetracyclic scalarin type skeleton with an acetoxy group and a hydroxyl at C-12 was inferred from mass spectra. This latter assignment is confirmed by the corresponding H-12 signal in ^1^H NMR. Comparison of ^13^C data (including decoupling experiments) with those of known compounds confirmed the proposed structure. The upfield shifts observed upon acetylation indicated the presence of a hydrogen bond between C-12-OH and the lactone oxygen. The localization of the acetoxy group in C-16 was suggested by the ease of elimination observed in mass spectra. Its orientation was based on the upfield shift of H-14 when compared with a known compound, justifiable by a γ-gauche interaction with an axially oriented group. The upfield resonance of C-25 indicated an α-orientation of C-18. The β,γ-disubstituted-α,β-butenolide ring suggested by UV, IR and ^1^H NMR data was confirmed by chemical transformation. The relative stereochemistry is in agreement to previous findings for scalarin type compounds. Application of Horeau’s method determined an *R* absolute configuration at C-12.

**Figure 51:**
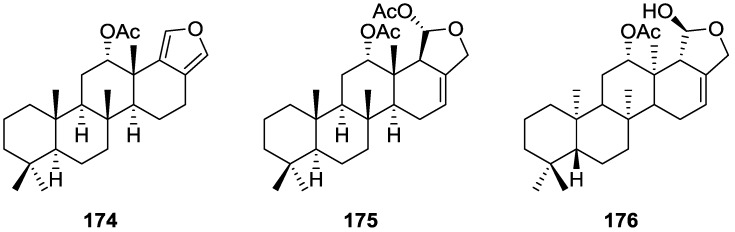
Structures of 16-deacetoxy-12-*epi*-scalarafuran acetate **174**, deoxoscalarin acetate **175**, and (−)-12-*epi*-deoxoscalarin **176**.

From the already mentioned study of De Giulio et al. [61] 16-deacetoxy-12-*epi*-scalarafuran acetate **174** and deoxoscalarin acetate **175**, together with (−)-12-*epi*-deoxoscalarin **176** (Figure 51), were isolated from a *S. officinalis* L. collected in northern Adriatic, whose extract showed cytotoxic activity (LD50 45 μg/mL) in the brine shrimp assay.

For all compounds ^1^H and ^13^C NMR data comparison with literature compounds supported a scalarane skeleton. For **174** the presence of an acetyl group was inferred from IR data and confirmed by ^1^H NMR. This spectra also showed the typical signals of a β,β-disubstituted furano ring. This data suggested that **174** was identical to the furan obtained in the reaction of acetylation of deoxoscalarin **163** [68]. The relative stereochemistry at C-12 was assigned by the chemical shift and multiplicity observed in ^1^H NMR. Compound **175** was identified by IR and ^1^H NMR data that showed it to be the acetyl derivative of deoxoscalarin **163**. **175** was unstable and readily transformed into **174**, what suggested that **174** was an artifact. The spectral data of **176** (including COSY and NOESY) were in agreement with those of **165**. The fact that it presented a similar melting point and an optical rotation of the same magnitude but opposite sign indicated it was the enantiomer of **165**. All compounds were tested in the *Artemia salina* shrimp bioassay, an in-house substitute for 9 KB and 9 PS cytotoxicities, (LD50 180–200 mg/mL). The authors point out that the co-occurrence of two related compounds (**175** and **176**) as epimers at C-12 shows that the enzymatic pathways for oxidation at C-12 for scalarane sesterterpenes are non stereoselective.

**Figure 52:**
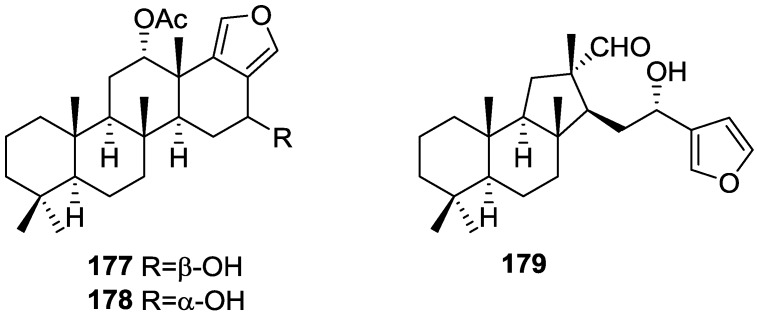
Structures of isoscalarafuran A **177**, isoscalarafuran B **178**, and hyrtiosal **179**.

Davis and Capon [72] reported the isolation of the new isoscalarafuran A **177** and isoscalarafuran B **178**, together with the known hyrtiosal **179**, from a *S. hispida* collected in the Great Australian Bight (Figure 52).

Compound **179** was identified by comparison with literature data. For **177** NMR data identified five tertiary methyls, two furan α-protons, acetoxy and hydroxy functions, and five sp^2^ carbons that led the authors to propose a tetracyclic sesterterpene. Comparison of the data with the known scalarafuran **170** and other literature compounds confirmed the localization of the hydroxy and acetoxy functions. Confirmation of the α-orientation at C-12 was obtained by the ^1^H chemical shift and multiplicity of H-12. Stereochemistry at C-16 was determined by molecular modelling. For **178** the main difference in respect to the ^1^H NMR data of **177** was the H-16 resonance, consistent with **178** being the C-16 epimer. Both compounds underwent decomposition before absolute stereochemistry, optical rotation and biological activity could be determined.

**Figure 53:**
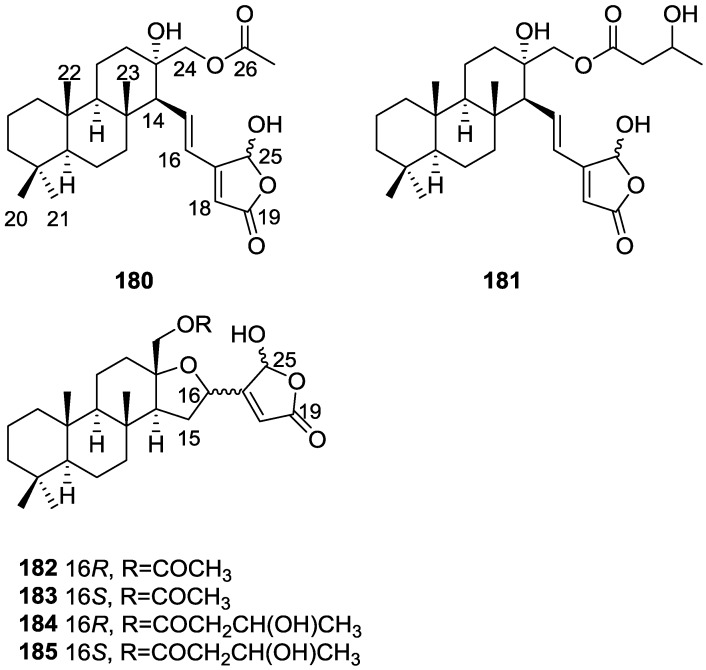
Structures of spongianolides A–F **180**–**185**.

He et al. [73] reported the isolation of the new spongianolides A–F **180**–**185** (Figure 53) from a *Spongia* sp. collected in Florida in a bioguided study of protein kinase C (PKC) inhibitors.

For **180** the sesterterpenoid skeleton was suggested by four methyl resonances in ^1^H and ^13^C NMR spectra where additional resonances could be assigned to rings A and B of spongiane type diterpenes. A γ-hydroxide butenolide moiety was also identified by NMR and UV indicated conjugation with a *trans* double bond. The full structure was assigned by HMBC. The A/B and B/C ring *trans* junctions were supported by nOe (H-9 to H-5, and H-9 to H-14). The β-configuration of the substituents at C-13 and C-14 was based on nOe between Me-23 and one CH_2_-24, and Me-23 and H-15. For **181,** comparison with **180** showed the replacement of the acetyl group by a γ-hydroxybutyryl group. Comparison of **182** and **183** with **180** revealed that the olefinic protons were replaced by a methylene and an oxygenated methine, consistent with a tetrahydrofuranyl ring confirmed by COSY spectra. The authors suggest that biosynthetically this ring is formed by addition of C-13-OH to C-16 in **180**. The diastereomeric relationship of **182** and **183** at C-16 was established by the *J* coupling values of H-16 with the diastereotopic H-15. The configuration of this center in both compounds was determined by nOe (H-18/H-24 for **182** and H-16/H-24, H-16/H-15β and Me-23/H-24 for **183**). The measured coupling constant of CH_2_-15 and H-16 were very close to the corresponding values obtained from molecular mechanics calculations. Compounds **184** and **185** possessed a γ-hydroxybutyryl ester side chain instead of the acetyl group of **182** and **183**. Lactones **180**–**184** inhibited PKC at IC_50_ 20–30 μM and did not inhibit the human 85 kD phospholipase A_2_. Compounds **180**–**183** potently inhibited (IC_50_ 0.5–1.4 μM) the proliferation of the mammary tumor cell line MCF-7. After completion of the manuscript compounds **182** and **183** were isolated from another sponge and designated as lintenolides A and B [74]. However, they were characterized only after acetylation and only partial spectroscopic data were reported for the natural products.

**Figure 54:**
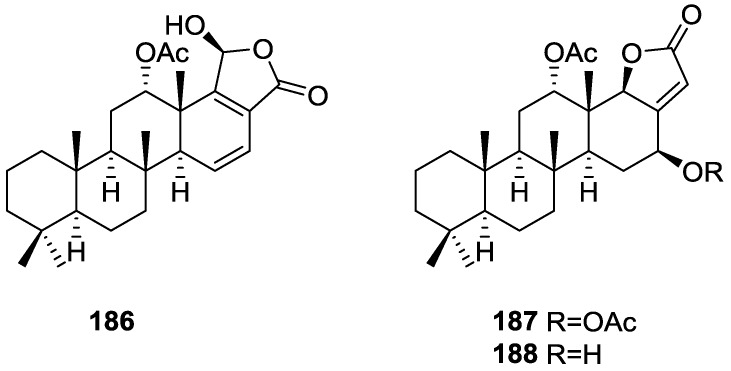
Structures of 12α-acetoxy-19β-hydroxyscalara-15,17-dien-20,19-olide **186**, 12α,16β-diacetoxyscalarolbutenolide **187**, and 12α-acetoxy-16β-hydroxyscalarolbutenolide **188**.

Lu and Faulkner [75] reported the isolation of 12α-acetoxy-19β-hydroxyscalara-15,17-dien-20,19-olide **186** and 12α,16β-diacetoxyscalarolbutenolide **187**, together with the known scalarin **164** and 12α-acetoxy-16β-hydroxyscalarolbutenolide **188**, from *S. matamata* de Laubenfels 1954, collected in a marine lake at Palau, Western Caroline Islands (Figure 54).

For **186** the presence of five methyl signals and an acetate group in ^1^H NMR indicated a pentacyclic scalarane skeleton. UV (conjugated diene), IR, ^1^H and ^13^C NMR, HMQC, HMBC, DQCOSY and NOEDS spectra allowed identification: coupling of the olefinic protons to a methine placed the conjugated diene on ring D; H-12 was assigned by HMBC correlations with Me-25 and nOe showed that it is β-equatorial (irradiation produced nOe on H-19 and Me-25); nOe also defined the regiochemistry of the butenolide ring showing that H-19 (which overlapped with H-15) is α-oriented (irradiation caused enhancement on the acetate methyl signal). **187** showed spectral data very similar to **188**, except for the presence of an extra acetyl group. The significant downfield shift of H-16 confirmed its location. Further confirmation came from acetylation of **188**. This compound had been reported in the abstract of a paper but not on the main text [76]. Its full characterization is reported and its assignment is based on spectral data analysis and comparison to 16-*O*-deacetyl-16-*epi*-scalarolbutenolide. The stereochemistry at C-12 was deduced from the small coupling constants observed.

**Figure 55:**
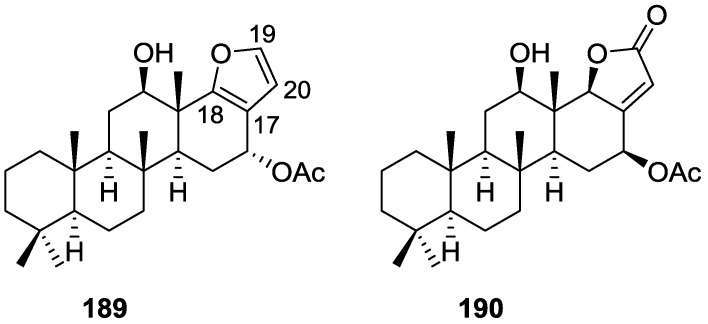
Structures of 12,16-di-*epi*-12-*O*-deacetyl-16-*O*-acetylfuroscalarol **189** and 16-*epi*-scalarolbutenolide **190**.

From the already mentioned study of Rueda et al. [67] on *S. agaricina*, from Cádiz, Spain, 12,16-di-*epi*-12-*O*-deacetyl-16-*O*-acetylfuroscalarol **189** and 16-*epi*-scalarolbutenolide **190** (Figure 55), together with the known 12-*epi*-scalaradial **167**, 12,18-di-*epi*-scalaradial **168**, 12-*epi*-deoxoscalarin **165**, 12-*epi*-scalarin **166**, and scalarolbutenolide **173** were isolated.

For **189** the signals in ^1^H NMR for the five methyl groups and the furan moiety established the furoscalarol skeleton. IR and ^1^H and ^13^C NMR identified the hydroxyl and acetyl groups. Comparison with the known furoscalarol indicated substantial differences on the signals of protons geminal to the oxygenated functionalities. Thus, H-12 was assigned as axial and H-16 as equatorial. **190** was assigned a scalarane type skeleton on the basis of the ^1^H and ^13^C NMR signals of the five methyl groups. IR and ^13^C NMR established the α,β-unsaturated-γ-lactone. Comparison with the data of scalarolbutenolide **173** indicated the same functionality and stereochemistry at C-12 and an opposite orientation of the acetoxy group at C-16. Compound **189** showed cytotoxicity against P-388, A-549, HT-29 and MEL-28 tumor cell lines with IC_50_ values of 1 μg/mL and **190** showed weaker activity with IC_50_ of 5 μg/mL.

**Figure 56:**
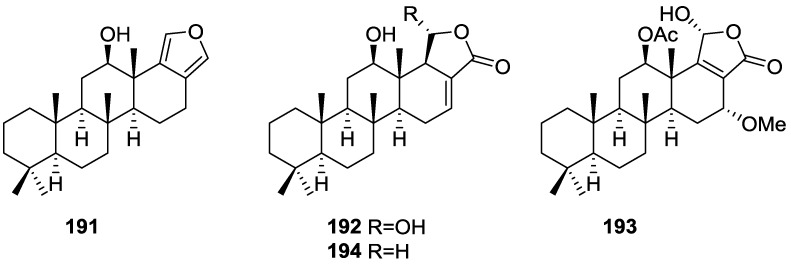
Structures of 12-*O*-deacetylscalafuran **191**, 12-*O*-deacetyl-12-*epi*-scalarin **192**, 12-*O*-acetyl-16-*O*-methylhyrtiolide **193**, and 12-*O*-deacetyl-12-*epi*-19-deoxyscalarin **194**.

Tsukamoto et al. [77] described the bioassay guided isolation of 12-*O*-deacetylscalafuran **191**, 12-*O*-deacetyl-12-*epi*-scalarin **192** and 12-*O*-acetyl-16-*O*-methylhyrtiolide **193** (Figure 56) together with the known 12-*O*-deacetyl-12-*epi*-19-deoxyscalarin **194**, 12-*epi*-deoxoscalarin **165** and 12-*epi*-scalarin **166**, from a *Spongia* sp. collected in the sea of Japan.

Compound **191** was assigned on the basis of ^1^H and ^13^C NMR, HMQC and HMBC data. The pentacyclic sesterterpene skeleton was inferred from the ^13^C NMR resonances of the five methyl groups and the presence of a furan ring was established by HMBC. The C-12-OH was suggested by IR and H-12 was established as α-axial on the basis of *J* couplings. Although this compound had previously been reported as a product of the hydrogenolysis of scalarafuran **170**, this was its first occurrence as a natural product. Compound **192** was assigned by NMR data. The C-12-OH was established as α-axial on the basis of *J* couplings of H-12. nOe correlation with H-18 implied that the latter was also α-oriented. Stereochemistry at C-19 could not be resolved. Further confirmation came from the transformation into 12-*epi*-scalarin **166** by acetylation. For **193** the γ-hydroxy-butenolide ring was suggested by the deshielded methine signals, two quaternary olefinic carbons and a carbonyl resonance. Comparison of the data with that of hyrtiolide showed the extra *O*-acetyl and *O*-methyl groups, whose location came from HMBC. *J* couplings showed that H-12 was α-axial and that H-16 was β-equatorial. nOe showed that H-19 was β-oriented (correlation with Me-25). All six compounds were tested against L1210, HeLa, A549 and KB cell lines. The results are in Table 2.

Compounds **192**, **165** and **194** were also tested for in vivo mean survival times (MST) and increases of life spans (ILS) in sarcoma-180-implanted mice. **194** showed significant ILS: 50.3% of ILS at 5 mg/Kg intraperitonial administration, and this is more potent than a positive control 5-fluoroacil (5-FU; 32.9%) at the same dose. Compound **165** also showed comparable ILS (28%) to 5-FU at 10 mg/kg, and **192** was inactive at 5 mg/kg.

**Figure 57:**
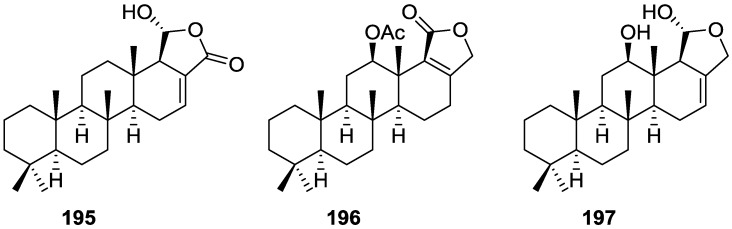
Structures of deacetoxy scalarin **195**, and compounds **196** and **197**.

Tokue et al. [78] reported the isolation of the new deacetoxy scalarin **195**, together with the known **166**, **192**, **194**, **196** and **197** from a *Spongia* sp. collected in Toyama Bay, Japan sea (Figure 57).

The known compounds were identified by comparison with literature data. For **195**, IR, ^1^H and ^13^C NMR identified a carbonyl, an hydroxyl, an olefin, an oxygenated methine and five methyl groups. Further NMR data analysis (including COSY, HMBC and nOe) and comparison with **192** established the structure. nOe correlations between H-19 and Me-25 established the β-orientation of the former. Neurotrophic activity was tested using PC-12 (pheochromocytoma) cells. At the concentration of 50 μg/mL **196**, **194**, **166**, **192** and **197** induced neurite outgrowth in PC-12 cells (68, 65, 58, 50 and 24% of the cells underwent outgrowth), but **195** was inactive.

**Figure 58:**
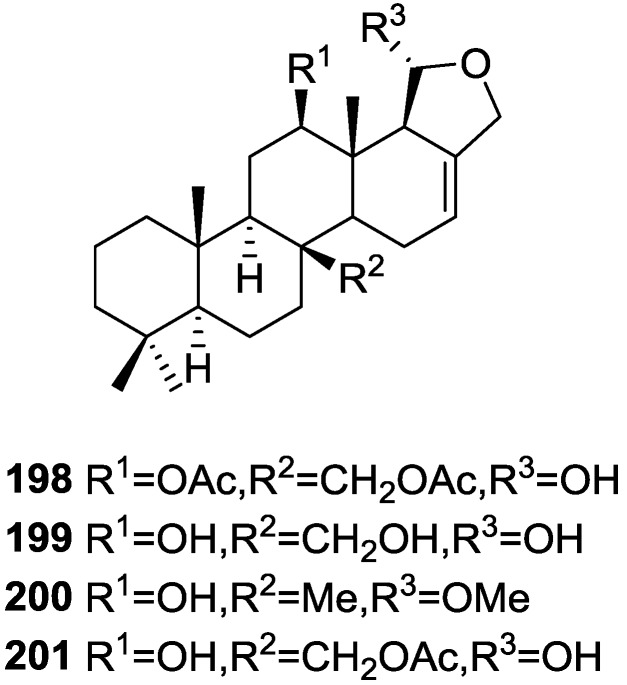
Structures of 12,24-diacetoxy-deoxoscalarin **198**, 12-*O*-deacetoxyl-24-hydroxyl-deoxoscalarin **199**, and 12-*O*-deacetoxyl-19-*O*-methyldeoxoscalarin **200**, and compound **201**.

Nam et al. [79] reported the isolation of three new sesterterpenes 12,24-diacetoxy-deoxoscalarin **198**, 12-*O*-deacetoxyl-24-hydroxyl-deoxoscalarin **199** and 12-*O*-deacetoxyl-19-*O*-methyldeoxoscalarin **200**, together with the known **201** and **165**, from a *Spongia* sp. collected in Korea (Figure 58).

The structure of **198** was established by IR, ^1^H and ^13^C NMR, DEPT, COSY, HSQC, HMBC and NOESY, where olefin, acetal and acetate groups were revealed. A scalarane type skeleton with acetyl groups at C-12 and C-24 was thus assigned. The relative stereochemistry was established by NOESY where Me-23, C-24, Me-25 and H-19 showed the same orientation. H-18 was shown to be *trans* to H-19 by the *J* coupling observed and correlation with H-12 showed that both have a *cis* relationship (confirmed by *J* coupling value of the latter). Comparison of **199** with **198** showed that both have the same skeleton, the only difference being that two hydroxyl groups of the former are replaced by acetyl groups in the latter, as evidenced by the upfield shifts of H-12 and H-24. Synthesis of the triacetate of both compounds confirmed the assignment. The spectral data of **200** showed, when compared to **199**, extra methoxyl and methyl groups and the loss of the downfield C-24 methylene. HMBC confirmed the location of the methoxyl group. All compounds were tested against FXR (nuclear hormone receptor, farnesoid X-activated receptor, a promising drug target to treat hypercholesterolemia in humans) transactivation (Table 3). The authors suggest that acetyl groups at C-12 and C-24 are critical for FXR antagonistic activity.

**Figure 59:**
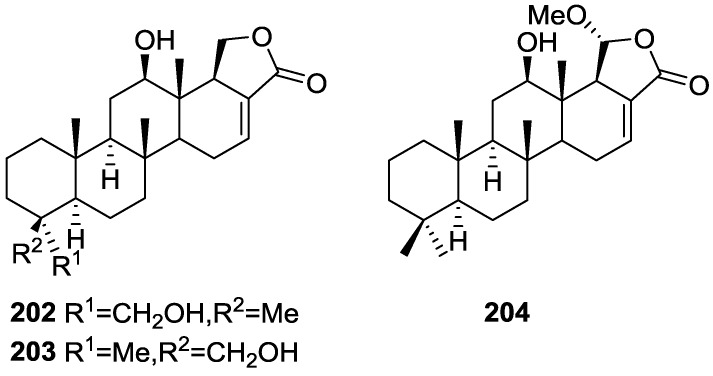
Structures of 12-*O*-deacetyl-12-*epi*-19-deoxy-21-hydroxyscalarin **202**, 12-*O*-deacetyl-12-*epi*-19-deoxy-22-hydroxyscalarin **203**, and 12-*O*-deacetyl-12-*epi*-19-*O*-methylscalarin **204**.

Further studies of Nam et al. [80] on the activity of extracts as FXR transactivation inhibitors led to the isolation of the new 12-*O*-deacetyl-12-*epi*-19-deoxy-21-hydroxyscalarin **202**, 12-*O*-deacetyl-12-*epi*-19-deoxy-22-hydroxyscalarin **203** and 12-*O*-deacetyl-12-*epi*-19-*O*-methylscalarin **204** (Figure 59), together with the known 12-*O*-deacetyl-12-*epi*-scalarin **192**, 12-*epi*-scalarin **166** and 12-*O*-deacetyl-12-*epi*-19-deoxyscalarin **194**, from a *Spongia* sp. collected in the South sea of Korea.

Compound **202** was assigned by IR, ^1^H and ^13^C NMR, COSY, HSQC, HMBC and ROESY: IR and ^13^C data indicated an hydroxyl and α,β-exounsaturated-γ-lactone; an oxygenated methylene at C-19 was assigned by COSY (coupling to H-18) and HMBC, and an oxygenated methylene at C-21 was assigned by HMBC. The relative stereochemistry was assigned by *J* coupling analysis and ROESY that showed that Me-22, Me-23, Me-24, Me-25 were axial, and were on the same plane as C-19. The magnitude of the *J* coupling of H-12 indicated also an axial orientation. The spectral data for **203** showed an identical structure to **202** except for ROESY correlations that established it was a diastereomer. The spectral data for **204** indicated a scalarane skeleton with a methoxy group at C-19. The location of the acetal, the γ-position of the lactone and the position of the double bond were determined by HMBC. The relative stereochemistry was assigned by *J* coupling and NOESY data that established H-12 as axial (correlation with H-18). The α-configuration of C-19-OMe was established by a NOESY correlation between H-19 and Me-25. All compounds were tested against FXR transactivation (Table 4). The stereochemistry at C-4 seems to be critical for biological activity since compounds **203**, **194**, **192** and **166** showed almost no activity below the IC_50_ value of cytotoxicity.

Direct binding of scalarins **202**–**204**, **194**, **192** and **166** to the ligand binding domain (LBD) of FXR was monitored by using surface plasmon resonance (SPR) spectroscopy using a BIAcore system. They decreased the affinity of FXR LBD for SRC-1 peptide, which was facilitated by CDCA (chenodeoxycholic acid, a natural ligand for FXR). **202**, which showed the most potent antagonistic activity against FXR in the cell-based cotransfection assay, was only a weak inhibitor or no inhibitor at all of the specific interaction between FXR and SRC-1 peptide. This result suggested that **202** should inhibit FXR transactivation by an indirect mechanism or by interaction with one of the other cofactors such as SRC-2or-3 in cells, which was not tested. Compounds **204**, **192**, **166** and **194** showed very strong direct interactions with FXR, although they were not especially potent in the cell based assay. This may be caused by the fact that FXR controls target gene expression in a ligand- and promoter-specific fashion. They may interact with FXR very well as on a natural promoter such as a bile salt export pump or cholesterol 7α-hydrolase, a well known target for FXR, while they activate the luciferase reporter gene poorly on a nonmammalian promoter, ecdysone receptor response element.

**Figure 60:**
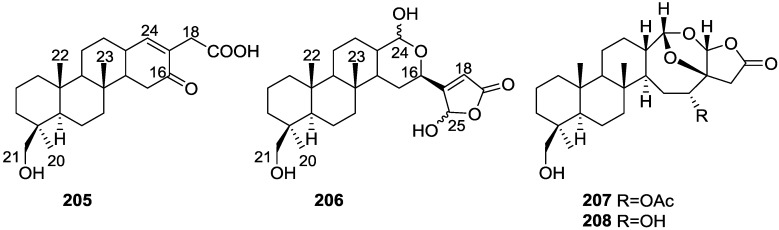
Structures of 21-hydroxy petrosaspongiolide K **205**, 21-hydroxy petrosaspongiolide P **206**, petrosapongiolides D **207**, and G **208**.

From the already mentioned study of De Marino et al. [42] on a *Spongia* sp. collected in Vanuatu Islands, Australia, 21-hydroxy petrosaspongiolide K **205** and 21-hydroxy petrosaspongiolide P **206**, together with the known petrosapongiolides D **207** and G **208** were isolated (Figure 60). Compounds **205** and **206** are the 21-hydroxy derivatives of the known petrosaspongiolides K and P, respectively.

For **205** comparison with the know petrosaspongiolides K led to the proposed structure. The localization of the hydroxyl group was confirmed by HMBC. For **206** the comparison with petrosaspongiolide P led to the proposed structure. The upfield shift for C-3 and the presence of an oxygenated methylene carbon were consistent with the proposed structure. For **205** mass spectrum, IR and ^13^C NMR data indicated the presence of a carboxyl group. The ^13^C NMR data also revealed a tricarbocyclic skeleton with geminal dimethyl groups at C-4, and two methyl groups at the ring junctions C-8 and C-10. A disubstituted pyridinium salt was also inferred from ^13^C NMR, confirmed by UV and IR absorptions typical of alkylpyridinium salts. COSY and HMBC allowed the proposal of the structure. For **206** the comparison with **205** allowed the identification of the acetoxymethyl group, located at C-4 by the downfield shift observed at C-4 and upfield shift observed at C-3. The stereochemistry at C-4 was determined by ROESY (intense cross peaks between CH_2_-17 and Me-19). For **207** ^1^H and ^13^C NMR, COSY and HMBC data, together with comparison with **205** and **206** allowed the determination of the proposed structure. For **208** the comparison with **205** and the differences observed for the pyridine salt moiety, together with COSY and IR data allowed the determination of the taurine residue. HMBC established its location. . Inhibition of specific PLA_2_ enzymes constitutes a potentially useful approach for treating a great variety of inflammatory disorders. Compounds **205** and **206** were tested as inhibitors of sPLA_2_ (secretory phospholipase A_2_) enzymes belonging to the groups I (*Naja naja* venom and porcine pancreatic enzymes), II (human synovial recombinant and rat air pouch secretory enzymes) and III (bee venom enzymes). Compound **206** inhibited preferentially human synovial PLA_2_ in the μM range, showing a slightly lower potency towards this enzyme that that of the reference inhibitor, manoalide. None of the compounds was active against cPLA_2_ (cytosolic phospholipase A_2_ from macrophage cell line RAW 264.7), although this enzyme was partially inhibited by manoalide at 10 μM. The results are shown in Table 5.

**Figure 61:**
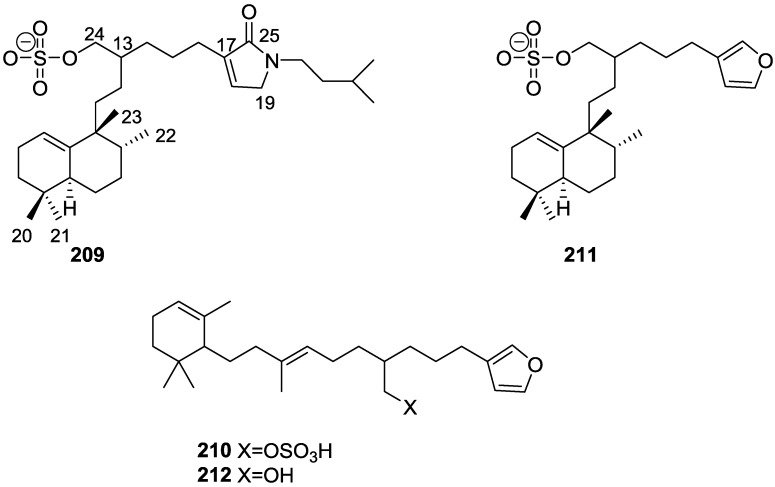
Strutures of irregularasulfate **209**, hipposulfate C **210**, halisulfate-7 **211**, and igernellin **212**.

Carr et al. [81] reported the isolation of a new nitrogen containing sesterterpenoid irregularasulfate **209**, together with the known hipposulfate C **210**, halisulfate-7 **211** and igernellin **212** (Figure 61), from the bioassay guided fractioning of extracts of *S. irregularis* Ledenfeld from Papua New Guinea.

Compound **212** was isolated from an inactive fraction. Compounds **210**, **211** and **212** were identified by comparison with literature data. For **209**, comparison of the NMR data (^1^H, ^13^C, DEPT, COSY, HSQC, HMBC) with that of halisulfate-7 **211** showed the molecules were closely related. HMBC correlations indicated the attachment of C-16 to the α-carbon of an α,β-unsaturated-γ-lactam. COSY and HMBC identified the remaining isopentenyl fragment, whose attachment to nitrogen was confirmed by HMBC. The near identity of the carbon and proton shifts of the decalin system of **209** and **211** showed that the relative configurations at C-5, C-8 and C-9 were identical. The relative configuration at C-13 remained to be assigned in both compounds. Calcineurin (a serine/threonine protein phosphatase) is the indirect cellular target of two important immunosuppressive natural product drugs. Irregularasulfate **209** inhibited calcineurin in vitro with IC_50_ of 59 μM, while hipposulfate C **210** and halisulfate **211** showed calcineurin inhibition with IC_50_’s of 66 and 69 μM, respectively. To test for selectivity, **210** and **211** were also tested against pure preparations of the catalytic subunits of protein phosphatases PP-1 and PP-2A. They both showed similar potency against PP-1 (IC_50_’s 71 and 64 μM, respectively) as they did against calcineurin, but were less active to PP-2A (IC_50_’s of 130 and 140 μM, respectively). It seemed possible that the sulfated C-24 hydroxymethyl fragment in **209**, **210** and **211** mimicked a phosphorylated serine residue in the natural substracts for calcineurin, PP-1 and PP-2A. In order to test this further, thiophosphate and phosphate analogues of the major *S. irregularis* metabolites hipposulfate C **210** and halisulfate-7 **211** were prepared in an attempt to increase their potency as calcineurin inhibitors. The phosphate analogue of **211** was significantly less active than **211** against calcineurin (IC_50_ 36 μM) but was more active against PP-1c (IC_50_ 36 μM). A possible explanation was that the analogue acted as substrate and was being converted to the inactive alcohol during the assay. The thiophosphate analogue of **210** showed comparable activity (IC_50_ 70 μM) against calcineurin to that of **210**. The sulfate functionality seems to be essential for phosphatase inhibition.

### Other Studies

**Figure 62:**
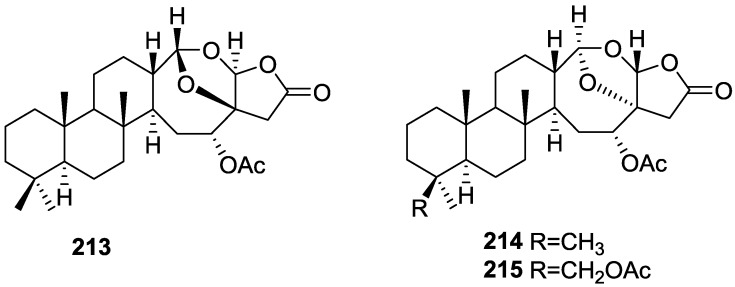
Structures of petrosaspongiolides A **213**, B **214**, and I **215**.

Petrosaspongiolides A **213**, B **214** and I **215** were isolated from a *Spongia* sp. collected in Vanuatu Islands, Australia [82] (Figure 62).

**Figure 63:**
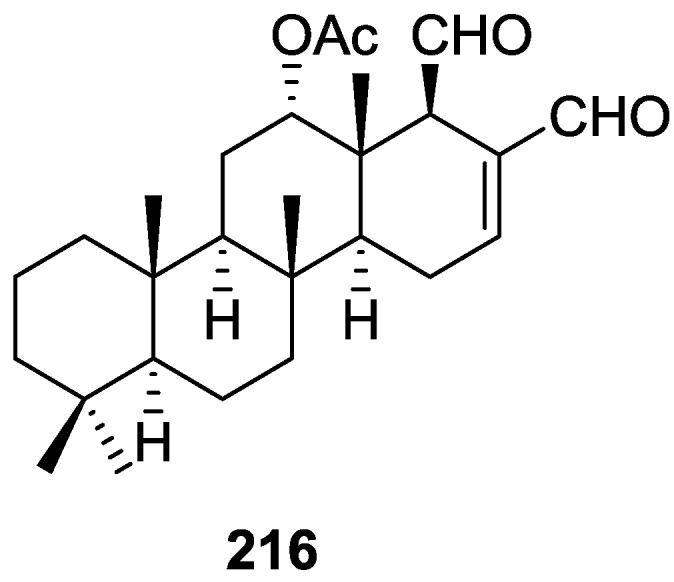
Structure of scalaradial **216**.

Deoxoscalarin **163**, 16-deacetoxy-12-*epi*-scalarafuran acetate **174** and scalaradial **216** (Figure 63), were isolated from the already mentioned study on *S. officinalis* L. from Sicily of Manzo et al. [66]

## 6. Sterols

**Figure 64:**
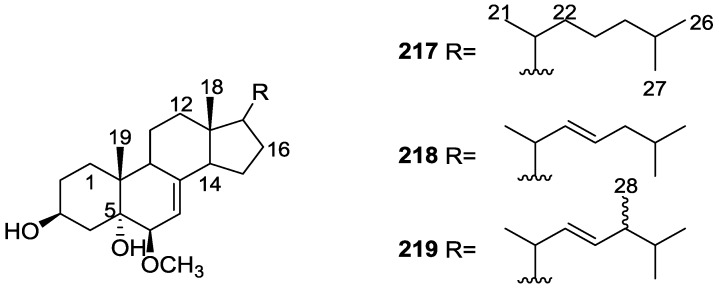
Structures of 3β,5α-dihydroxy-6β-methoxycholest-7-enes **217**, **218**, and **219**.

Aiello et al. [83] reported the isolation of three new 3β,5α-dihydroxy-6β-methoxycholest-7-enes from *S. agaricina* collected in Naples (Figure 64).

Compound **217** was identified by ^1^H and ^13^C NMR were the steroid skeleton was recognized by the five methyl resonances. The high field position of Me-18 in ^13^C NMR was in good agreement with a ∆^7^ sterol. Further evidence came from spin-decoupling experiments. The multiplicity of H-4 axial suggested that C-5 was not protonated. This fact, together with the downfield shift of H-3 relative to 5α-cholestan-3β-ol and the downfield shift observed for H-3 when the spectra was run in pyridine, led to assignment of the 5α-hydroxyl group. Spin decoupling experiments led to the oxidation pattern of ring B. Selective esterification of C-3-OH confirmed that the remaining hydroxyl group was located at C-5. The configuration at C-6 was established by *J* coupling analysis and was consistent with the lower than normal resonances of Me-19 and axial H-4. For **218** the comparison with **217** led to the assignment. The presence of a double bond in the side chain was inferred from mass spectrum and ^1^H NMR aided by spin decoupling experiments. The *E* configuration was established by the large coupling constant of H-22 and H-23. For **219** the comparison with **217** and **218** led to the assignment. The possibility that the compounds were formed by methanolysis of the allylic alcohol during extraction was excluded by repeating the extraction with EtOH.

**Figure 65:**
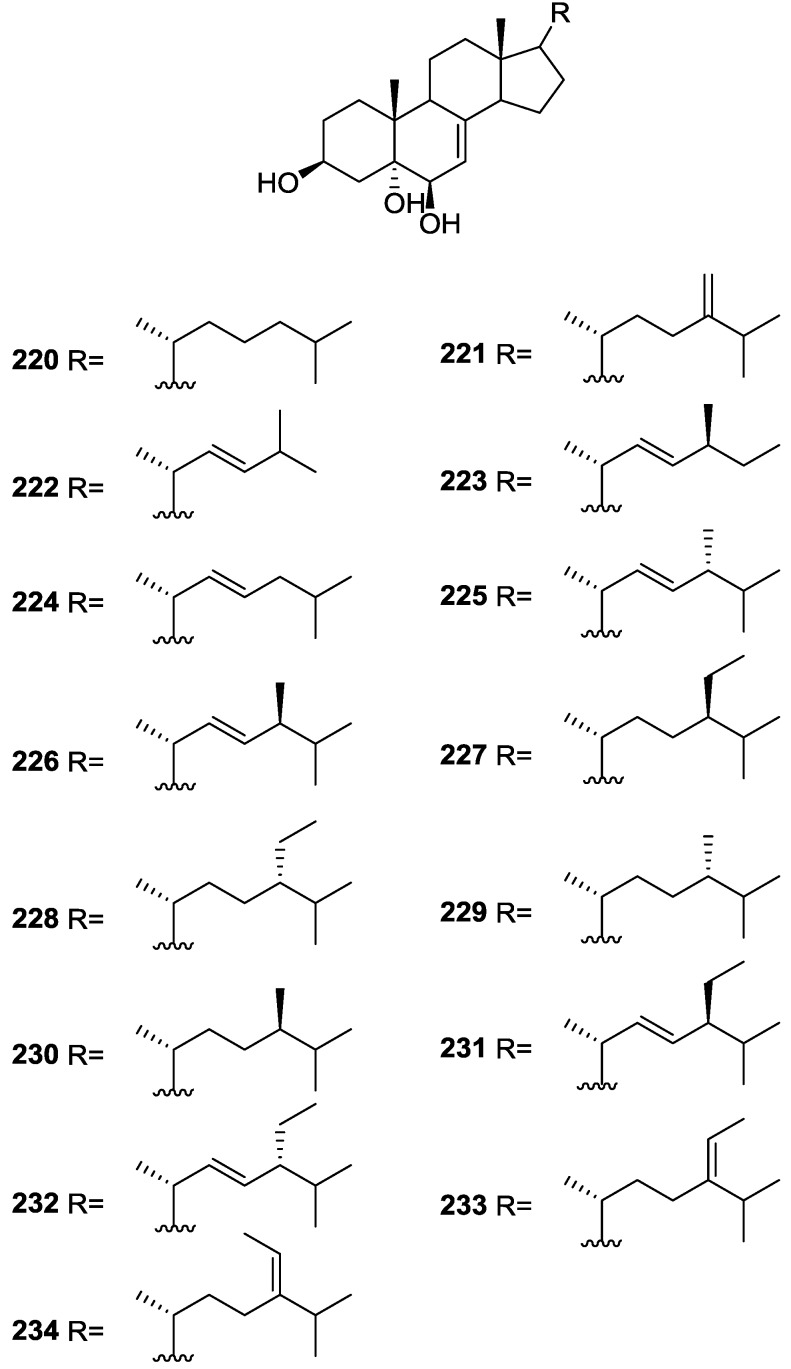
Structures of 3β,5α,6β-trihydroxycholest-7-enes **220**–**234**.

Madaio et al. [84] reported the isolation of the known **220**–**228** together with the new **229**–**234** 3β,5α,6β-trihydroxycholest-7-enes from *S. officinalis* collected in Naples (Figure 65).

The 3β,5α,6β-trihydroxy sterol nucleus was identified by the chemical shift values of H-3, H-4, H-6, H-7, Me-18 and Me-19. In addition the signals of H-3, axial H-4 and Me-19 showed the typical pyridine induced deshielding due to the 1,3-diaxial interaction with C-5 and C-6 hydroxyl groups. The ^13^C NMR shifts of C-3, C-5, C-6, C-7 and C-8 confirmed the assignment. For all the compounds, a [M^+^] could not be observed, the highest peak in mass spectra being at *m/z* values corresponding to [M − H_2_O]^+^. The new compounds were all isolated as mixtures (**229** and **230**, **231** and **232**, **233** and **234**). For **229** and **230** analysis of the mass spectra allowed the identification of a saturated C_9_H_19_ side chain. The chemical shifts of the side chain signals were consistent with those of authentic samples of campesterol and 24-*epi*-campesterol. For **231** and **232** the mass spectra allowed the identification of a C_10_H_19_ side chain containing a double bond; ^1^H–^1^H COSY 45 and irradiation allowed the assignment of the ^1^H NMR shifts. Comparison of the methyl region of the ^1^H NMR spectra with that of a 1:1 mixture of stigmasterol and poriferasterol revealed the presence of both epimers. For **233** and **234** the mass spectra allowed the identification of a C_10_H_19_ side chain containing a double bond and ^1^H decoupling experiments confirmed the presence of the ethylidene group at C-24. The side chain chemical shifts were consistent with those of fucosterol and 28-*iso*-fucosterol; the stereochemistry of the 24(28) double bond was determined by the chemical shift value of H-25, in accordance with the values reported for the known compounds.

**Figure 66:**
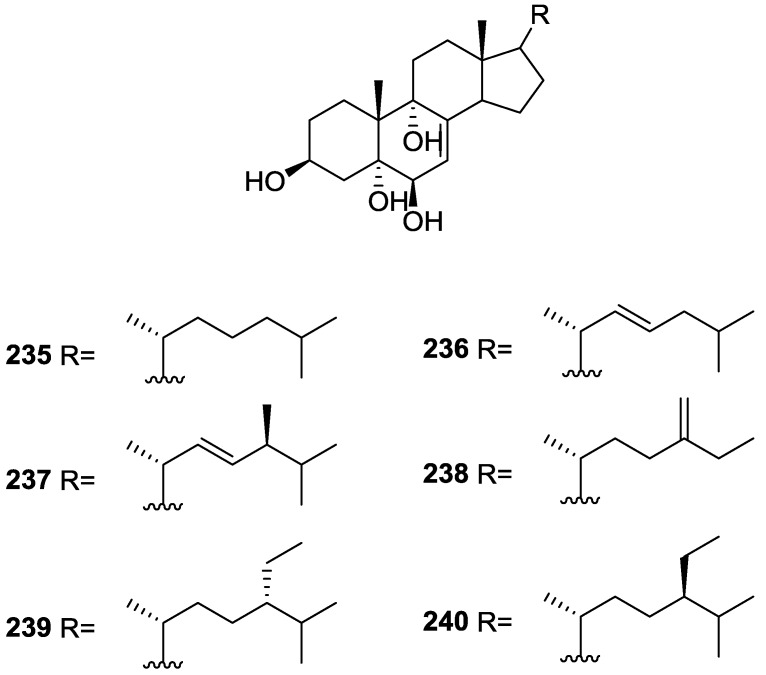
Structures of 5α-cholest-7-ene-3β,5,6β,9-tetraol **235**, (22*E*)-5α-cholest-7,22-diene-3β,5,6β,9-tetraol **236**, (22*E*,24*S*)-24-methyl-5α-cholest-7,22-diene-3β,5,6β,9-tetraol **237**, 24-methylene-5α-cholest-7-ene-3β,5,6β,9-tetraol **238**, (24*S*)-24-ethyl-5α-cholest-7-ene-3β,5,6β,9-tetraol **239**, and (24*R*)-24-ethyl-5α-cholest-7-ene-3β,5,6β,9-tetraol **240**.

Work of Migliuolo et al. [85] allowed the isolation of six new tetrahydroxylated sterols, 5α-cholest-7-ene-3β,5,6β,9-tetraol **235**, (22*E*)-5α-cholest-7,22-diene-3β,5,6β,9-tetraol **236**, (22*E*,24*S*)-24-methyl-5α-cholest-7,22-diene-3β,5,6β,9-tetraol **237**, 24-methylene-5α-cholest-7-ene-3β,5,6β,9-tetraol **238**, (24*S*)-24-ethyl-5α-cholest-7-ene-3β,5,6β,9-tetraol **239** and (24*R*)-24-ethyl-5α-cholest-7-ene-3β,5,6β,9-tetraol **240** from *S. officinalis* collected in Naples (Figure 66).

The mass spectra, ^1^H and ^13^C NMR data of all compounds allowed the identification of a common skeleton, thoroughly determined for **235** by NMR analysis of the ^1^H spectra run in CD_3_OD and pyridine, together with ^13^C NMR, DEPT, COSY 45, irradiation experiments and homo-decoupling spectral measurements. Five methyl signals of the cholestane skeleton were observed and the signal of H-3 showed the normal complexity of an α-oriented proton for an A/B *trans* steroid; its low field chemical shift was typical of 3β-hydroxysteroids with a 5α-hydroxyl group. The downfield shifts observed in pyridine for H-1 axial, H-3 axial, H-4 axial and Me-19 led to the placement of two hydroxyl groups in the 6β and 9α positions. The side chain structure was established by comparison of the ^13^C NMR data with that of cholesterol. nOe difference experiments confirmed the proposed structure and allowed the assignment of the overall relative stereochemistry: irradiation of 9α-OH enhanced axial H-1 and H-14; irradiation of H-3 enhanced equatorial H-2 and equatorial H-4; irradiation of Me-19 caused nOe on 6β-OH confirming its orientation. Chirality at C-17 was also determined by nOe that proved Me-18 and Me-21 are in proximity. For **236** and **237**, analysis of the mass spectra and NMR data allowed the identification of the side chain. The *E* stereochemistry of both double bonds was established on the basis of the *J* coupling observed for H-22/H-23. For **237** the assignment of the configuration at C-24 was performed by comparison with an epimeric mixture of brassicasterol. For **238** the identity of the side chain was established by mass spectrometry. NMR data analysis, together with comparison of the ^13^C NMR shifts with those of 24-methylene-5α-cholest-7-ene-3β,6β-diol allowed its confirmation. Compounds **239** and **240** could not be separated, and NMR data analysis was difficult on account of signal overlapping. The chemical shifts of the side chain carbons and methyl protons, as well as the absolute configuration at C-24, were established by comparison with those of sitosterol and clionasterol. The authors also point out that, analogously to what had been stated by other authors on the biosynthesis of another organism, the co-occurrence in the same sponge of the isolated metabolites together with ∆^7^-3β,5α,6β-trihydroxysterols and the corresponding ∆^5,7^-3β-hydroxysterols, may indicate that the latter may be the biosynthetic precursors of both the above mentioned ∆^7^-tri and ∆^7^-tetrahydroxysterols.

**Figure 67:**
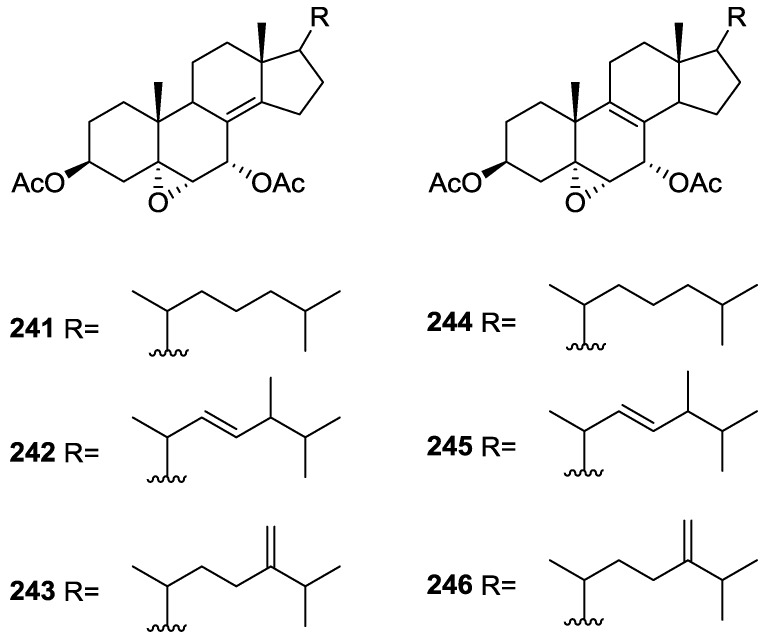
Structures of 5α,6α-epoxycholest-8(14)-ene-3β,7α-diol 3,7-diacetate **241**, (22*E*,24ξ)-5α,6α-epoxy-24-methylcholesta-8(14),22-diene-3β,7α-diol 3,7-diacetate **242**, 5α,6α-epoxy-24-methylcholesta-8(14),24(28)-diene-3β,7α-diol 3,7-diacetate **243**, 5α,6α-epoxy-cholest-8-ene-3β,7α-diol 3,7-diacetate **244**, (22*E*,24ξ)-5α,6α-epoxy-24-methylcholesta-8,22-diene-3β,7α-diol 3,7-diacetate **245**, and 5α,6α-epoxy-24-methylcholesta-8,24(28)-diene-3β,7α-diol 3,7-diacetate **246**.

Subsequent work by Migliuolo et al. [86] on a Mediterranean *S. officinalis* led to the isolation of the diacetate derivatives of six new sterols, 5α,6α-epoxycholest-8(14)-ene-3β,7α-diol 3,7-diacetate **241**, (22*E*,24ξ)-5α,6α-epoxy-24-methylcholesta-8(14),22-diene-3β,7α-diol 3,7-diacetate **242**, 5α,6α-epoxy-24-methylcholesta-8(14),24(28)-diene-3β,7α-diol 3,7-diacetate **243**, 5α,6α-epoxy-cholest-8-ene-3β,7α-diol 3,7-diacetate **244**, (22*E*,24ξ)-5α,6α-epoxy-24-methylcholesta-8,22-diene-3β,7α-diol 3,7-diacetate **245** and 5α,6α-epoxy-24-methylcholesta-8,24(28)-diene-3β,7α-diol 3,7-diacetate **246** (Figure 67).

Isolation of the compounds in pure form proved very difficult and they could only be separated after acetylation. Previous NMR analysis indicated the absence of natural acetyl groups. For **241** mass analysis after hydrolysis established the molecular formula and the presence of two hydroxyl groups in the natural compound. NMR analysis of **241** allowed the identification of the structure. The ∆^8,(14)^ double bond was established by the chemical shift values of Me-18, C-8 and C-14. The chemical shift and shape of the signal of the 3α proton was in accordance with an A/B *trans* 3β-acetoxysteroid having a oxygenated function at C-5 with an α-orientation, confirmed by COSY spectra. This function was identified as an epoxide ring (^1^H and ^13^C NMR resonances). The second acetoxy group was located at C-7 since H-7 correlated with H-6. The cholestane type side chain was indicated by NMR and mass data. nOe experiments failed to establish the stereochemistry at C-7. The proposed structure was confirmed by X-ray analysis. Comparison of **242** and **243** with **241** showed that these compounds only differed in the side chain. These were established by MS data and NMR, including decoupling experiments. For **242** the configuration at C-24 remained unassigned. For **244** the mass spectrum and NMR data were very similar to **241**. The positioning of all functionalities in the steroid nucleus was aided by ^1^H–^1^H COSY-60 and decoupling experiments. The α-orientation of the acetate group at C-7 was indicated by nOe between H-7 and Me-19. The double bond could only be located at the ∆^8^ position. Again comparison of **245** and **246** with **244** showed that these compounds only differed in the side chain. These were established by mass data and decoupling experiments. For **245** the configuration at C-24 remained unassigned.

**Figure 68:**
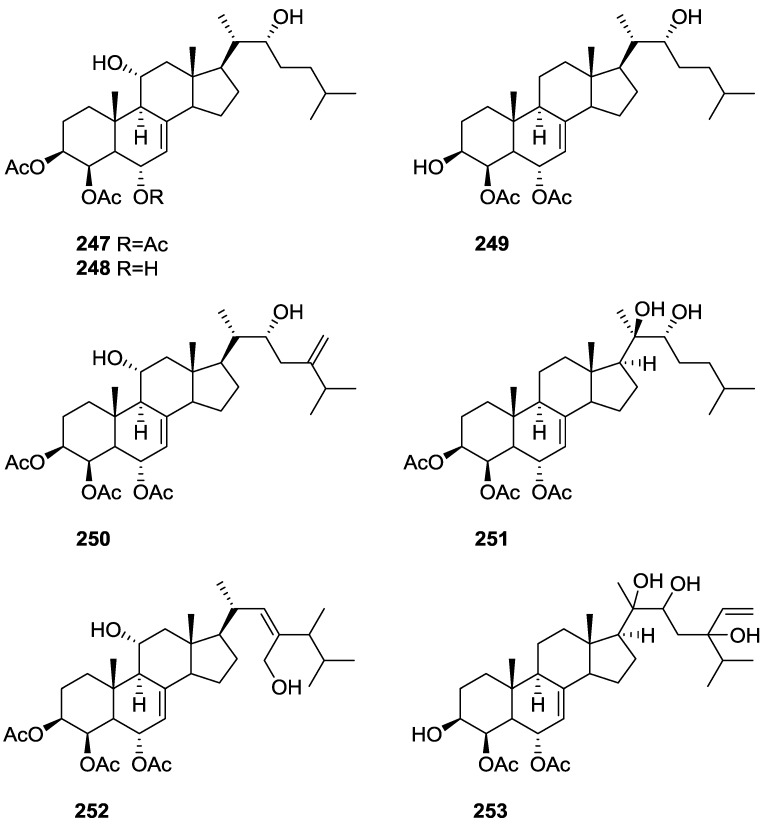
Structures of agosterol A **247**, B **248**, C **249**, A_4_ **250**, D_2_ **251**, A_5_ **252** and C_6_ **253**.

Aoki et al. [87] reported the bioassay-guided isolation of agosterol A **247** from a *Spongia* sp. collected in Mie Prefecture. The ethyl acetate soluble portion of the acetone extract showed strong growth inhibition at 10 μg/mL against P-gp (P-glycoprotein) overexpressing MDR tumor cells (KB-C2) in the presence of 0.1 μg/mL of colchicine, while it exhibited little cytotoxicity against parental KB-3-1 cells at 10 μg/mL. Further work [88] led to the isolation of agosterol B **248**, C **249**, A_4_ **250**, D_2_ **251**, A_5_ **252** and C_6_ **253** together with agosterol A **247** (Figure 68).

Compound **247** was identified on the basis of ^1^H and ^13^C NMR, COSY, TOCSY and HMBC spectral data. The relative stereochemistry was assigned on the basis of ROESY spectra and *J* couplings. The absolute configurations at C-11 and C-22 were established as *R* by a modified Mosher’s method. The configuration at C-20 was determined as *S* by comparison of the CD data of the 22-keto derivative (obtained by treatment of **247** with pyridinium dichromate) to that of (20*S*)-22-ketocholesterol.

The compounds **248**–**253** were analyzed by 2D NMR (COSY, HMQC, HOHAHA, HMBC and NOESY), compared to agosterol A **247**, and their structures were elucidated. Compound **248** was very similar to **247**, the difference being the lack of an acetyl group at C-6 (indicated by the higher field resonances for H-6 and C-6). For **249** the lack of the 3-*O*-acetyl group and higher field resonances of protons and carbons at C-3 and C-11 led to the proposed structure. For **250** the exomethylene group was located in C-24 by HMBC; the absolute configuration at C-22 was assigned as *R* by a modified Mosher’s method. The 20*S* configuration was presumed on the basis of the similarity of the C-21 shift with that of **247**. For **251**, treatment with 2,2-dimethoxypropane and PPTS afforded the acetonide derivative. Comparison of the Me-21 ^1^H chemical shifts of both compounds established an 20*R*,22*R* configuration. The absolute configuration at C-22 was confirmed by a modified Mosher’s method. For **252** the *E* geometry of the ∆^22^ double bond was assigned by NOESY (correlation between H-22, H-24 and Me-28). The absolute stereochemistry at C-20 and C-24 were not determined. For **253** the side chain was identified by ^1^H NMR and HMBC. The absolute stereochemistry at C-20, C-22 and C-24 were not determined. Agosterol A **247** completely reversed the resistance to colchicine in KB-C2 cells (P-gp overexpressing strain) at 3 μg/mL and also the resistance of vincristine in KB-CV60 cells (which overexpress multidrug resistance-associated protein MRP), at 1 μg/mL. Compound **247** was not cytotoxic even at 10 μg/mL. The reversing activity of the analogous **248**–**253** and other derivatives were further examined. The results also showed that three acetyl groups in rings AB seem to be crucial for reversing activity. The C-11 and C-22 hydroxyl groups also seem to be crucial. Further studies on the mechanism by which agosterol A **247** reverses MRP I-mediated drug resistance through the investigation of the interaction between agosterols and MRP I in MRP I-over expressing human KB carcinoma cells were undertaken [89].

**Figure 69:**
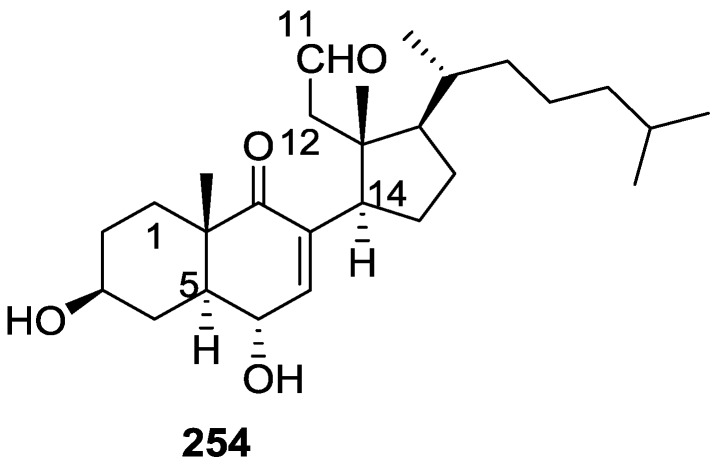
Structure of 9,11-secosterol, 3β,6α-dihydroxy-9-oxo-9,11-seco-5α-cholest-7-en-11-al **254**.

Migliuolo et al. [90] reported the isolation of the new 9,11-secosterol, 3β,6α-dihydroxy-9-oxo-9,11-seco-5α-cholest-7-en-11-al **254**, from *S. officinalis* L. collected in Naples (Figure 69).

The structure of **254** was elucidated with mass spectrometry, UV, IR, ^1^H and ^13^C NMR, ^1^H–^1^H COSY, proton double quantum 2D NMR, ^1^H–^13^C heterocorrelation and decoupling experiments. The conjugated ketone was identified by UV and IR. Five methyl resonances indicated a steroid nucleus. The H-3 signal indicated a 3β-hydroxysteroid with A/B *trans* rings and the stereochemistry at C-6 was inferred from the *J* coupling values; the fact that C-6-OH was equatorial was indicated by the small downfield shift observed for Me-19, and the more substantial downfield shift on H-4 equatorial, when the proton spectrum was run in pyridine-d_5_. The localization of the CH_2_CHO fragment came from nOe that also fully supported the seco structure for ring C: irradiation of Me-18 caused effects on Me-19 and Me-21, and a strong effect on the H-7 signal, implying that the ring-D-containing portion of the molecule was slightly rotated around the C-8/C-14 bond. Mass spectra identified the side chain, confirmed by ^13^C NMR. Further proof of the proposed structure came from its synthesis from 7-dehydrocholesterol, the first synthesis of a natural ring-C secosterol.

**Figure 70:**
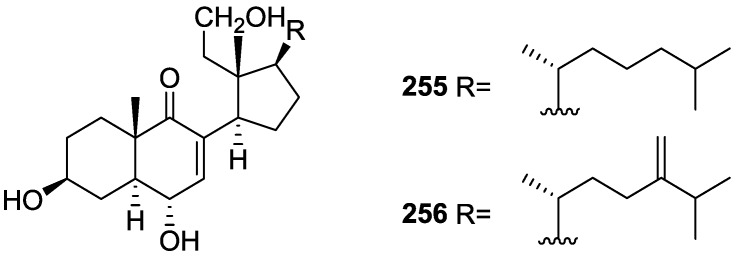
Structures of 9,11-seco-3β,6α,11-trihydroxy-5α-cholest-7-en-9-one **255**, and 9,11-seco-3β,6α,11-trihydroxy-24-methylene-5α-cholest-7-en-9-one **256**.

Further studies of the same sponge [91] led to the isolation of the new 9,11-seco-3β,6α,11-trihydroxy-5α-cholest-7-en-9-one **255** and 9,11-seco-3β,6α,11-trihydroxy-24-methylene-5α-cholest-7-en-9-one **256** (Figure 70).

For **255** the conjugated ketone was identified by UV and IR and confirmed by ^13^C NMR. Five methyl resonances indicated a steroid nucleus. The unsaturation equivalents deduced from the molecular formula indicated a secosterol structure. MS data and NMR, including ^1^H–^1^H COSY-45 indicated that the structure contained the same 9,11-seco-3β,6α-dihydroxy-7-en-9-one steroidal structure of **254** with an hydroxyl group at C-11 instead of the aldehyde group. The side chain structure was supported by MS and ^13^C NMR. Further confirmation came by synthesis from **254** by aldehyde selective reduction with tetra-*n*-butylammonium triacetoxyborohydride. Comparison of **256** with **255** showed that these compounds only differed in the side chain, which was identified by MS analysis and COSY-45. Assignment of the chemical shifts for the side chain carbons in the ^13^C NMR spectrum was based on comparison with the known values for 24-methylene-5α-cholest-7-en-3β,6α-diol.

**Figure 71:**
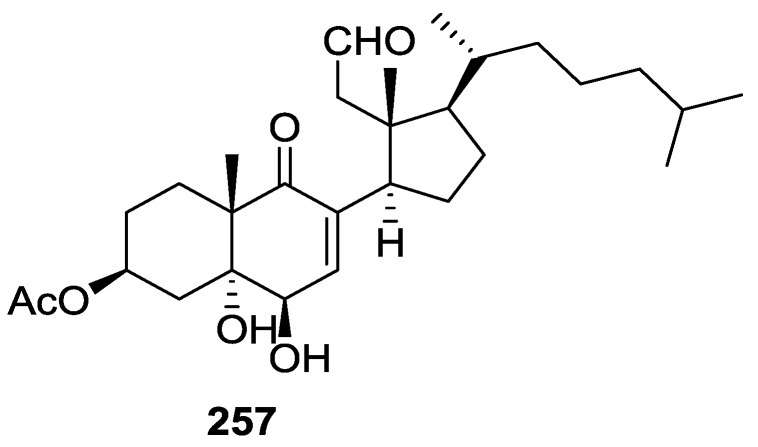
Structure of 3β-acetoxy-5,6β-dihydroxy-9-oxo-9,11-seco-5α-cholest-7-en-11-al **257**.

Further investigation of the same sponge [92], led to the isolation of 3β-acetoxy-5,6β-dihydroxy-9-oxo-9,11-seco-5α-cholest-7-en-11-al **257** (Figure 71).

Compound **257** was elucidated with mass spectrum, UV, IR, and NMR with decoupling experiments, and comparison with **254**. IR indicated an ester, α,β-unsaturated-ketone and hydroxyl functions. The enone moiety was confirmed by UV and ^13^C NMR. Four methyl resonances (one integrating for six protons) suggested a steroid of the cholestane series. The unusually high chemical shift resonance of H-3 indicated, apart from acetylation, an α-oriented hydroxyl group at C-5, which was confirmed by the multiplicity of both H-4, and the strong pyridine induced shifts experienced by the axial H-1 and H-3. The β-orientation of C-6-OH was also determined on the basis of the pyridine downfield shifts observed for Me-19 and H-4 axial. ^1^H–^1^H COSY and ^1^H–^13^C heterocorrelation fully elucidated rings A and B. nOe experiments established the location of the CH_2_CHO group and a ring-C seco structure (strong enhancement of H-7 upon irradiation of Me-18). The sidechain was identified by ^1^H NMR and mass fragments. Further confirmation of the structure came from its synthesis from 7-dehydrocholesterolacetate. The authors suggest that both **257** and **254** derive from a common 5,7,9(11)-triene sterol through oxidation at C-5 and C-6, in the case of **257**, or only at C-6 in the case of **254**, with concomitant oxidative cleavage of the 9,11 double bond.

**Figure 72:**
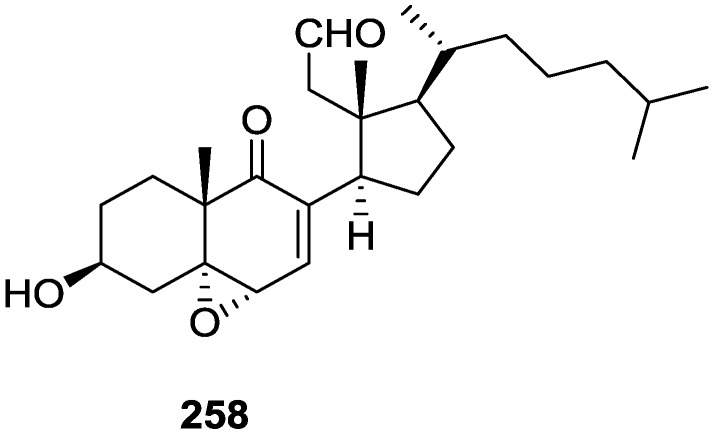
Structure of 3β-hydroxy-5α,6α-epoxy-9-oxo-9,11-seco-5α-cholest-7-en-11-al **258**.

From the already mentioned study of Lu and Faulkner [75] on *S. matamata* de Laubenfels 1954, collected in a marine lake at Palau, Western Caroline Islands, the new 3β-hydroxy-5α,6α-epoxy-9-oxo-9,11-seco-5α-cholest-7-en-11-al **258** was isolated (Figure 72).

Compound **258** was identified by comparison with the known luffasterol A, the acetyl derivative. Synthesis of this compound by acetylation of **258** confirmed the structure.

**Figure 73:**
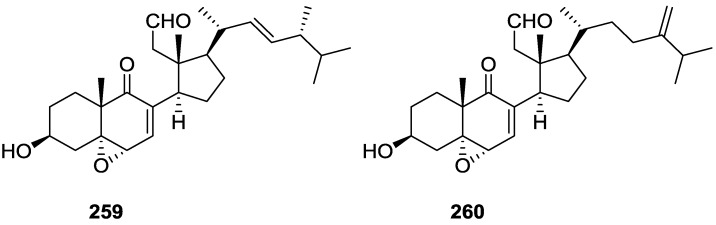
Structures of 3-*O*-deacetylluffasterol B **259** and 3-*O*-deacetyl-22,23-dihydro-24,28-dehydroluffasterol B **260**.

From the already mentioned study of Rueda et al. [67] on *S. agaricina*, from Cádiz, Spain, the new 3-*O*-deacetylluffasterol B **259** and 3-*O*-deacetyl-22,23-dihydro-24,28-dehydroluffasterol B **260** (Figure 73), together with the known **258**, were isolated.

Compound **259** was identified by spectral data and comparison with the known luffasterol B, the acetyl derivative. Synthesis of this compound by acetylation of **259** confirmed the structure. Compound **260** was identified by comparison with **259**, where the replacement of Me-28 and H-22 and H-23 proton signals by two exomethylene signals, was observed. The authors also propose that **259**, **260** and **258** derive from the same 5,7,9(11)-trienesterol precursor proposed by Adinolfi et al. [92]. Compounds **259** and **260** showed cytotoxicity against P-388, A-549, HT-29 and MEL-28 tumor cell lines with IC_50_ values of 1 μg/mL.

## 7. Macrolides

**Figure 74:**
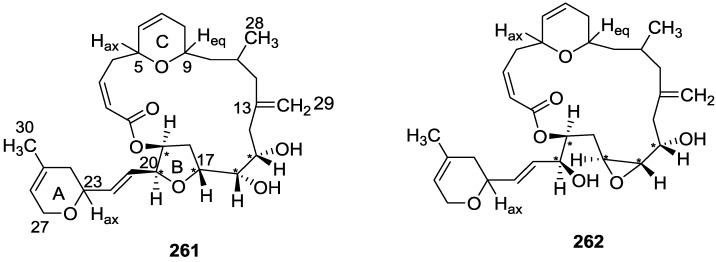
Structures of fijianolides A **261** and B **262**.

Quiñoà et al. [93] reported the isolation of two cytotoxic macrocyclic lactones, fijianolides A **261** and B **262**, from *S. mycofijiensis*, collected in Vanuatu Islands, Australia (Figure 74).

Both metabolites were identified by mass spectrometry and extensive NMR analysis. For **261** the unsaturated structures were assigned as follows: the but-2(*Z*)-enoyl array (as a lactone) was recognized by IR and NMR shifts; the dihydropyran ring A with vinylic methyl and equatorial (*E*)-ethenyl was identified by NMR resonances and the large *J* value at H-23; the second dihydropyran (ring C) with a disubstituted double bond was deduced from the value of *J*_6,7_ and the 1,3 C-ring substituents were assigned as *trans* by *J* analysis of H-5 and H-9; ^1^H–^1^H and ^1^H–^13^C COSY data allowed the proposal of a 2-methyl-4-methylenepentanyl group and the confirmation of the connections showed in **261** for the contiguous carbons. A vicinal diol was recognized by conversion to the diacetate and dioxolane and analysis of the H-15 and H-16 *J* values. Ring B was identified by ^1^H NMR chemical shifts and ^1^H–^1^H COSY spectra revealed its location. Its stereochemistry was confirmed by comparison to furanose rings and *J* value analysis. 2D nOe experiments confirmed the *cis* arrangement of H-19 and H-20. The relative H-16/H-17 *erythro* stereochemistry was assigned by comparison of *J* values with model compounds. A relative stereochemistry of 15S*,16S*,17R*,19S*,20S* is proposed for **261**. Compound **262** was identified by comparison of the NMR data with those of **261** that showed that the structure and the stereochemistry were the same except for the region of C-15 to C-20. Conversion to the diacetyl derivative allowed further identification. The *trans* epoxide ring was identified by the characteristic ^1^H and ^13^C NMR shifts and the value of *J*_16,17_. The authors suggest that both compounds are biogenetically related by an SN_2_ transposition with inversion at C-17 and that their stereochemistry is the same at the remaining centers. As such, **262** is assigned a 15S*,16S*,17S*,19S*,20S* relative configuration. Moderate in vitro cytotoxicity was shown by **261** and **262** acetate against HT-29 (IC_50_ 11 μg/mL and 0.5 μg/mL, respectively) and P388 (IC_50_ 9 μg/mL and 6 μg/mL, respectively).

**Figure 75:**
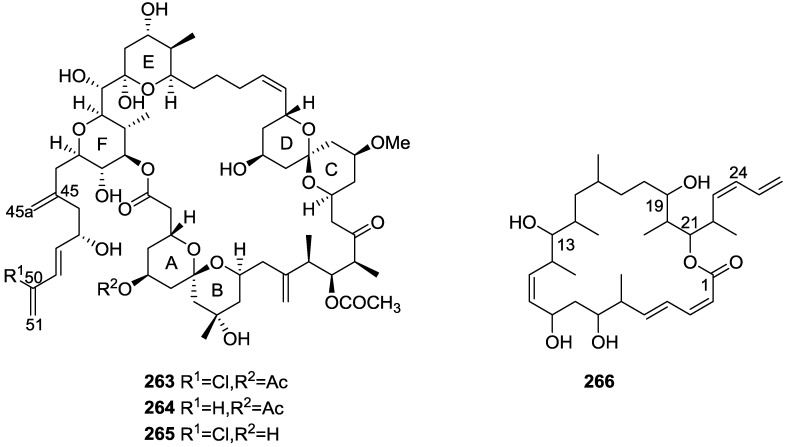
Structures of spongistatins 1–3 **263**–**265** and dictyostatin 1 **266**.

Work of Pettit et al. [94,95,96] led to the isolation of spongistatins 1–3 **263**–**265** and dictyostatin 1 **266** from a *Spongia* sp. collected in the Eastern Indian Ocean, and its recollection in the Republic of the Maldives (Figure 75).

All compounds were isolated from P388 lymphocytic leukemia bioassay active fractions. The structure of **263** was identified on the basis of extensive NMR data analysis in three solvents, at 400 and 500 MHz (including APT, ^1^H–^1^H and ^1^H–^13^C COSY, HMBC and nOe). Selective acylation experiments were also employed. Due to the small amount available and difficulties in crystallization, the relative and absolute stereochemistry was not established. Compounds **264** and **265** were identified by extensive NMR analysis and comparison with **263**. For **264** the presence of a ABX spin system in the ^1^H NMR spectra confirmed that the difference between both compounds was at C-50, confirmed by shifts observed at C-51, C-50, C-49, C-48 and C-47. For **265** the main difference relative to **263** was the absence of an acetyl group at C-5, established by the upfield shift of the corresponding H-5. The structure of **266** was identified on the basis of extensive NMR data analysis at 400 and 500 MHz (including APT, ^1^H–^1^H COSY, HMQC, HMBC and nOe). An ABX spin system in ^1^H NMR indicated a terminal unit. A broad singlet of an oxygenated methine correlating to a carbonyl in HMBC suggested a macrolide. The coupling relationships of signals corresponding to H-2, H-3, H-4 and H-5 were established and extended to H-13. Coupling of H-13 to H-19 was also established and extended to H-23 and H-26. The absence of a dihydropyran ring between C-9 and C-13 was established by nOe experiments and mass spectrometry. The geometry of the double bonds was inferred from *J* coupling values. The definitive relative and absolute stereochemistry were not assigned. At the same time, the isolation of spongistatin 1 **263** was reported, Kobayashi [97] and Fusetani [98] reported the isolation of the same metabolite from other marine sources (altohyrtin A and cinachyrolide A, respectively). The proposed configuration for spongistatin 1 **263** [96,99] was in disagreement with the proposed configuration of the other compounds [98,100]. The absolute configurations of spongistatin 1 **263** here depicted were only determined by synthesis in subsequent studies by other authors [101,102]. The authors suggest that the depicted configurations are common to all the members of this class of spongipyran natural products. Compound **263** was found to be extremely potent (GI_50_ typically 2.5–3.5 × 10^−11^ M) against a subset of highly chemoresistant tumor types (e.g., HL-60, SR leukemias; NCI-H226, NCI H23, NCI H460, NCI H522 non-small cancer lung; DMS 114 and DMS 273 small cell lung; HCT-116, HT29, KM12, KM 20L2 and SW-620, colon; SF-539, U251 brain; SK-MEL-5 melanoma; OVCAR-3 ovarian; and RXF-393 renal cancers) comprising the NCI panel of 60 human cancer cell lines. Cell lines derived from human melanoma and lung, colon, and brain cancers were found to be especially sensitive to spongistatin 1 **263**. The distinctive pattern of relative cellular sensitivity to spongistatin 1 **263** was analyzed by computerized pattern-recognition algorithms and found to be closely related with the important general mechanistic class of microtubule interactive antimitotics. Spongistatins 2 **264** and 3 **265** showed a diminished overall potency when compared to **263** in the NCI 60 cell line in vitro screening panel. Compounds **263**, **264** and **265** remain however among the most potent of substances tested in the NCI screen.

Bai et al. [103] showed that spongistatin 1 **263** inhibited mitosis, microtubule assembly and the binding of vinblastine to tubulin. Subsequent studies [104] on the interaction of macrocyclic lactone polyethers with tubulin showed that spongistatin 3 **265** inhibits the formation of the same intrachain cross-link in tubulin as is inhibited by vinblastine. Unlike vinblastine, **265** has no effect on the exposure of either sulfhydryl groups or hydrophobic areas on the tubulin molecule.

**Figure 76:**
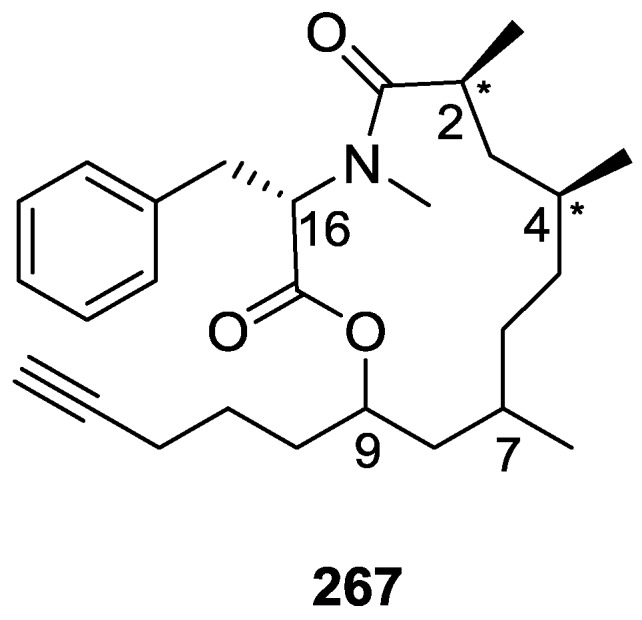
Structure of spongidepsin **267**.

From the already mentioned study of Grassia et al. [82] on a *Spongia* sp. collected in Vanuatu Islands, Australia, the new spongidepsin **267** was isolated by bioassay guided fractionation (Figure 76).

**Figure 77:**
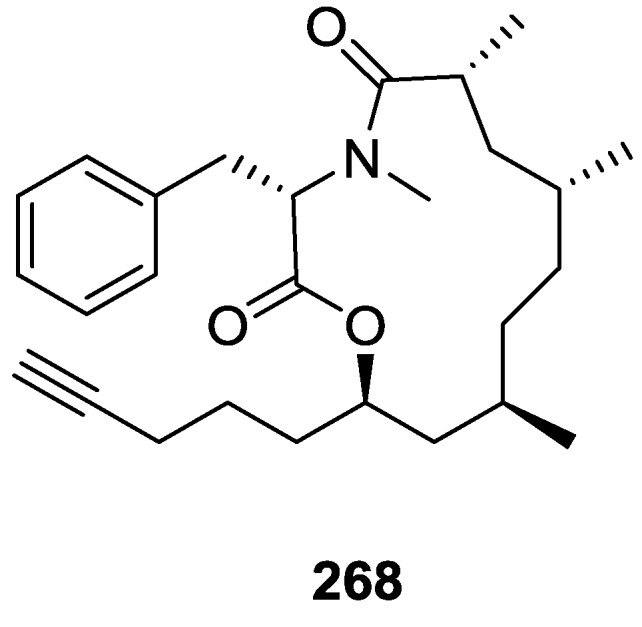
Structure of (2*R*,4*R*,7*R*,9*R*,16*S*)-spongidepsin **268**.

Compound **267** was identified through extensive NMR analysis including HSQC, HMBC, COSY and TOCSY. These spectra allowed the identification of the 9-hydroxy-2,4,7-trimethyltetradeca-14-ynoic acid. Further confirmation of the alkyne function came from ^13^C NMR resonances and HMBC data. The remaining NMR data suggested the presence of a *N*-methylphenylalanine residue. Further HMBC analysis allowed the closing of the 13-membered macrocyclic ring and determination of the carbon framework. The l-series was assigned to the *N*-methylphenylalanine residue through the application of Marfey HPLC method on the acidic hydrolysate of **267**. The relative configuration at C-2 and C-4 was investigated using an NMR approach described by Murata that relies on ^3^*J*_H,H_ and ^2,3^*J*_CH_ measurements in combination with ROESY responses, which allows the identification of the predominant conformers among the six staggered rotamers with *threo* and *erythro* relative configurations. Stereochemistry at C-7 and C-9 could not be assigned due to signal overlap that precluded *J* analysis. Subsequent work by two other authors [105,106] led to the determination of the absolute configuration spongidepsin **267** by total synthesis. Thus, (2*R*,4*R*,7*R*,9*R*,16*S*)-spongidepsin can be assigned to structure **268** (Figure 77).

Antiproliferative assays for spongidepsin **268** and of the control (6-mercaptopurine) are shown in Table 6.

## 8. Miscellaneous Compounds

**Figure 78:**
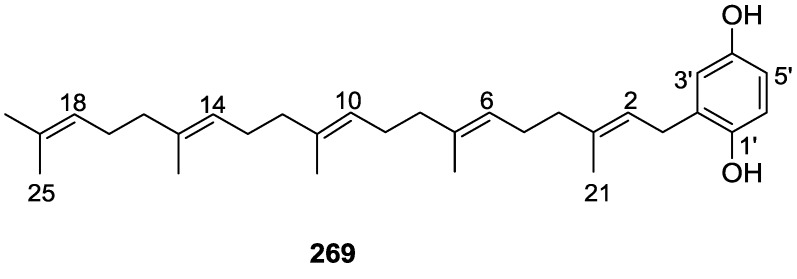
Structure of p-quinol **269**.

From the already mentioned study of Lumsdon et al. [62] on a *Spongia* sp. collected in Australia, the new p-quinol **269** was isolated (Figure 78).

NMR analysis identified a 1,2,4-trisubstituted aromatic ring, four internal trisubstituted double bonds and one terminal trisubstituted double bond of a pentaprenyl side chain with the corresponding methyl groups. The ^13^C NMR shifts of the vinylic methyls established an all *E* geometry of the double bonds while the doublet multiplicity of the benzylic methylene due to coupling to the olefinic proton allowed the double bond regiochemistry to be assigned. The substituents on the aromatic ring were supported by MS data and IR absorptions. Comparison with literature compounds supported the aromatic pattern shown. Although the crude extract evoked a large triphasic contraction of smooth muscle in the isolated guinea-pig ileum, appeared to inhibit contractions elicited by different drugs (acetylcholine, 5-hydroxytryptamine and histamine) of the isolated guinea-pig ileum, and inhibited the growth of several bacteria (*Staphylococcus* *aureus*, *Micrococcus* sp. and *Serrata* sp.) in a standard antibiotic disk assay, **269** showed no activity.

**Figure 79:**
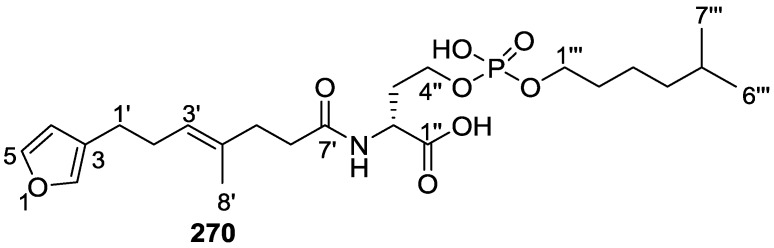
Structure of pokepola ester **270**.

Kalidindi et al. [107] reported the isolation of pokepola ester **270** from *S. oceania* collected in Maui, Hawaii (Figure 79).

The structure was identified by mass spectrometry, IR and NMR (including HMBC and COSY), where a homoserine moiety connected as an amide to C_12_ carboxylic acid terminated by a furan ring, and an esterified 2-methylhexanol residue were identified. ^13^C NMR, where coupling to phosphorus was observed, together with ^31^P NMR indicated the presence of a phosphate group. The geometry of the double bond was established as *E* by nOe experiments where effects were observed between H-3′ and H-5′ and CH_2_-2′ and Me-8′. The absolute configuration of homoserine was determined to be *D*((*R*)-2-amino-4-hydroxybutyric acid) by Marfey’s method. This absolute configuration suggests a microbial origin whereas the C_12_ carboxylic acid appears to be a trisnorsesquiterpene. Compound **270** showed mild anti HIV activity at a concentration of 0.2 μg/mL without showing any cytotoxicity.

**Figure 80:**
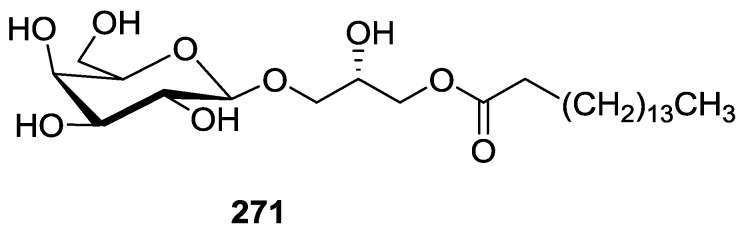
Structure of spongilipid **271**.

Pettit et al. [108] reported the isolation of spongilipid **271** from *S. cf. hispida* collected in the Republic of Singapore (Figure 80).

IR data indicated the presence of hydroxyl, ester and ether groups and preliminary ^1^H NMR analysis showed a long chain aliphatic unit; mass spectrometry indicated a C16 saturated aliphatic ester (palmitoyl group). Conversion to the pentaacetate allowed simplification of the spectra, identification of a β-linked sugar and of a glycerol unit. APT NMR experiment for carbon hybridization combined with COSY, HMQC and HMBC led to the assigned structures of both the pentaacetate and **271**. The structure was unequivocally confirmed and the absolute chemistry assigned by X-ray analysis. Compound **271** was active against *Enterococcus faecalis*. Under standard P388 techniques, the activity of **271** ranged from 2 to >100 mg/mL, presumably owing to difficulties caused by severe solubility problems and suspension formation in the cell media.

**Figure 81:**
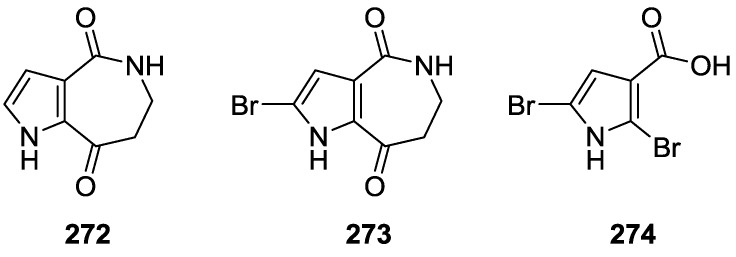
Structure of the alkaloids **272**–**274**.

Xu et al. [109] reported the isolation of three new alkaloids **272**–**274** from *S. obligue* collected in the South China Sea (Figure 81).

The structures were established by UV, IR, MS, 1D and 2D NMR and elemental analysis.

**Figure 82:**
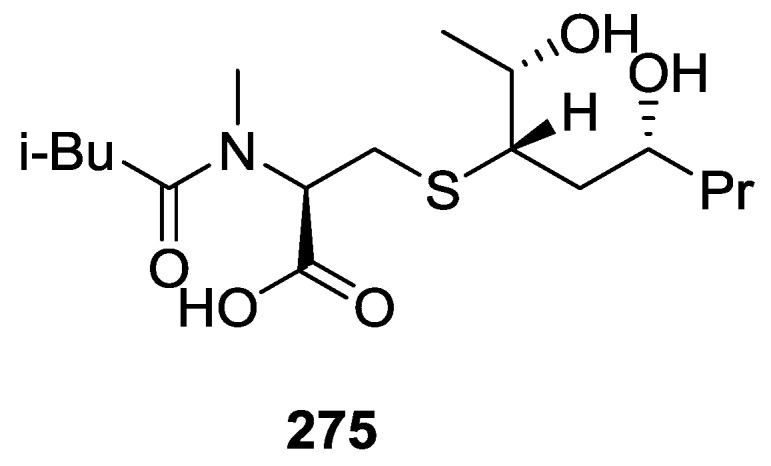
Structure of spongiacysteine **275**.

Kobayashi et al. [110] reported the isolation of spongiacysteine **275** from a *Spongia* sp. (Figure 82).

The structure was elucidated by spectroscopic analysis and the absolute stereostructure was established by total synthesis. **275** showed moderate antimicrobial activity against the rice blast fungus *Pyricularia oryzae*.

**Figure 83:**
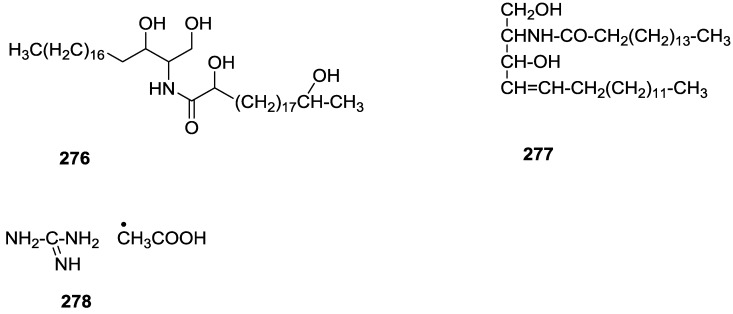
Structures of ceramide **276**, compound **277** and the guanidine acetic salt **278**.

Lin et al. [111] reported the isolation of a new ceramide **276**, together with **277** and the guanidine acetic salt **278** from *S. zimocca* subspecies *irregularia* (Ledenfeld) (Figure 83).

The structures were determined by spectroscopic methods.

**Figure 84:**
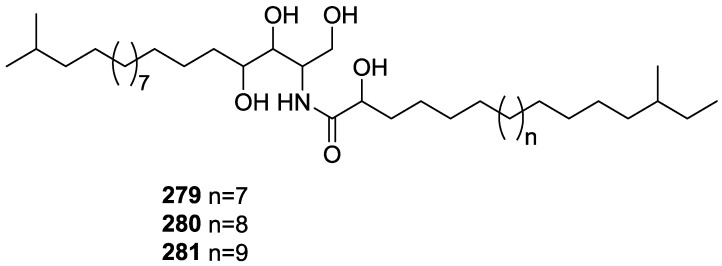
Structures of the ceramides 2-hydroxy-*N*-(1,3,4-trihydroxy-17-methyloctadecan-2-yl)-18-methylarachidamide **279**, 2-hydroxy-*N*-(1,3,4-trihydroxy-17-methyloctadecan-2-yl)-19-methyl-henicosanamide **280**, and 2-hydroxy-*N*-(1,3,4-trihydroxy-17-methyloctadecan-2-yl)-20-methyl-behenamide **281**.

Xu and Yang [112] reported the isolation of three new ceramides 2-hydroxy-*N*-(1,3,4-trihydroxy-17-methyloctadecan-2-yl)-18-methylarachidamide **279**, 2-hydroxy-*N*-(1,3,4-trihydroxy-17-methyloctadecan-2-yl)-19-methyl-henicosanamide **280** and 2-hydroxy-*N*-(1,3,4-trihydroxy-17-methyloctadecan-2-yl)-20-methyl-behenamide **281** from *S. suriganensis* collected in the South China Sea (Figure 84).

The structures were confirmed by spectroscopic analysis and hydrolysis.

**Figure 85:**
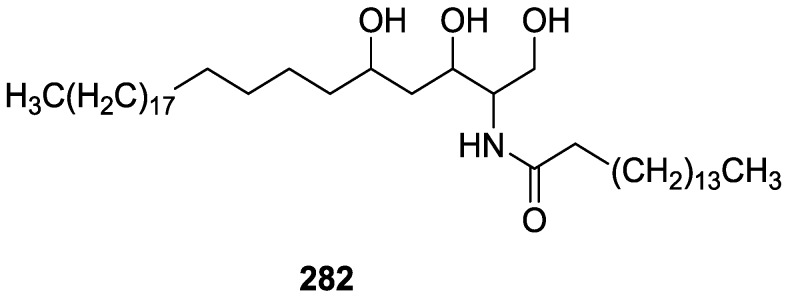
Structures of *N*-palmitoyl-heptacosane-1,3,5-triol **282**.

Guan and Zeng [113] reported the isolation of a ceramide, *N*-palmitoyl-heptacosane-1,3,5-triol **282**, from *Spongia* sp. collected in Hainan Province, China (Figure 85). The isolation of this compound was also reported in a Chinese Journal [114], where the presence of several other compounds (including sterols) is mentioned.

The structure was identified by IR, ^1^H, ^13^C and DEPT, together with FAB-MS data. The FAB-MS fragmentation showed peaks corresponding to fragments G and T, according to sphingolipid mass fragmentation patterns, which allowed the identification of the proposed structure. Further confirmation came from hydrolysis of **282**: the hexane extract furnished methyl palmitate as sole product.

**Figure 86:**
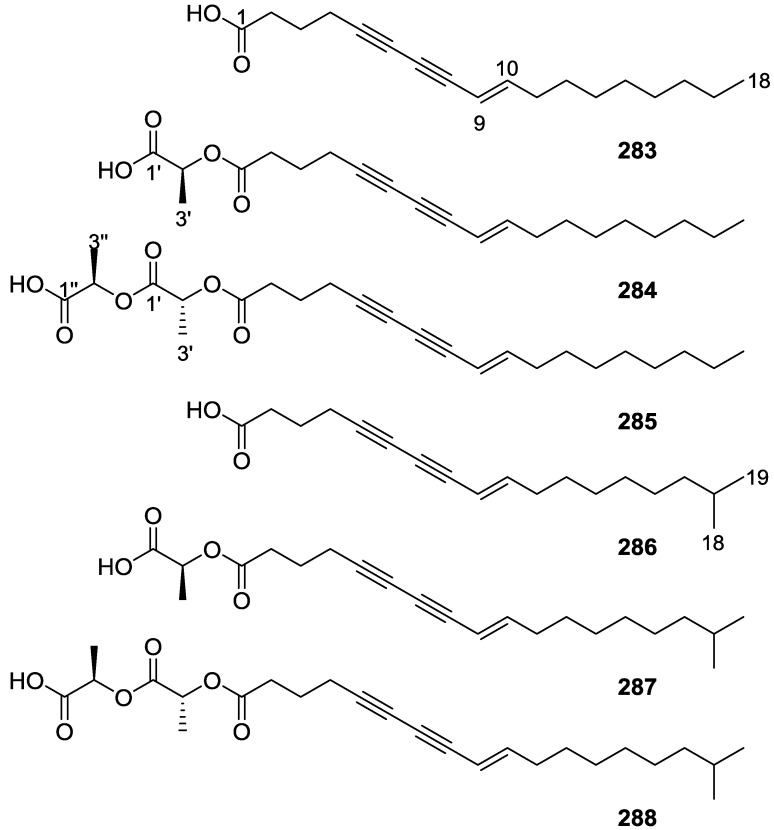
Structures of heterofibrins A1 **283**, A2 **284,** A3 **285**, B1 **286**, B2 **287** and B3 **288**.

Salim et al. [115] reported the isolation of heterofibrins A1 **283**, A2 **284,** A3 **285**, B1 **286**, B2 **287** and B3 **288** from *Spongia (heterofibria)* sp. collected in the Great Australian Bight (Figure 86).

For **283** UV indicated a diyne-ene moiety. The NMR indicated a carboxylic acid, two fully substituted acetylenes, an *E*-1,2-disubstituted olefin, a primary methyl and ten methylene carbons suggestive of an acyclic unbranched fatty acid. Analysis of 2D-NMR revealed a diagnostic correlation sequence from the carboxylic acid terminus, to the four acetylenic carbons, and from there to the olefinic methines, and on through the methylenes C-11, C-12 and C-13. The structure was thus assigned. Comparison of the data of **286** with that of **283** allowed the determination of the proposed structure. For **284,** comparison with **283** allowed the determination of an extra lactyl ester and a diastereotopic nature for both H-2 hydrogens. The 2′*S* absolute stereochemistry was assigned on the basis of the comparison of the value of the optical rotation with that of the synthesized (2′*S*)-lactyl linoleoate (the (2′*R*) isomer was also prepared). For **287,** comparison with **284** allowed the identification of the structure. The 2′*S* stereochemistry was assigned on the basis of the comparison of the optical rotation with those of **284** and (2′*S*)-lactyl linoleoate. Both **285** and **288** proved hard to purify. They were isolated as mixtures with 13-methylmyristic acid and palmitic acid, respectively. The ratios and the structure elucidation of each heterofibrin and associated fatty acid were confirmed by spectroscopic analysis and comparisons with model compounds (all the possible isomers of dilactyl linoleate). Only **283** and **286** inhibited the formation of lipid droplets (LD) in A431 fibroblast cells (up to 60% at 10 μM). All of the metabolites were non cytotoxic to the A431 fibroblast cells in the LD bioassay, or mammalian HeLa (cervical) and MDA-MB-231 (mammary epithelium) cancer cell lines (up to 30 μM). Likewise they were all non-cytotoxic to *Candida albicans*, *Pseudomonas aeruginosa* or *Escherichia coli* (IC_50_ values > 50 μM). They did display weak antibacterial activity against Gram positive bacterium *Bacillus subtilis* (10 < IC_90_ < 60 μM), while **283** and **286** displayed weak activity against *Staphylococcus aureus* (4 < IC_90_ < 45 μM). Further studies [116] on the effect of heterofibrin A1 **283** on the cellular storage of neutral lipids were undertaken [116]. Inhibition of LD biogenesis by heterofibrin A1 **283** was observed for A431 cells and AML12 hepatocytes. The activity was dose dependent with 20 μM inhibiting LD formation and triglyceride accumulation by 50% in the presence of 50 μM oleic acid. Compound **283** significantly reduced the intracellular accumulation of fatty acids and resulted in the formation of distinct fatty acid metabolites in both cultured cells and in embryos of the zebrafish *Danio rerio*.

**Figure 87:**
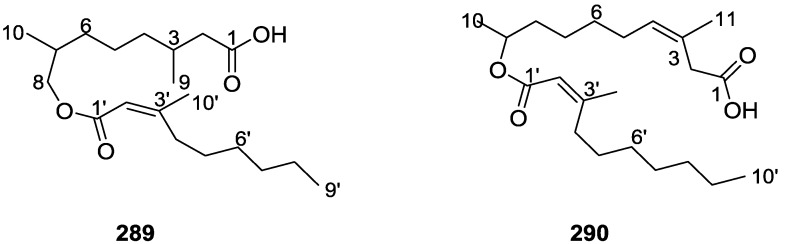
Structures of officinoic acid A **289** and officinoic acid B **290**.

From the already mentioned study of Manzo et al. [66] on *S. officinalis* collected in Sicily, officinoic acid A **289** and officinoic acid B **290** were isolated (Figure 87).

For **289** IR and ^13^C NMR allowed the identification of a carboxylic acid and an α,β-unsaturated ester. The ^13^C NMR spectrum also showed signals due to a trisubstituted double bond and 16 sp^3^ resonances including a signal due to an oxygenated methylene, which were consistent with an acyclic carbon skeleton. The ^1^H NMR spectrum showed, among others, four methyl signals. Analysis of ^1^H–^1^H COSY and HMBC identified the structure. The geometry of the double bond was established as *Z* by the value of the vinyl methyl carbon signal and nOe between Me-10′ and H-2′. The configurations at C-3 and C-7 were unassigned. The 2D NMR data of the methyl derivative was in accordance with the proposed structure. This derivative was submitted to methanolysis and the reaction mixture was analyzed by GC-MS where diagnostic molecular ion peaks for the two methyl esters were observed. Compound **290** was identified by NMR data analysis and comparison to **289**. The geometries of the double bonds were established as *Z* by the chemical shift values of the vinyl methyls and nOe (between Me-11 and H-4, and Me-11′ and H-2′). The configuration at C-9 was unassigned. Compound **290** was also converted to the methyl ester which was characterized by 2D NMR. Methanolysis of the derivative gave a reaction mixture analyzed by GC-MS, where again diagnostic molecular ion peaks for the two methyl esters were observed.

**Figure 88:**
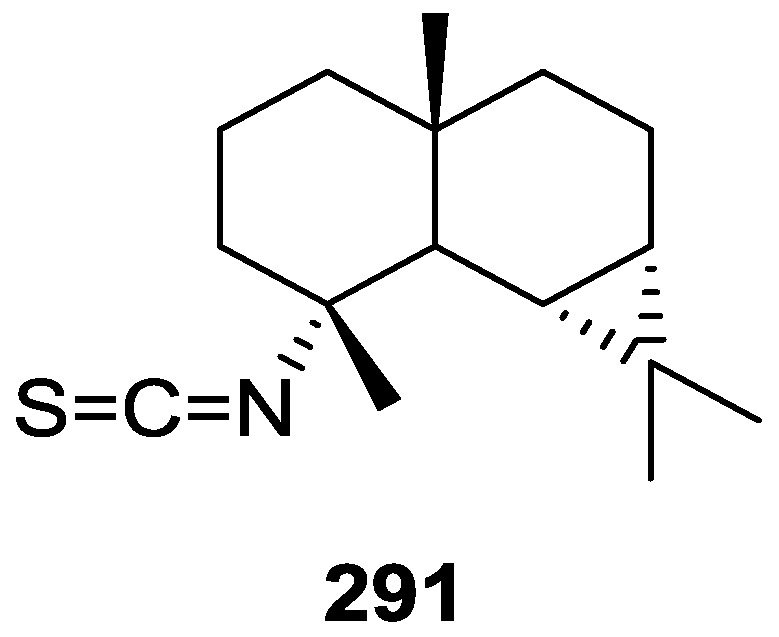
Structure of compound **291**.

From the already mentioned study of Ponomarenko et al. [39] on a *Spongia* ssp. (subgenus *Heterofibria*) collected in Northern Cook Island **291** was isolated (Figure 88).

The effects of **291** on the biosynthesis of nucleic acids and on the embryonic development of the sea urchin *Strongylocentrotus intermedius* were studied. The compound inhibited sea urchin embryo development at concentrations of 20 μg/mL and above and DNA biosynthesis at the dose of 10 μg/mL. The inhibitory effect of this compound may partly be explained by the inhibition of thymidine kinase activity. Compound **291** stimulated RNA synthesis in the developing sea urchin embryos.

Carballeira et al. [117] reported the total phospholipid fatty acid composition of *S. tampa*, collected in Puerto Rico. The most prominent phospholipid fatty acids were 5,9-pentacosadienoic acid and 5,9-hexacosadienoic acid. 9- and 16-pentacosenoic acids and 9-, 15- and 16-hexacosenoic acids were identified as trace amounts. The purified fraction of phosphatidylethanolamines was shown to contain C_14_–C_22_ fatty acids. The principal saturated fatty acids were tetradecanoic acid, hexadecanoic acid and octadecanoic acid. The principal fatty acids in the phosphatidylethanolamines were the monosaturated 9-octadecenoic acid and 13-docosenoic acid. 9-hexadecenoic acid and 11-octadecenoic acid were also found. The only dienoic acid present was 9,12-octadecadienoic acid. The positional distribution of the C_14_–C_22_ fatty acids in the phosphatidylethanolamines was examined. 9,12-octadecadienoic acid was shown to have no preference for the *sn*-1 or *sn*-2 positions in the phosphatidylethanolamines. The other saturated and unsaturated fatty acids with chains between 14 and 22 carbons long were also found to be equally distributed between the *sn*-1 and *sn*-2 positions of the phosphatidylethanolamines of this sponge.

Junqua et al. [118] reported the isolation and partial characterization of glycoconjugates from *S. officinalis* collected near Marseille. Two types of glycoconjugates were distinguished with respect to the size of their sugar moiety: type 1 contained 4%–7% carbohydrate, showed affinity for lectins and represent about 63% of total glycoconjugates; type 2 contained about 30% carbohydrate and some sulphate (about 2.7%) and were not bound to lectins. Type 1 glycoproteins had mol. wt. of 10, 16, 21 and 32 K. Glucuronic acid was present in all fractions together with galactose, mannose, glucose, fucose, arabinose and *N*-acetylglucosamine. Type 2 glycoconjugates had a much higher mol. wt. *N*-acetylglucosamine, *N*-acetylgalactosamine, galactose, mannose, fucose, arabinose and glucuronic acid were present. The aminoacid and carbohydrate composition revealed a striking similarity between sponge glycoproteins and structural glycoproteins isolated from vertebrate tissues.

## 9. Other Reports

**Figure 89:**
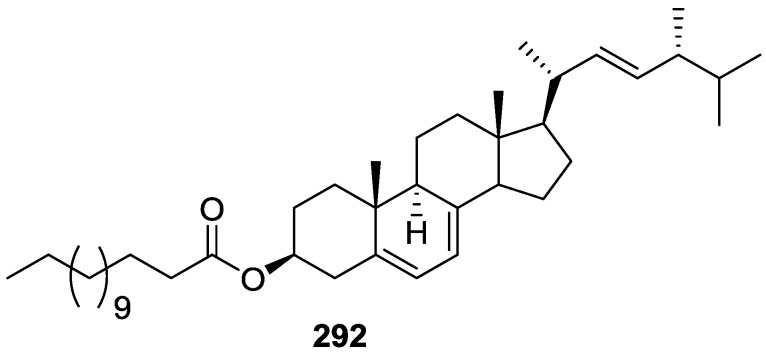
Structure of ergosteryl myristate **292**.

Noyer et al. [119] reported the intraspecific diversity of the Mediterranean *S. lamella*. The chemical profiles of seven populations spreading over 1200 km in the Western Mediterranean were obtained by SPE-HPLC-DAD-ELSD. Nitenin **117**, isonitenin **155**, dihydronitenin **118**, 12-episcalarin **166**, 12-*epi*-deoxoscalarin **165**, 12-*epi*-scalaradial **167**, 12,18-di-*epi*-scalaradial **168** and ergosteryl myristate **292** (Figure 89) were identified by comparison of the HPLC-MS spectra and ^1^H NMR with literature data.

**Figure 90:**
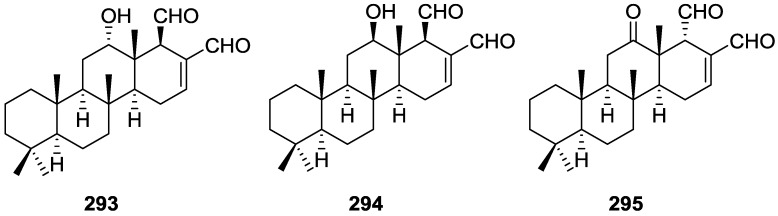
Structures of 12-deacetylscalaradial **293**, 12-deacetyl-12-*epi*-scalaradial **294**, and 12-deacetyl-18-*epi*-12-oxoscalaradial **295**.

Terem and Scheuer [120] reported the isolation of the known scalaradial **216** and 12-deacetylscalaradial **293** from an associated *S. oceania* and *Chromodoris youngbleuthi*, collected in O’ahu, Hawaii. From the nudibranch **216** was absent and **293**, and the new 12-deacetyl-12-*epi*-scalaradial **294** and 12-deacetyl-18-*epi*-12-oxoscalaradial **295** were isolated (Figure 90).

**216** and **293** were identified by comparison of their spectral data with literature compounds. For **295** the chemical shift of both H-11 suggested a 12-oxo functionality, confirmed by ^13^C NMR. Stereochemistry at C-18 was inferred by the *J* coupling value of the C-19 aldehyde proton, and confirmed by nOe, where irradiation of Me-25 enhanced the equatorial H-18. Analysis of the NMR data of **294** indicated a diastereomer of **293**. nOe studies were inconclusive for the assignment of the stereochemistry that had to be assigned by comparison of the spectral data of the acetylated derivative with literature compounds. The relative amounts of the **293**, **294** and **295** suggest that **293** is converted into the other metabolites by the nudibranch, which is supported by the absence of **294** and **295** in the sponge. Both **293** and **294** tasted more bitter than **295**. Fish feeding studies on *Tilapia* sp. in freshwater failed to quantify this finding although preliminary studies on filefishes showed on the basis of frequency counts that **293** was rejected more frequently than **295**.

**Figure 91:**
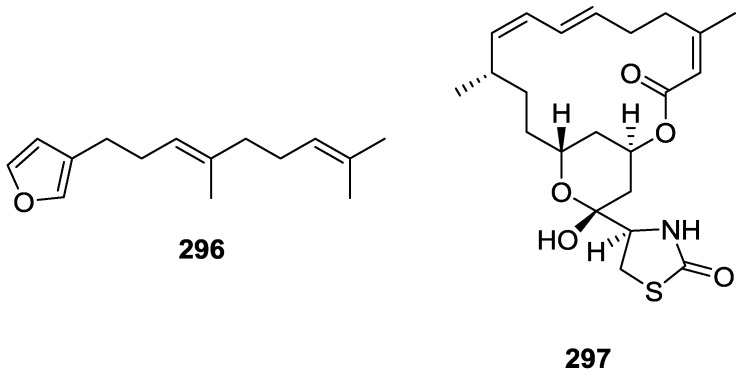
Structures of dendrolasin **296** and latrunculin A **297**.

Kakou and Crews [121] reported the isolation of dendrolasin **296** and latrunculin A **297** from an association of *S. mycofijijensis* and the nudibranch *Chromodoris lochi* (Figure 91).

Collections were from two distinct locations in the Fiji Islands. Although both organisms are associated separate extracts were performed and the content of each of the metabolites determined by ^13^C NMR. Compound **297** was completely toxic to Hep-2 and MA-104 cells at 0.072 μg/mL and 0.23 μg/mL, respectively. Compound **296** was completely toxic to Hep-2 at 24 μg/mL but inactive against MA-104 cells.

**Figure 92:**
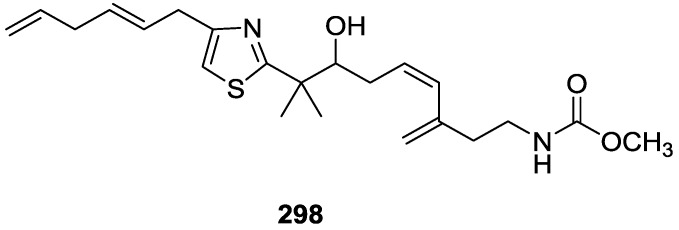
Structure of mycothiazole **298**.

Further studies [122] led to the recolection of the same pair of organisms in Vanuatu Islands, Australia. From the sponge, mycothiazole **298** was isolated (Figure 92).

It is noteworthy that ^13^C analysis of the extract showed the absence of dendrolasin A **296**. Mycothiazole **298** was identified by mass spectrometry and extensive NMR analysis (including spectra in three different solvents, ^1^H–^1^H COSY and ^1^H–^13^C COSY). For the determination of the connection site of the thiazole a study of the substituent increment shifts in ^13^C NMR and of the *J*_CH_ coupling values in substituted thiazole rings was performed, using literature data. A biosynthetic relation for mycothiazole **298** and lantrunculin B is proposed. Compound **298** was completely active at 50 μg/mL in an antihelminthic (in vitro) assay against *Nippostrongylus braziliensis*. It was deadly to mice at 10 mg/kg when injected intraperitonially, but no toxicity was seen by this route at 3 mg/kg.

**Figure 93:**
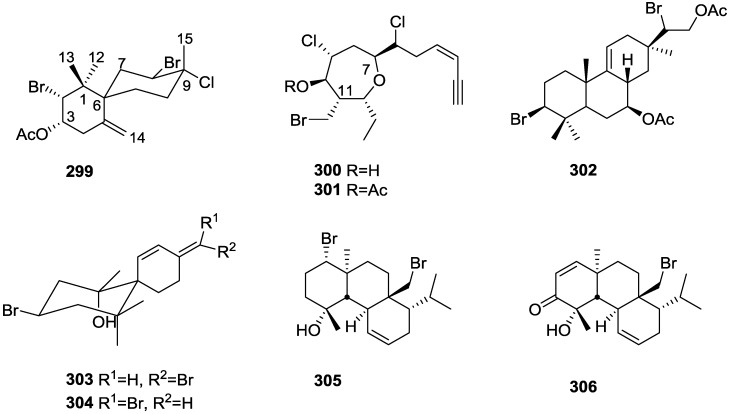
Structures of rogiolol acetate **299**, rogiolenyne B **300**, rogiolenyne C **301**, isopimarane **302**, chamigrene 4*E* **303**, chamigrene 4*Z* **304**, bromosphaerol **305**, and sphaerococcenol A **306**.

Three reports by Guella et al. [123,124,125] focused on the metabolites of *S. zimocca* and of the seaweed *Laurentia microcladia*. Both species grow in a narrow area off torrent of Il Rogiolo, south of Livorno. From the sponge the new rogiolol acetate **299**, rogiolenyne B **300** and rogiolenyne C **301** were isolated, together with the known isopimarane **302** (semisynthetic), chamigrene 4*E* **303**, chamigrene 4*Z* **304**, bromosphaerol **305**, sphaerococcenol A **306** and furospongin-1 **119** (Figure 93).

From the seaweed *L. microcladia* the deacetyl derivative of **299** was isolated, together with **300**, **303** and **304**. The presence of the di-deacetyl derivative of **302** could not be ascertained. The finding that **303** and **304**, as well as traces of **300**, are present ion both species strongly suggest that these metabolites are obtained by the sponge from the seaweed. A similar suggestion can be made about **305** and **306** since they are produced by the red seaweed *Sphaerococcus coronopifolius,* which also grows in the same area as *S. zimocca*. The authors suggest that *S. zimocca* either engulfs the seaweeds like the action of a solvent, or that it filters solid matrices (maybe algal spores) containing the compounds released by the seaweeds. The authors also suggest that compounds **299**, **301** and **302** are acetylated by the sponge. NMR data and MS data of both **299** and the deacetyl derivative, obtained by mild saponification, allowed the determination of the molecular formula and establishment of the presence of halogens. The β-chamigrene structure was deduced from NMR data where the exo methylidene group, the *gem*-dimethyl group and the spiro center could be identified. The location of the substituents was obtained by comparison with literature compounds and confirmed by COSY and ^1^H–^13^C correlation experiments. The relative configuration at the five carbon centers was derived from *J* coupling values and nOe. Further confirmation came from the addition of Eu(fod)_3_ to the deacetyl derivative, where all the paramagnetic shifts observed were in accordance with the proposed structure. Chemical transformation of the deacetyl derivative of **299** allowed the proposal of an absolute stereochemistry of (2*R*,3*S*,6*R*,8*R*,9*R*) (CD data) confirmed by chemical correlation of the deacetyl derivative with a known compound. For **300** and **301** mass spectrometry established the molecular formula and the presence of the chlorine and bromine atoms. Comparison of the NMR data with rogiolenyne A (epoxide ring in positions C-9/C-10, isolated from *L. microcladia*) identified both as chlorohydrin and acetyl chlorohydrin derivatives, respectively. Confirmation came from the transformation of **301** into rogiolenyne A by treatment with K_2_CO_3_/MeOH at room temperature. The location of the Cl atom at C-9 was inferred from COSY spectra. The pseudo-equatorial positions of H-9 and H-10 were inferred from the W-coupling of H-11/H-9 and H-10/one CH_2_-8, and the small *J* coupling values of H-9 and H-10. Confirmation came from nOe (one CH_2_-8/H-12 and H11/H-12. Eu(fod)_3_-induced deshielding of the H-12 resonance of **300** established the β-orientation of the OH group while the quasi-insensitive resonance of H-7 further supported the *trans*-relationship of the side chains at C-7 and C-12. The authors suggest that rogiolenyne A is transferred to the sponge where it undergoes epoxide opening by Cl^−^ anion to give **300** and **301**. Compounds **303** and **304** proved to be stable at low temperature (−15 °C) in the dark but to undergo interconversion when exposed to daylight at room temperature.

## 10. Biological Activity

In Table 7, a summary of the biological activities mentioned in the text is presented.

Several reports have appeared in the literature concerning the activity of extracts of *Spongia* sp.

Extracts of *S. barbara* collected in the Florida Keys significantly inhibited MAPK/ERK_1,2_ (mitogen-activated protein kinase-extracellular signal regulated kinase) activity (44% of control levels without altering cell survival) in cultured SW-13 human adrenal carcinoma cells. Results showed that ERK_2_ predominated over ERK_1_ and that the phosphorylated forms of these isozymes were strongly suppressed by active extracts [126].

Further studies [127] reported the effects of the extracts of the same sponge on cell cycle regulatory protein, cyclin B1; on cell cycle growth phase (sub G1/apoptosis, G1, S, and G2/M); and on cell survival in SW-13 human adrenal carcinoma cultures. A 70%–90% reduction in cyclin B1 levels was observed, together with a 10-fold increase in the percentage of cells entering apoptosis. During the same time the percentage of cells in G2/M was increased by 2 fold. Cell growth/survival studies also indicated a time-dependent decline in the percentage confluence of cell cultures exposed to the extracts.

A screening of the activity of *S. magellanica* (Thiele, 1905) against genotoxic biomarkers (mitotic index, cell proliferation kinetics and sister chromatid exchanges) was reported, although no activity was found [128].

Reports on the activity of extracts *S. officinalis* include:

- the screening against clinical isolates of bacteria including multi-drug resistant (MDR) strains and fungi, where activity against MDR strains of *Streptococcus pyogenes* and *Acinetobacter s*p. was observed [129];
- the anticonvulsant (using pentylenetetrazole seizure model) and analgesic (using writhing test in mice) activities of the extract (and its fractions) of the defensive secretion on the sponge, where analgesic activity in a dose dependent manner was observed for the extract [130];
- the evaluation of antiproliferative (A549—lung cell carcinoma, HCT15—colon cell carcinoma and MCF7—breast adenocarcinoma) and anti-inflammatory (carrageenan-induced rat paw edema) activities of the extract (and its semi-purified fractions) of the defensive secretion of the sponge [131] and of the sponge methanol extract (and its semi-purified fractions) [132], where significant antiproliferative activities and anti-inflammatory activity were observed, and
- the antiamoebic activity of extracts of *S. officinalis* var. *ceylonensis* against *Entamoeba histolytica*, where the alkaloids xestospongins and araguspongins where identified (by LCMS) as the major components of the active fraction [133].

## 11. Conclusions

The chemical composition of *Spongia* species has been studied in specimens obtained from different geographic areas. From these marine sponges a broad array of metabolites has been identified since 1971. The terpenic metabolites (sesqui-, di-, sesterpenes, and sterols) are the most representative compounds of the genera along macrolides, long chain lipid compounds and some alkaloids. More specifically we can verify that sesquiterpene quinones, C21 furanoterpenes, spongian diterpenes, scalarane sesterterpenes, sterols and secosterols are the most abundant skeletons biosynthesized by these marine organisms.

Some of the terpenic structures exhibit action on different cancer cell lines, are anti-HIV, anti-HSV1 and anti-HSV2, and immunomodulators, from a long list of assays described in literature. In general the description of long chain lipid compounds concerns their structure and they may be considered as part of the chemical fingerprint of *Spongia* genus. No activity assays are described for these compounds.

We highlight the most interesting activity encountered in *Spongia* metabolites that corresponds to the macrolide structure spongistatin 1 **263** that displays an extremely potent action against a broad array of highly chemoresistant tumour types in the range of nanograms. Macrolides are anticancer leads and spongistatin 1 **263** has been the subject of intense studies of synthetic strategies and anticancer mechanism of action. However the minute quantities in which this compound occurs from natural source and the complexity of the syntheses limit the progress of clinical studies.

Among the compounds of natural source that were identified as active and whose mechanism of action is somewhat effective on a given pathology there are few that reach the stage of clinical trials and even fewer come on the market for therapeutic purposes. Efforts are continuous to bring new lead structures to reach their role for the sake of human wellbeing. In this scenario marine organisms are an untold source of potent toxins that encourage scientists to break through the barriers to reach efficient therapeutic drugs.

## Figures and Tables

**Table 1:** Effect of compounds **107**–**110** on different sPLA_2_ activities ^a^.

Compound	*N. naja* Venom %I (10 μM)	Pancreas %I (10 μM)	Human Synovial %I (10 μM) IC50 (μM)	RAP^b^ + Zymosan %I (10 μM)	Bee Venom %I (10 μM) IC50 (μM)
**107**	0.5 ± 0.5	18.0 ± 8.1	40.1 ± 7.7 ^d^	17.1 ± 4.6	33.1 ± 6.0 ^c^
**108**	0.4 ± 0.4	14.2 ± 5.1	34.6 ± 5.8 ^d^	17.9 ± 4.2	32.2 ± 6.0 ^c^
**109**	3.1 ± 2.2	9.1 ± 3.5	40.4 ± 5.7 ^d^	30.9 ± 5.3 ^c^	36.2 ± 5.4 ^d^
**110**	0.0 ± 0.0	7.6 ± 4.0	48.2 ± 3.8 ^d^	19.6 ± 5.4	37.6 ± 6.5 ^c^
manoalide	17.0 ± 1.7 ^c^	32.3 ± 2.7 ^d^	93.2 ± 0.2 ^d^ 3.9	38.4 ± 0.5 ^d^	62.5 ± 3.8 ^d^ 7.5

^a^ Results show percentages of inhibition at 10 μM and IC_50_ (μM) values determined only for those compounds that reach 50% of inhibition. Mean ± S.E.M. (*n* = 6); ^b^ RAP: Rat air pouch PLA2; ^c^ *p* < 0.05; ^d^ *p* < 0.01.

**Table 2:** Cytotoxicity of compounds against tumor cell lines [77].

	IC_50_ (μg/mL)			
Compound	L1210	HeLa	A549	KB
**165**	2.1	22.5	29.4	16.2
**166**	13.2	26.0	23.7	18.5
**191**	>50	19.5	>50	>50
**192**	2.3	15.0	14.8	14.3
**193**	2.2	5.3	5.3	15.6
**194**	1.6	16.5	16.5	17.1
Mitomycin C	0.020	0.015	0.020	0.015

**Table 3:** Activity against FXR [79].

Compound	Inhibition FXR Transactivation IC_50_ (μM)	Cytotoxicity IC_50_ (μM) (CV-1 cell)
**165**	81.1	98.5
**198**	8.1	32.7
**199**	64.5	>100
**200**	24.8	86.9
**201**	25.3	29.2
*Z*-Guggulsterone	10.0	Not determined

**Table 4:** Activity against FXR [80].

Compound	Inhibition FXR Transactivation IC_50_ (μM)	Cytotoxicity IC_50_ (μM) (CV-1 Cell)
**166**	60.4	75.1
**192**	75.0	77.2
**194**	31.6	41.4
**202**	2.4	49.4
**203**	>100	>100
**204**	24.0	48.0
*E*-Guggulsterone	4.1	Not determined

**Table 5:** Effect of compounds **205** and **206** on different sPLA_2_ activities ^a^.

Compound	*N. naja* Venom %I (10 μM)	Pancreas %I (10 μM)	Human Synovial %I (10 μM) IC50 (μM)	RAP^b^ + Zymosan %I (10 μM)	Bee Venom %I (10 μM) IC50 (μM)
**205**	1.3 ± 0.8	14.3 ± 6.8	34.4 ± 6.5 ^d^	18.8 ± 3.2	37.1 ± 6.3 ^d^
**206**	8.7 ± 3.9	19.5 ± 3.6 ^d^	87.2 ± 2.1 ^d^ 5.8	25.6 ± 1.9 ^d^	5.4 ± 2.1
manoalide	17.0 ± 1.7 ^c^	32.3 ± 2.7 ^d^	93.2 ± 0.2 ^d^ 3.9	38.4 ± 0.5 ^d^	62.5 ± 3.8 ^d^ 7.5

^a^ Results show percentages of inhibition at 10 μM and IC_50_ (μM) values determined only for those compounds that reach 50% of inhibition. Mean ± S.E.M. (*n* = 6); ^b^ RAP: Rat air pouch PLA2; ^c^ *p* < 0.05; ^d^ *p* < 0.01.

**Table 6:** In vitro antiproliferative activity of spongidepsin **268** [82].

Cell Lines	268 IC_50_ (μM)	6-Mercaptopurine IC_50_ (μM)
J774.A1	0.56	0.003
HEK-293	0.66	0.007
WEHI-164	0.42	0.017

**Table 7:** Biological activities of *Spongia* sp. Metabolites.

Activity	Sesquiterpene Quinones	Diterpenes	C21 and Other Furano Terpenes	Sesterterpenes	Sterols	Macrolides	Miscellaneous Compounds
Artemia Salina		**53**,**54**,**63**,**74**,**75** [32] **41** [33]	**139**,**140** [58] **121**,**144**,**145**,**146** [61] **162** [32]	**165**,**169**,**172** [58]			
Anticancer (cytotoxicity and/or antiproliferative)	**6** [11] **8** [15,22] **12**,**13** [16] **14**,**15** [17] **21**,**22** [18]	**53**,**54**,**55** [27] **61** [29] **86** [36] **106** [41] **40**,**100** [48]	**156**,**157**,**158** [65] **296** [121]	**180**,**181**,**182**,**183** [73] **189,190** [67] **165**,**166**,**191**,**192**,**193**,**194** [77]	**259**,**260** [67]	**261**,**262** [93] **263** [94,95] **267** [82] **297** [121]	**271** [108] **283**,**284**,**285**,**286**,**287**,**288** [115]
Anticancer (other actions)	**8** [15] **14**,**15**,**16**,**17**,**18**,**19** [20]	**111** [43]		**180**,**181**,**182**, **183**,**184** [73] **165**,**192**,**194** [77]		**263** [103]	
Chemopreventive		**103**,**104** [40]					
DNA repair	**8** [15]						
Embryo development		**89**,**90**,**91**,**99**,**100**,**101**,**102** [39]					**291** [39]
Immunomodulatory		**90**,**94** [37]					
Immunosuppression				**209**,**210**,**211** [81]			
P-glycoprotein modulation					**247** [87] **247**,**248**,**249**,**250**,**251**,**252**,**253** [88]		
HSV1 or HSV2		**53**,**54**,**55** [27] **43** [48]					
HIV							**270** [107]
Microorganisms	**1**,**2** [9] **3**,**4**,**5** [10]	**48**,**49** [26]	**147** [62] **148** [63] **126** [66]				**271** [108] **275** [110] **283**,**284**,**285**,**286**,**287**,**288** [115]
Antihelminthic							**298** [122]
Predation/Defense			**139** [58] **143** [60]	**169**,**172** [58]			
Biofilm induction of *E. coli* PHL628			**123**,**124** [66]				
Hypercholesterolemia				**165**,**198**,**199**,**200**,**201** [79] **166**,**192**,**194**,**202**,**204** [80]			
Lipid metabolism							**283**,**286** [115] **283** [116]
Antiinflamatory				**205**,**206** [42]			
Antioxidant	**39** [21]						
Hemolytic activity	**6** [11]						
Smooth muscle paralyzing agent			**147** [62]				
Neurotransmission		**43**,**95**,**96**,**97**,**98** [38]					
Neurotrophic				**166**,**192**,**194**,**196**,**197** [78]			

## References

[1] Blunt J.W., Copp B.R., Keyzers R.A., Munroa M.H.G., Prinsepd M.R. (2015). Marine natural products. Nat. Prod. Rep..

[2] Perdicaris S., Vlachogianni T., Valavanidis A. (2013). Bioactive Natural Substances from Marine Sponges: New Developments and Prospects for Future Pharmaceuticals. Nat. Prod. Chem. Res..

[3] Pronzato R., Manconi R. (2008). Mediterranean Commercial Sponges: Over 5000 Years of Natural History and Cultural Heritage. Mar. Ecol..

[4] Voultsiadou E., Dailianis T., Antoniadou C., Vafidis D., Dounas C., Chintiroglou1 C.C. (2011). Aegean Bath Sponges: Historical Data and Current Status. Rev. Fish. Sci..

[5] Noyer C., Agell G., Pascual M., Becerro M.A. (2009). Isolation and Characterization of Microsatellite Loci from the Endangered Mediterranean Sponge *Spongia agaricina* (Demospongiae: Dictyoceratida). Conserv. Genet..

[6] Noyer C., Becerro M.A. (2012). Relationship between Genetic, Chemical, and Bacterial Diversity in the Atlanto-Mediterranean Bath Sponge *Spongia lamella*. Hydrobiology.

[7] Cunningham E., Dunne N., Walker G., Maggs C., Wilcox R., Buchanan F. (2010). Hydroxyapatite Bone Substitutes Developed via Replication of Natural Marine Sponges. J. Mater. Sci. Mater. Med..

[8] Fattorusso E., Minale L., Sodano G., Trivellone E. (1971). Isolation and Structure of Nitenin and Dihydronitenin, New Furanoterpenes from *Spongia nitens*. Tetrahedron.

[9] Urban S., Capon R.J. (1992). 5-*epi*-isospongiaquinone, A New Sesquiterpene/Quinone Antibiotic from an Australian Marine Sponge, *Spongia Hispida*. J. Nat. Prod..

[10] Capon R.J., Groves D.R., Urban S., Watson R.G. (1993). Spongiaquinone Revisited-Structural and Stereochemical Studies on Marine Sesquiterpene Quinones from a Southern Australian Marine Sponge, *Spongia* sp.. Aust. J. Chem..

[11] Utkina N.K., Denisenko V.A., Scholokova O.V., Virovaya M.V., Prokof’eva N.G. (2003). Cyclosmenospongine, a New Sesquiterpenoid Aminoquinone from an Australian Marine Sponge *Spongia* sp.. Tetrahedron Lett..

[12] Capon R.J., MacLeod J.K. (1987). A Revision of the Absolute Stereochemistry of Ilimaquinone. J. Org. Chem..

[13] Kondraki M.-L., Guyot M. (1987). Smenospongine: A Cytotoxic and Antimicrobial Aminoquinone Isolated from *Smenospongia* sp.. Tetrahedron Lett..

[14] Utkina N.K., Denisenko V.A., Scholokova O.V., Makarchenko A.E. (2003). Determination of the Absolute Stereochemistry of Cyclosmenospongine. J. Nat. Prod..

[15] Cao S., Gao Z., Thomas S.J., Hecht S.M., Lazo J.S., Kingston D.G.I. (2004). Marine Sesquiterpenoids that Inhibit the Lyase Activity of DNA Polymerase β. J. Nat. Prod..

[16] Takahashi Y., Tsuda M., Fromont J., Kobayashi J. (2006). Metachromins J and K, New Sesquiterpenoids from Marine Sponge *Spongia* Species. Heterocycles.

[17] Takahashi Y., Kubota T., Fromontb J., Kobayashi J. (2007). Metachromins L–Q, New Sesquiterpenoid Quinones with an Amino Acid Residue from Sponge *Spongia* sp.. Tetrahedron.

[18] Takahashi Y., Yamada M., Kubota T., Fromont J., Kobayashi J. (2007). Metachromins R–T, New Sesquiterpenoids from Marine Sponge *Spongia* sp.. Chem. Pharm. Bull..

[19] Takahashi Y., Kubota T., Yamamoto S., Kobayashi J. (2013). Inhibitory Effects of Metachromins L–Q and Its Related Analogs Against Receptor Tyrosine Kinases EGFR and HER2. Bioorg. Med. Chem. Lett..

[20] Takahashi Y., Kubota T., Kobayashi J. (2009). Nakijiquinones E and F, New Dimeric Sesquiterpenoid Quinones from Marine Sponge. Bioorg. Med. Chem..

[21] Utkina N.K., Denisenko V.A. (2011). Sesquiterpene Quinones from a Viet Nam Sea Sponge *Spongia* sp.. Chem. Nat. Compd..

[22] Kittiwisut S., Yuenyongsawad S., Mooberry S.L., Plubrukarn A. (2012). DNA Damage Initiated by Merosesquiterpenes from the Sponge *Spongia* sp.. Planta Med..

[23] Cimino G., Derosa D., Destefan S., Minale L. (1974). Isoagatholactone, a Diterpene of a New Structural Type from Sponge *Spongia officinalis*. Tetrahedron.

[24] Capelle N., Braekman J.C., Daloze D., Tursch B. (1980). Chemical Studies of Marine-Invertebrates .44. 3 New Spongian Diterpenes from *Spongia officinalis*. Bull. Des Soc. Chim. Belges.

[25] Cimino G., Morrone R., Sodano G. (1982). New Diterpenes from *Spongia officinalis*. Tetrahedron Lett..

[26] Gonzalez A.G., Estrada D.M., Martin J.D., Martin V.S., Perez C., Perez R. (1984). New Antimicrobial Diterpenes from a Sponge *Spongia officinalis*. Tetrahedron.

[27] Kohmoto S., McConnell O.J., Wright A., Cross S. (1987). Isospongiadiol, A Cytotoxic And Antiviral Diterpene From A Caribbean Deep-Water Marine Sponge, *Spongia* sp.. Chem. Lett..

[28] Hirsch S., Ashman Y.K. (1988). Spongialactone A, a New Spongian Diterpene from *Spongia arabica*. J. Nat. Prod..

[29] Gunasekera S.P., Schmitz F.J. (1991). New Spongian Diterpenoids from a Great Barrier Reef Sponge, *Spongia* sp.. J. Org. Chem..

[30] Searle P.A., Molinski T.F. (1994). Scalemic 12-Hydroxyambliofuran and 12-Acetoxyambliofuran, Five Furanoditerpenes and a Furanosesterterpene from *Spongia* sp.. Tetrahedron.

[31] Zubia E., Gavagnin M., Scognamiglio G., Cimino G. (1994). Spongiane and Ent.isocopalane Diterpenoids from the Mediterranean Sponge *Spongia zimocca*. J. Nat. Prod..

[32] Li C.-J., Schmitz F.J., Kelly-Borges M. (1998). New Diterpene Lactones from the Sponge *Spongia matamata*. J. Nat. Prod..

[33] Li C.-J., Schmitz F.J., Kelly-Borges M. (1999). Six New Spongian Diterpenes from the Sponge *Spongia matamata*. J. Nat. Prod..

[34] Mitchell S.S., Harper M.K., Faulkner D.J. (1999). Spongiabutenolides A–D: Minor γ-Hydroxybutenolide Diterpenoids from a Philippines *Spongia* sp.. Tetrahedron.

[35] Zeng L.M., Guan Z., Su J.Y., Feng X.L., Cai J.W. (2001). Two New Spongian Diterpene Lactones. Acta Chim. Sin..

[36] Su J.Y., Lin C.W., Zeng L.M., Yan S.J., Feng X.L., Cai J.W. (2003). Separation and Structure Determination of a New 19-nor-spongian Diterpenoid. Chem. J. Chin. Univ..

[37] Ponomarenko L.P., Kalinovsky A.I., Afiyatullov S.S., Pushilin M.A., Gerasimenko A.V., Krasokhin V.B., Stonik V.A. (2007). Spongian Diterpenoids from the Sponge *Spongia* (*Heterofibria*) sp.. J. Nat. Prod..

[38] Carroll A.R., Lamb J., Moni R., Hooper J.N.A., Quinn R.J. (2008). Spongian Diterpenes with Thyrotropin Releasing Hormone Receptor 2 Binding Affinity from *Spongia* sp.. J. Nat. Prod..

[39] Ponomarenko L.P., Terent’eva N.A., Krasokhin V.B., Kalinovsky A.I., Rasskazov V.A. (2011). Terpenoid Metabolites from *Spongia* spp. and Their Effects on Nucleic Acid Biosynthesis in Sea Urchin Eggs. Nat. Prod. Commun..

[40] Parrish S.M., Yoshida W.Y., Kondratyuk T.P., Park E.-J., Pezzuto J.M., Kelly M., Williams P.G. (2014). Spongiapyridine and Related Spongians Isolated from an Indonesian *Spongia* sp.. J. Nat. Prod..

[41] Pham A.T., Carney J.R., Yoshida W.Y., Scheuer P.J. (1992). Haumanamide, a Nitrogenous Spongian Derivative from *Spongia* sp.. Tetrahedron Lett..

[42] Marino S.D., Iorizzi M., Zollo F., Debitus C., Menou J.-L., Ospina L.F., Alcaraz M.J., Payá M. (2000). New Pyridinium Alkaloids from a Marine Sponge of the Genus *Spongia* with a Human Phospholipase A2 Inhibitor Profile. J. Nat. Prod..

[43] Mori D., Kimura Y., Kitamura S., Sakagami Y., Yoshioka Y., Shintani T., Okamoto T., Ojika M. (2007). Spongolactams, Farnesyl Transferase Inhibitors from a Marine Sponge: Isolation through an LC/MS-Guided Assay, Structures, and Semisyntheses. J. Org. Chem..

[44] Kazlauskas R., Murphy P.T., Wells R.J., Noack K., Oberhansli W.E., Schonholzer P. (1979). New Series of Diterpenes from Australian *Spongia* Species. Aust. J. Chem..

[45] Thompson J.E., Murphy P.T., Bergquist P.R., Evans E.A. (1987). Environmentally Induced Variation in Diterpene Composition of the Marine Sponge Rhopaloeides Odorabile. Biochem. Syst. Ecol..

[46] Puliti R., Mattia C.A. (1999). Ent-Isocopal-12-ene-15,16-dialdehyde from *Spongia officinalis*. Acta Crystallogr..

[47] Yong K.W.L., Garson M.J., Bernhardt P.V. (2009). Absolute Structures and Conformations of the Spongian Diterpenes Spongia-13(16),14-dien-3-one, Epispongiadiol and Spongiadiol. Acta Crystallogr. Sect. C.

[48] Betancur-Galvis L., Zuluaga C., Arnó M., González M.A., Zaragozá R.J. (2002). Cytotoxic Effect (on Tumor Cells) and in Vitro Antiviral Activity against Herpes Simplex Virus of Synthetic Spongiane Diterpenes. J. Nat. Prod..

[49] Cimino G., Stefano S.D., Minale L. (1971). Furospongin-1, a New C-21 Furanoterpene from the Sponges *Spongia officinalis* and *Hippospongia communis*. Tetrahedron.

[50] Cimino G., Stefano S.D., Minale L. (1972). Minor C-21 Furanoterpenes from the Sponges *Spongia officinalis* and *Hippospongia communis*. Tetrahedron.

[51] Cimino G., Stefano S.D., Minale L. (1972). Further Linear Furanoterpenes from Marine Sponges. Tetrahedron.

[52] Fontana A., Albarella L., Scognamiglio G., Uriz M., Cimino G. (1996). Structural and Stereochemical Studies of C-21 Terpenoids from Mediterranean Spongiidae Sponges. J. Nat. Prod..

[53] Kobayashi M., Chavakula R., Murata O., Sarma N.S. (1992). Marine Terpenes and Terpenoids. Part 16. Revised Structure of Marine Furanoterpene (+)-furospongin-1. J. Chem. Res..

[54] Cimino G., Stefano S.D., Minale L. (1974). Oxidized Furanoterpenes from the Sponge *Spongia officinalis*. Experientia.

[55] Kazlauskas R., Murphy P.T., Quinn R.J., Wells R.J. (1976). Tetradehydrofurospongin-1, a New C21 Furanoterpene from a Sponge. Tetrahedron Lett..

[56] Capon R.J., Ghisalberti E.L., Jefferies P.R. (1982). A New Furanoterpene from a *Spongia* sp.. Experientia.

[57] Kazlauskas R., Murphy P.T., Quinn R.J., Wells R.J. (1976). Two New Unsymetrically Oxygenated C21 furanoterpenes from a Sponge. Tetrahedron Lett..

[58] Walker R.P., Thompson J.E., Faulkner D.J. (1980). Sesterterpenes from *Spongia idia*. J. Org. Chem..

[59] Capon R.J., Jenkins A., Rooney F., Ghisalberti E.L. (2001). Structure Revision and Assignment of Absolute Stereochemistry of a Marine C21 Bisfuranoterpene. J. Nat. Prod..

[60] Tanaka J., Higa T. (1988). The Absolute Configuration of Kurospongin, a New Furanoterpene from a Marine Sponge, *Spongia* sp.. Tetrahedron.

[61] Giulio A.D., Rosa S.D., Vincenzo G.D. (1989). Terpenoids from the North Adriatic Sponge *Spongia officinalis*. J. Nat. Prod..

[62] Lumsdon D., Capon R.J., Thomas S.G., Beveridge A.A. (1992). A New Sesterterpene Tetronic Acid and a Pentaprenylated Para-Quinol from an Australian Marine Sponge, *Spongia* sp.. Aust. J. Chem..

[63] Urban S., Capon R.J. (1992). Cometins (A–C), New Furanosesterterpenes from an Australian Marine Sponge, *Spongia* sp.. Aust. J. Chem..

[64] Lenis L.A., Nunez L., Jimenez C., Riguera R. (1996). Isonitenin and Acetylhomoagmatine New Metabolites from the Sponges *Spongia officinalis* and *Cliona Celata* Collected at the Galician Coast (NW Spain). Nat. Prod. Lett..

[65] Garrido L., Zubía E., Ortega M.J., Salvá J. (1997). New Furanoterpenoids from the Sponge *Spongia officinalis*. J. Nat. Prod..

[66] Manzo E., Ciavatta M.L., Villani G., Varcamonti M., Sayem S.M.A., Soest R., Gavagnin M. (2011). Bioactive Terpenes from *Spongia officinalis*. J. Nat. Prod..

[67] Rueda A., Zubía E., Ortega M.J., Carballo J.L., Salvá J. (1998). New Metabolites from the Sponge *Spongia agaricina*. J. Nat. Prod..

[68] Cimino G., Stefano S.D., Minale L. (1973). Deoxoscalarin, a Further Sesterterpene with the Unusual Tetracyclic Carbon Skeleton of Scalarin, from *Spongia officinalis*. Experientia.

[69] Cimino G., Stefano S.D., Minale L., Trivellone E. (1977). 12-*epi*-Scalarin and 12-*epi*-Deoxoscalarin, Sesterterpenes from the Sponge *Spongia nitens*. J. Chem. Soc. Perkin Trans..

[70] Cimino G., Stefano S.D., Luccia A.D. (1979). Further Sesterterpenes from the Sponge *Spongia nitens*: 12-*epi*-scalaradial and 12,18-diepi-scalaradial. Experientia.

[71] Cimino G., Rosa S.D., Stefano S.D. (1981). Scalarolbutenolide, a New Sesterterpenoid from the Marine Sponge *Spongia nitens*. Experientia.

[72] Davis R., Capon R.J. (1993). Two New Scalarane Sesterterpenes-Isoscalarafuran-A and Isoscalarafuran-B, Epimeric Alcohols from a Southern Australian Marine Sponge, *Spongia hispida*. Aust. J. Chem..

[73] He H., Kulanthaivel P., Baker B.J. (1994). New Cytotoxic Sesterterpenes from the Marine Sponge *Spongia* sp.. Tetrahedron Lett..

[74] Conte M.R., Fatorrusso E., Lanzotti V., Magno S., Mayol L. (1994). Lintenolides, new pentacyclic bioactive sesterterpenes from the caribbean sponge *Cacospongia* cf. linteiformis. Tetrahedron.

[75] Lu Q., Faulkner D.J. (1997). Two New Sesterterpenoids and a New 9,11-Secosterol from *Spongia matamata*. J. Nat. Prod..

[76] Sakamoto K., Miyamot T., Amano H., Higuchi R., Komori T., Sasaki T. (1992). 34th Tennen Yuki Kagobutsu Toronkai Koen Yoshishu.

[77] Tsukamoto S., Miura S., Soest R.W.M.V., Ohta T. (2003). Three New Cytotoxic Sesterterpenes from a Marine Sponge *Spongia* sp.. J. Nat. Prod..

[78] Tokue T., Miura S., Kato H., Hirota H., Ohta T., Tsukamoto S. (2006). Neurotrophic Sesterterpenes Isolated from a Marine Sponge, *Spongia* sp.. Heterocycles.

[79] Nam S.-J., Ko H., Shin M., Ham J., Chin J., Kim Y., Kim H., Shin K., Choi H., Kang H. (2006). Farnesoid X-activated Receptor Antagonists from a Marine Sponge *Spongia* sp.. Bioorg. Med. Chem. Lett..

[80] Nam S.-J., Ko H., Ju M.K., Hwang H., Chin J., Ham J., Lee B., Lee J., Won D.H., Choi H. (2007). Scalarane Sesterterpenes from a Marine Sponge of the Genus *Spongia* and Their FXR Antagonistic Activity. J. Nat. Prod..

[81] Carr G., Raszek M., Soest R.V., Matainaho T., Shopik M., Holmes C.F.B., Andersen R.J. (2007). Protein Phosphatase Inhibitors Isolated from *Spongia irregularis* Collected in Papua New Guinea. J. Nat. Prod..

[82] Grassia A., Bruno I., Debitus C., Marzocco S., Pinto A., Gomez-Paloma L., Riccio R. (2001). Spongidepsin, a New Cytotoxic Macrolide from *Spongia* sp.. Tetrahedron.

[83] Aiello A., Ciminiello P., Fattorusso E., Magno S. (1988). 3,5-Dihydroxy-6-methoxycholest-7-enes from the Marine Sponge *Spongia agaricina*. J. Nat. Prod..

[84] Madaio A., Piccialli V., Sica D. (1989). New Polyhydroxysterols from the Dictyoceratid Sponges *Hippospongia communis*, *Spongia officinalis*, *Ircinia variabilis*, and *Spongionella gracilis*. J. Nat. Prod..

[85] Migliuolo A., Notaro G., Piccialli V., Sica D. (1990). New Tetrahydroxylated Sterols from the Marine Sponge *Spongia officinalis*. J. Nat. Prod..

[86] Migliuolo A., Piccialli V., Sica D., Giordano F. (1993). New Delta-8-5-alpha,6-alpha-epoxysterols and Delta-8(14)-5-alpha,6-alpha-epoxysterols from the Marine Sponge *Spongia officinalis*. Steroids.

[87] Aoki S., Yoshioka Y., Miyamoto Y., Higuchi K., Setiawan A., Murakami N., Chen Z.-S., Sumizawa T., Akiyama S., Kobayashi M. (1998). Agosterol A, a Novel Polyhydroxylated Sterol Acetate Reversing Multidrug Resistance from a Marine Sponge of *Spongia* sp.. Tetrahedron Lett..

[88] Aoki S., Setiawan A., Yoshioka Y., Higuchi K., Fudetani R., Chen Z.-S., Sumizawa T., Akiyama S., Kobayashi M. (1999). Reversal of Muitidrug Resistance in Human Carcinoma Cell Line by Agosterols, Marine Spongean Sterols. Tetrahedron.

[89] Chen Z.S., Aoki S., Komatsu M., Ueda K., Sumizawa T., Furukawa T., Okumura H., Ren X.Q., Belinsky M.G., Lee K. (2001). Reversal of Drug Resistance Mediated by Multidrug Resistance Protein (MRP) 1 by Dual Effects of Agosterol A on MRP1 Function. Int. J. Cancer.

[90] Migliuolo A., Piccialli V., Sica D. (1991). Structure Elucidation and Synthesis of 3β,6α-dihydroxy-9-oxo-9,11-seco-5α-cholest-7-en-11-al, a novel 9,11-secosterol from the Sponge *Spongia officinalis*. Tetrahedron.

[91] Migliuolo A., Piccialli V., Sica D. (1992). 2 New 9,11-Secosterols from the Marine Sponge *Spongia officinalis*. Synthesis of 9,11-seco-3-beta,6-alpha,11-Trihydroxy-5-alpha-cholest-7-en-9-one. Steroids.

[92] Adinolfi R., Migliuolo A., Piccialli V., Sica D. (1994). Isolation and Synthesis of a New 9,11-Secosterol from the Sponge *Spongia officinallis*. J. Nat. Prod..

[93] Quinoa E., Kakou Y., Crews P. (1988). Fijianolides, Polyketide Heterocycles from a Marine Sponge. J. Org. Chem..

[94] Pettit G.R., Cichacz Z.A., Gal F., Herald C.L., Boyd M.R., Schmidt J.M., Hooperlc J.N.A. (1993). Isolation and Structure of Spongistatin 1. J. Org. Chem..

[95] Pettit G.R., Cichacz Z.A., Gao F., Heralds C.L., Boyd M.R. (1993). Isolation and Structure of the Remarkable Human Cancer Cell Growth Inhibitors Spongistatins 2 and 3 from an Eastern Indian Ocean *Spongia* sp.. J. Chem. Soc. Chem. Commun..

[96] Pettit G.R., Cichacz Z.A., Gao F., Boyd M.R., Schmidt J.M. (1994). Isolation and Structure of the Cancer Cell Growth Inhibitor Dictyostatin 1. J. Chem. Soc. Chem. Commun..

[97] Kobayashi M., Aoki S., Sakai H., Kawazoe K., Kihara N., Sasaki T., Kitagawa I. (1993). Altohyrtin A, a Potent Anti-tumor Macrolide from the Okinawan Marine Sponge *Hyrfios alturn*. Tetrahedron Lett..

[98] Fusetani N., Shinoda K., Matsunaga S. (1993). Cinachyrolide A: A Potent Cytotoxic Macrolide Possessing Two Spiro Ketals from Marine Sponge *Cinachyra* sp.. J. Am. Chem. Soc..

[99] Pettit G.R., Herald C.L., Cichacz Z.A., Gao F., Boyd M.R., Christie N.D., Schmidt J.M. (1993). Antineoplastic Agents 293. the Exceptional Human Cancer Cell Growth Inhibitors Spongistatins 6 and 7. Nat. Prod. Lett..

[100] Kobayashi M., Aoki S., Gato K., Kitagawa I. (1996). Marine Natural Products. XXXVIII. Absolute stereostructures of Altohyrtins A, B, and C and 5-desacetylaltohyrtin A, a potent Cytotoxic Macrolides, from the Okinawan Marine Sponge *Hyrtios altum*. Chem. Pharm. Bull..

[101] Guo J., Duffy K.J., Stevens K.L., Dalko P.I., Roth R.M., Hayward M.M., Kishi Y. (1998). Total Synthesis of Altohyrtin A (Spongistatin 1): Part 1. Angew. Chem. Int. Ed..

[102] Hayward M.M., Roth R.M., Duffy K.J., Dalko P.I., Stevens K.L., Guo J., Kishi Y. (1998). Total Synthesis of Altohyrtin A (Spongistatin 1): Part 2. Angew. Chem. Int. Ed..

[103] Bai R.L., Cichacz Z.A., Herald C.L., Pettit G.R., Hamel E. (1993). Spongistatin-1, A Highly Cytotoxic, Sponge-Derived, Marine Natural Product that Inhibits Mitosis, Microtubule Assembly, and the Binding of Vinblastine to Tubulin. Mol. Pharmacol..

[104] Luduena R.F., Roach M.C., Prasad V., Pettit G.R., Cichacz Z.A., Herald C.L. (1995). Interaction of 3 Sponge-Derived Macrocyclic Lactone Polyethers (Spongistatin-3, Halistatin-1 and Halistatin-2) With Tubulin. Drug Dev. Res..

[105] Chen J., Forsyth C.J. (2004). Total Synthesis and Structural Assignment of Spongidepsin through a Stereodivergent Ring-Closing-Metathesis Strategy. Angew. Chem. Int. Ed..

[106] Ghosh A.K., Xu X. (2004). Assignment of Absolute Stereochemistry and Total Synthesis of (−)-Spongidepsin. Org. Lett..

[107] Kalidindi R.S., Yoshida W.Y., Palermo J.A., Scheuer P.J. (1994). Pokepola ester: A Phosphat Diester from a Maui Sponge. Tetrahedron Lett..

[108] Pettit G.R., Bond T.J., Herald D.L., Penny M., Doubek D.L., Williams M.D., Pettit R.K., Hooper J.N.A. (1997). Isolation and Structure of Spongilipid from the Republic of Singapore Marine Porifera S*pongia cf. hispida*. Can. J. Chem..

[109] Xu S.H., Cen Y.Z., Zeng L.M., Su J.Y. (2000). Isolation and Structural Determination of Heterocyclic Alkaloidal Compounds. Chin. J. Org. Chem..

[110] Kobayashi K., Shimogawa H., Sakakura A., Teruya T., Suenaga K., Kigoshi H. (2004). Spongiacysteine, a Novel Cysteine Derivative from Marine Sponge *Spongia* sp.. Chem. Lett..

[111] Lin C.W., Su J.Y., Zeng L.M., Wei W.X., Wei T.Y. (2005). Compounds Containing Nitrogen from *Spongia zimocca* Aubspecles Irregularia (Lendenfeld). Chin. J. Org. Chem..

[112] Xu S.H., Yang K. (2006). Three New Ceramides from the Sponge *Spongia suriganensis*. Chin. J. Org. Chem..

[113] Guan Z., Zeng L. (2010). A New Ceramide from a New Species of *Spongia* Sponge. Chem. Nat. Compd..

[114] Guan Z., Zeng L. (2010). Chemical constituents of a new species of *Spongia* sponge. Zhongguo Zhong Yao Za Zhi.

[115] Salim A.A., Rae J., Fontaine F., Conte M.M., Khalil Z., Martin S., Partonb R.G., Capon R.J. (2010). Heterofibrins: Inhibitors of Lipid Droplet Formation from a Deep-water Southern Australian Marine Sponge, *Spongia* (*Heterofibria*) sp.. Org. Biomol. Chem..

[116] Rae J., Fontaine F., Salim A.A., Lo H.P., Capon R.J., Parton R.G., Martin S. (2011). High-Throughput Screening of Australian Marine Organism Extracts for Bioactive Molecules Affecting the Cellular Storage of Neutral Lipids. PLoS ONE.

[117] Carballeira N.M., Emiliano A., Morales R. (1994). Positional Distribution of Octadecadienoic Acids in Sponge Phosphatidylethanolamines. Lipids.

[118] Junqua S., Lemonnier M., Robert L. (1981). Glycoconjugates from *Spongia officinalis* (Phylum Porifera). Isolation, Fractionation by Affinity-Chromatography on Lectins and Partial Characterization. Comp. Biochem. Physiol. B Biochem. Mol. Biol..

[119] Noyer C., Thomas O.P., Becerro M.A. (2011). Patterns of Chemical Diversity in the Mediterranean Sponge *Spongia lamella*. PLoS ONE.

[120] Terem B., Scheuer P.J. (1986). Scalaradial Derivatives from the Nudibranch *Chromodoris youngbleuthi* and the Sponge *Spongia oceania*. Tetrahedron.

[121] Kakou Y., Crew P. (1987). Dendrolasin and Latrunculin A from the Fijan Sponge S*pongia mycofijiensis* and an Associated Nudibranch *Chromodoris lochi*. J. Nat. Prod..

[122] Crews P., Kakou Y., Quinoa E. (1988). Mycothiazole, a Polyketide Heterocycle from a Marine Sponge. J. Am. Chem. Soc..

[123] Guella G., Mancini I., Chiasera G., Pietra F. (1990). Rogiolol Acetate: A Novel β-chamigrene-type Sesquiterpene Isolated from a Marine Sponge. Helv. Chim. Acta.

[124] Guella G., Pietra F. (1991). Rogiolenyne-A, Rogiolenyne-B, and Rogiolenyne-C—The first Branched Marine C-15 Acetogenins. Isolation from the Red Seaweed *Laurencia microcladia* or the Sponge *Spongia-zimocca* of Il-Rogiolo. Helv. Chim. Acta.

[125] Guella G., Mancini I., Pietra F. (1992). C-15 Acetogenins and Terpenes of the Dictyoceratid Sponge *Spongia-zimocca* of Il-Rogiolo: A Case of Seaweed-Metabolite Transfer to, and Elaboration within, a Sponge?. Comp. Biochem. Phys. B Comp. Biochem..

[126] Brown J.W., Kesler C.T., Neary J.T., Fishman L.M. (2001). Effects of Marine Sponge Extracts on Mitogen-Activated Protein Kinase (MAPK/ERK_1,2_) Activity in SW-13 Human Adrenal Carcinoma Cells. Toxicon.

[127] Brown J.W., Cappell S., Perez-Stable C., Fishman L.M. (2004). Extracts from Two Marine Sponges Lower Cyclin B1 Levels, Cause a G2/M Cell Cycle Block and Trigger Apoptosis in SW-13 Human Adrenal Carcinoma Cells. Toxicon.

[128] Bartolotta S.A., Scuteri M.A., Hick A.S., Palermo J., Brasco M.F.R., Hajdu E., Mothes B., Lerner C., Campos M., Carballo M.A. (2009). Evaluation of Genotoxic Biomarkers in Extracts of Marine Sponges from Argentinean South Sea. J. Exp. Mar. Biol. Ecol..

[129] Devi P., Wahidulla S., Kamat T., D’Souza L. (2011). Screening Marine Organisms for Antimicrobial Activity against Clinical Pathogens. Indian J. Mar. Sci..

[130] Dellai A., Mansour H.B., Clary-Laroche A., Deghrigue M., Bouraoui A. (2012). Anticonvulsant and Analgesic Activities of Crude Extract and Its Fractions of the Defensive Secretion from the Mediterranean Sponge, *Spongia officinalis*. Cancer Cell Int..

[131] Dellai A., Deghrigue M., Laroche-Clary A., Masour H.B., Chouchane N., Robert J., Bouraoui A. (2010). Anti-inflammatory and Antiproliferative Activities of Crude Extract and Its Fractions of the Defensive Secretion From the Mediterranean Sponge, *Spongia officinalis*. Drug Dev. Res..

[132] Dellai A., Deghrigue M., Laroche-Clary A., Masour H.B., Chouchane N., Robert J., Bouraoui A. (2012). Evaluation of Antiproliferative and Anti-inflammatory Activities of Methanol Extract and Its Fractions from the Mediterranean Sponge. Cancer Cell Int..

[133] Lakshmi V., Ghosal S. (2014). Antiamoebic Activity of Marine Sponge *Spongia officinalis* var. ceylonensis Dendy. Bangladesh Pharm. J..

